# The α_2_δ-like Protein Cachd1 Increases N-type Calcium Currents and Cell Surface Expression and Competes with α_2_δ-1

**DOI:** 10.1016/j.celrep.2018.10.033

**Published:** 2018-11-06

**Authors:** Shehrazade Dahimene, Karen M. Page, Ivan Kadurin, Laurent Ferron, Dominique Y. Ho, Gareth T. Powell, Wendy S. Pratt, Stephen W. Wilson, Annette C. Dolphin

**Affiliations:** 1 Department of Neuroscience, Physiology and Pharmacology, Division of Biosciences, University College London, London WC1E 6BT, UK; 2 Department of Cell and Developmental Biology, Division of Biosciences, University College London, London WC1E 6BT, UK

**Keywords:** voltage-gated calcium channel, Cachd1, cell surface expression, interaction site, cache domain

## Abstract

Voltage-gated calcium channel auxiliary α2δ subunits are important for channel trafficking and function. Here, we compare the effects of α2δ-1 and an α2δ-like protein called Cachd1 on neuronal N-type (Ca_V_2.2) channels, which are important in neurotransmission. Previous structural studies show the α2δ-1 VWA domain interacting with the first loop in Ca_V_1.1 domain-I via its metal ion-dependent adhesion site (MIDAS) motif and additional Cache domain interactions. Cachd1 has a disrupted MIDAS motif. However, Cachd1 increases Ca_V_2.2 currents substantially (although less than α2δ-1) and increases Ca_V_2.2 cell surface expression by reducing endocytosis. Although the effects of α2δ-1 are abolished by mutation of Asp122 in Ca_V_2.2 domain-I, which mediates interaction with its VWA domain, the Cachd1 responses are unaffected. Furthermore, Cachd1 co-immunoprecipitates with Ca_V_2.2 and inhibits co-immunoprecipitation of α2δ-1 by Ca_V_2.2. Cachd1 also competes with α2δ-1 for effects on trafficking. Thus, Cachd1 influences both Ca_V_2.2 trafficking and function and can inhibit responses to α2δ-1.

## Highlights

• Cachd1 enhances Ca_V_2.2 currents and increases Ca_V_2.2 surface expression
• Effects of Cachd1 are not prevented by mutation in Ca_V_2.2 VWA interaction site
• The effects of α_2_δ-1 are prevented by the same mutation in Ca_V_2.2
• Cachd1 competes with α_2_δ-1 for its effects on Ca_V_2.2

## Graphical Abstract

**Figure:**
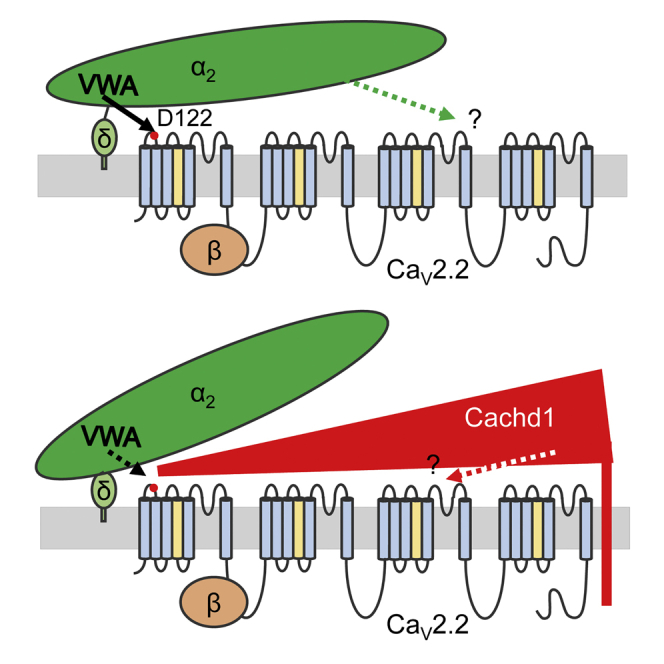

## Teaser

Dahimene et al. examine the role of Cachd1, a protein with similarity to the auxiliary α_2_δ subunits of voltage-gated calcium channels. They find that Cachd1 increases N-type calcium currents substantially despite having a disrupted VWA interaction domain. Cachd1 also enhances channel trafficking and inhibits responses to α_2_δ-1.

## Introduction

Voltage-gated calcium (Ca_V_) channels are key constituents of excitable cells, including muscles, neurons, and secretory cells, and are essential for their function (for a review, see Zamponi et al., 2015). The neuronal N-type (Ca_V_2.2) and P/Q-type (Ca_V_2.1) channels are critical for presynaptic release of neurotransmitters (for a review, see Nanou and Catterall, 2018), with N-type calcium channels playing a particularly important role in primary afferent neurotransmission involving pain pathways (for a review, see McGivern and McDonough, 2004). Ca_V_ α1 subunits form the pore of the channels, determining their main biophysical and pharmacological properties (Zamponi et al., 2015), but the associated β and α_2_δ proteins represent auxiliary subunits that are important contributors to the trafficking and biophysical properties of the channel complexes (Gurnett et al., 1996, Leung et al., 1987, Pragnell et al., 1994, Takahashi et al., 1987). The β subunits increase Ca_V_ currents by binding to the intracellular I-II linker (Pragnell et al., 1994), promoting folding (Van Petegem et al., 2004), hyperpolarizing current activation (Stea et al., 1993), preventing polyubiquitination (Page et al., 2016), and inhibiting proteasomal degradation (Altier et al., 2011, Waithe et al., 2011).

By contrast, the mechanism by which the α_2_δ subunits increase trafficking and function of channel complexes is less well understood (Cantí et al., 2005, Cassidy et al., 2014, Ferron et al., 2018, Kadurin et al., 2016, Savalli et al., 2016). The α_2_δ-1 subunit, in combination with neuronal calcium channels, is the therapeutic target for gabapentinoid drugs, used for the alleviation of neuropathic pain conditions and as an add-on therapy in certain epilepsies (Field et al., 2006), and it is therefore important to understand its mechanism of action. The α_2_δ proteins undergo several post-translational processing steps, including N-glycosylation, proteolytic cleavage into α_2_ and δ (De Jongh et al., 1990, Ellis et al., 1988, Jay et al., 1991), and glycosyl-phosphatidylinositol (GPI) anchoring (Davies et al., 2010).

The recent structure of the skeletal muscle Ca_V_1.1 complex (Wu et al., 2016) has revealed a complex interaction of α_2_δ-1 with several extracellular loops in domains I-III of Ca_V_1.1. In the present study, we have taken advantage of the insights provided by this structure to probe the role of the von Willebrand factor A (VWA) domain and investigate whether there is a role for other α_2_δ domains in Ca_V_ channel function. In previous studies, by mutating the metal ion-dependent adhesion site (MIDAS) motif in the VWA domain of α_2_δ subunits, we have shown that the VWA domains of both α_2_δ-1 and α_2_δ-2 are key to promoting calcium channel trafficking and function (Cantí et al., 2005, Cassidy et al., 2014, Hoppa et al., 2012). The structure confirms the interaction of the MIDAS motif with the Ca_V_1.1 α1 subunit (Wu et al., 2016). However, we also found that mutating the MIDAS motif reduced the trafficking of α_2_δ-1 itself when it was expressed alone (Cassidy et al., 2014). In the present study, we have therefore taken the reciprocal step of mutating the residue in Ca_V_2.2 with which α_2_δ-1 is predicted to bind to examine whether other regions, such as their Cache domains, play a role in promoting Ca_V_2.2 trafficking and function. The Cache domains in α_2_δ-1, which have homology to domains in bacterial chemotaxis receptors (Anantharaman and Aravind, 2000), have also been shown to interact with the Ca_V_1.1 α1 subunit (Wu et al., 2016). We have compared the effect of α_2_δ-1 with that of Cachd1, identified bioinformatically to be related to α_2_δ proteins (Whittaker and Hynes, 2002). Cachd1 has a VWA domain with a disrupted MIDAS motif but retains multiple predicted Cache domains. Surprisingly, we found that expression of Cachd1 increased both Ca_V_2.2 currents and cell surface trafficking in both cell lines and neurons. By contrast, expression of Cachd1 did not increase the closely related Ca_V_2.1 currents, indicating that this effect shows specificity for certain calcium channels. Furthermore, Cachd1 competed with α_2_δ-1 for binding to Ca_V_2.2 and for its functional effects and can therefore inhibit responses to α_2_δ-1.

## Results

### Disruption of the Interaction Site between Ca_V_2.2 and the α_2_δ-1 VWA Domain Prevents the Interaction between α_2_δ-1 and Ca_V_2.2

**Figure 1:**
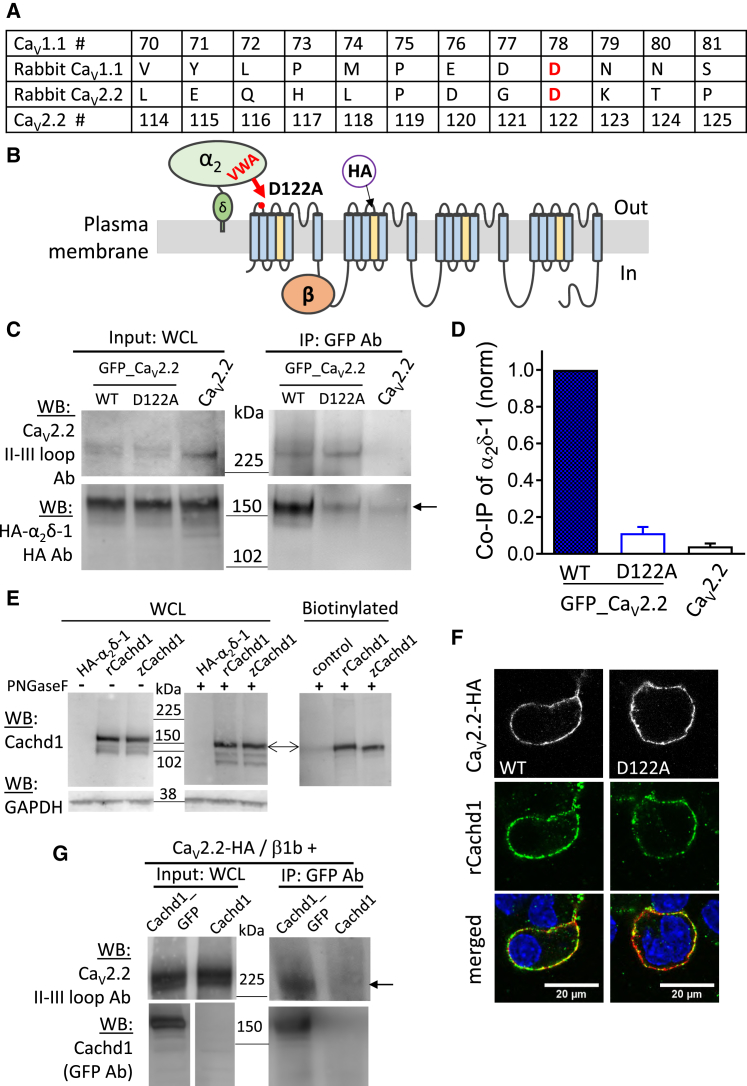
Effect of D122A Mutation in Ca_V_2.2 on Interaction with α_2_δ-1 and Cachd1 (A) Sequence alignment of the VWA domain interaction site on Ca_V_1.1 in comparison with the rabbit Ca_V_2.2 used in this study, showing the position of D122 in the first extracellular loop of Ca_V_2.2. Residue numbering is shown (#). (B) Diagram of the putative Ca_V_2.2 interaction site with the VWA domain of α_2_δ-1, showing the position of the D122A mutation and the HA epitope tag. (C) IP of GFP_Ca_V_2.2, and co-immunoprecipitation (coIP) of α_2_δ-1. WCL input (left) and IP (right) for WT and D122A mutant GFP_Ca_V_2.2 and untagged Ca_V_2.2 control (top) and for HA-tagged α_2_δ-1 (bottom). IP was performed with GFP Ab and pulled down both WT and D122A GFP_Ca_V_2.2 (top right). CoIP of HA-tagged α_2_δ-1 is shown in at the bottom right (arrow). (D) Quantification of coIP of α_2_δ-1 with WT GFP_Ca_V_2.2 (solid blue bar) compared with D122A GFP_Ca_V_2.2 (open blue bar) and control Ca_V_2.2 (open black bar); mean ± SEM of 5 experiments. (E) Western blot using Cachd1 Ab of WCL from tsA-201 cells transfected with α_2_δ-1 as a control (lane 1), rCachd1 (lane 2), and zCachd1 (lane 3). Left: prior to deglycosylation with PNGase F. Center: after deglycosylation. Bottom: glyceraldehyde 3-phosphate dehydrogenase (GAPDH) loading control. Right: a separate experiment after cell surface biotinylation and deglycosylation; the control here was untransfected cells. The arrow indicates a major Cachd1 band. (F) Representative confocal images of N2A cells expressing Ca_V_2.2 HA WT (left) or D122A (right) with β1b and rCachd1. Cells were not permeabilized and incubated with rat anti-HA and rabbit anti-Cachd1 Abs for 1 hr to show extracellular HA staining on the plasma membrane (top row, white) and Cachd1 (center row, green). Merged images (with HA in red and co-localization in yellow) are shown at the bottom; DAPI was used to stain the nuclei (blue). Scale bars, 20 μm. (G) IP of Cachd1_GFP and coIP of Ca_V_2.2. Shown are WCL input (left) and IP (right). Top: Ca_V_2.2. Bottom: zCachd1_GFP (left lane) and untagged zCachd1 (right lane; both lanes are from the same blot). IP was performed with GFP Ab and pulled down both Cachd1_GFP (bottom) and Ca_V_2.2 (top right, arrow). Lack of coIP of Ca_V_2.2 with untagged Cachd1 is shown in the right lane. Data are representative of n = 6 experiments. zCachd1 expression in WCL is confirmed in Figure S1C.

In previous studies, we found that mutation of the MIDAS motif in α_2_δ-1 and α_2_δ-2 prevented the ability of these proteins to traffic Ca_V_2 channels and abolished the increase in Ca_V_1 and Ca_V_2 currents, normally seen with wild-type (WT) α_2_δ-1 and α_2_δ-2 (Cantí et al., 2005, Cassidy et al., 2014, Hoppa et al., 2012). However, trafficking of the α_2_δ-1 MIDAS mutant alone to the cell surface was also impaired (Cassidy et al., 2014), and our data indicate that α_2_δ-1 also interacts with the trafficking protein LRP1 via its VWA domain (Kadurin et al., 2017). Therefore, in the present study, we took advantage of the recently described structure of the skeletal muscle calcium channel complex (Wu et al., 2016) and mutated the residue in Ca_V_2.2 likely to coordinate the divalent cation together with the MIDAS interaction site of α_2_δ-1. The structure of Ca_V_1.1 shows this to be residue D78, which is in the first extracellular loop of domain I; it corresponds by alignment to D122 in Ca_V_2.2 (Figures 1A and 1B). This residue was mutated to uncharged alanine to disrupt the interaction with α_2_δ-1. D122A Ca_V_2.2 was expressed at the same level as WT Ca_V_2.2 in tsA-201 cells in the presence of β1b and α_2_δ-1 (Figure 1C). As we found previously (Kadurin et al., 2016), Ca_V_2.2 showed robust co-immunoprecipitation with α_2_δ-1 (Figures 1C and 1D). In contrast, D122A Ca_V_2.2 exhibited only very weak co-immunoprecipitation (coIP) with α_2_δ-1 (Figures 1C and 1D), confirming a key role for D122 in this interaction.

### The α_2_δ Homolog Cachd1 Is Expressed on the Cell Surface and Interacts with Ca_V_2.2

The cryoelectron microscopy (cryo-EM) structure of Ca_V_1.1 shows that α_2_δ-1 has four Cache domains (Anantharaman and Aravind, 2000, Wu et al., 2016), and there are interactions of the α1 subunit with these domains as well as with the VWA domain (Wu et al., 2016). The α_2_δ-like protein Cachd1 contains Cache domains, similar to the α_2_δ subunits, but its VWA domain has a highly disrupted MIDAS motif (Whittaker and Hynes, 2002). Indeed, in a preliminary report, Cachd1 was found to have no effect on Ca_V_2.2 currents (Soubrane et al., 2012). Because our experiments also suggest that the VWA domain has a dominant role in mediating the effects of α_2_δ-1, we decided to investigate whether Cachd1 showed any residual functional effect on Ca_V_2.2 function.

We initially used a construct encoding zebrafish Cachd1 (zCachd1) that had been generated in a study to identify genes underlying particular nervous system development phenotypes (H. Stickney, A. Faro, G.T.P., and S.W.W., unpublished data). We subsequently confirmed our results with the rat construct rCachd1. There is very high sequence conservation, the two proteins being 85.6% identical at the amino acid level. Using a polyclonal antibody (Ab) raised against the predicted extracellular domain of zCachd1, which also recognizes human CACHD1 (G.T.P., G.J. Wright, and S.W.W., unpublished data), we observed a major band of the predicted molecular weight (MW) in whole-cell lysate (WCL) of tsA-201 cells transfected to express either zCachd1 or rCachd1 (Figure 1E). Cachd1 is predicted to be an N-glycosylated protein (Figure S1A). For rCachd1, the MW was ∼168 kDa when glycosylated and ∼148 kDa following deglycosylation with N-Glycosidase F (PNGase F), indicating that it has up to 7 N-glycosylation sites (Figure 1E), agreeing with the predicted number (Figure S1A). The glycosylation pattern is also compatible with the prediction that Cachd1 is a type I membrane protein (Figure S1A), in contrast to the GPI-anchored α_2_δ proteins (Davies et al., 2010). In addition to the major Cachd1 protein band, two lower MW minor bands were observed. For rCachd1, these were ∼148 and ∼137 kDa, reduced to ∼133 and ∼119 kDa following deglycosylation (Figure 1E). Similar results were found for zCachd1 (Figure 1E). Cell surface biotinylation indicated that the major band was the species on the plasma membrane (Figure 1E), suggesting that membrane-associated Cachd1 does not undergo post-translational proteolytic processing, unlike α_2_δ proteins.

To determine whether Cachd1 was co-localized on the cell surface with Ca_V_2.2, we expressed the proteins in N2A or tsA-201 cells and imaged their localization. We found that both rCachd1 (Figure 1F) and zCachd1 (Figure S1B) were present on the cell surface, together with either WT Ca_V_2.2 or D122A Ca_V_2.2 and β1b. Partial co-localization of Cachd1 with Ca_V_2.2-hemagglutinin (HA) on the cell surface was observed (Figure 1F, yellow regions). Even in permeabilized cells, most of the Cachd1 appeared to be associated with the cell surface (Figure S1B).

We then co-expressed Ca_V_2.2 with a C-terminally GFP-tagged Cachd1 and found that immunoprecipitation (IP) of Cachd1_GFP with GFP Ab was able to coIP Ca_V_2.2. As a control, there was no coIP of Ca_V_2.2 using Cachd1 without a GFP tag (Figure 1G), expression of which was confirmed using Cachd1 Ab (Figure S1C). The interaction of Cachd1 with Ca_V_2.2 was likely to be weaker than that observed for α_2_δ-1 because no coIP of Cachd1 with GFP_Ca_V_2.2 was observed in experiments performed under conditions similar to those shown for α_2_δ-1 in Figure 1C (Figure S1D).

### The D122A Mutation in Ca_V_2.2 Prevents the Effect of α_2_δ-1 but Not Cachd1 on Ca_v_2.2 Currents

**Figure 2:**
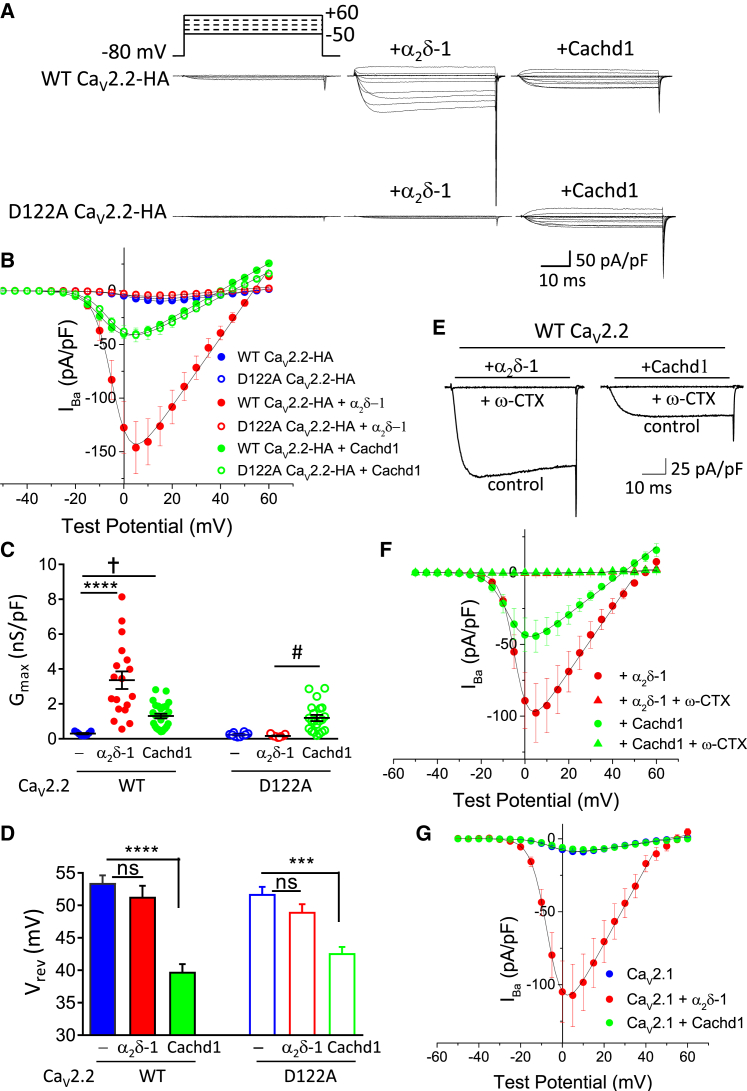
D122A Mutation of Ca_V_2.2 Abolishes Ca_V_2.2 Current Enhancement by α_2_δ-1 but Not Cachd1 (A) Example families of Ca_V_2.2 currents for WT Ca_V_2.2-HA (top row) and D122A Ca_V_2.2-HA (bottom row), co-expressed with β1b and either no α_2_δ (left), α_2_δ-1 (center), or rCachd1 (right). Holding potential −80 mV, steps between −50 and +60 mV for 50 ms (top, applies to all traces). (B) Mean (± SEM) current-voltage relationships for the conditions shown in (A). WT Ca_V_2.2-HA (solid circles; n = 9, 18, and 27 for no α_2_δ, α_2_δ-1, and Cachd1, respectively) and D122A Ca_V_2.2-HA (open circles; n = 10, 11, and 21 for no α_2_δ, α_2_δ-1, and Cachd1, respectively) were co-expressed with β1b and either no α_2_δ (blue), α_2_δ-1 (red), or Cachd1 (green). The individual and mean data were fit with a modified Boltzmann equation (STAR Methods). The potential for half-maximal activation (V_50,act_) (mV) was +4.9 ± 1.3, −2.9 ± 1.6, and −4.6 ± 0.5 for WT Ca_V_2.2-HA with no α_2_δ, α_2_δ-1 and Cachd1, respectively, and +4.5 ± 0.7, +3.7 ± 0.9, and −3.6 ± 0.7 for D122A Ca_V_2.2-HA with no α_2_δ, α_2_δ-1, and Cachd1, respectively. (C) G_max_ (nanosiemens [nS]/picofarad [pF]) from the current-voltage relationships shown in (B). Individual data (same symbols as in B) and mean ± SEM are plotted. †p = 0.0483, #p = 0.0357, ^∗∗∗∗^p < 0.0001 (1-way ANOVA and Sidak’s *post hoc* test correcting for multiple comparisons). (D) Bar charts of mean ± SEM for reversal potential (V_rev_) (millivolt) for the conditions shown in (B). WT Ca_V_2.2-HA (solid bars) and D122A Ca_V_2.2-HA (open bars) were co-expressed with β1b and either no α_2_δ (blue), α_2_δ-1 (red), or Cachd1 (green). ns, not significant; ^∗∗∗^p < 0.001, ^∗∗∗∗^p < 0.0001 (1-way ANOVA and Sidak’s *post hoc* test correcting for multiple comparisons). (E) Examples of current traces at +5 mV of WT Ca_V_2.2 co-expressed with β1b and either α_2_δ-1 (left) or Cachd1 (right) before (control) and after application of 1 μM ω-conotoxin GVIA (+ ω-CTX). (F) Mean (± SEM) current-voltage relationships before the application of ω-conotoxin GVIA for WT Ca_V_2.2-HA co-expressed with β1b and either α_2_δ-1 (red circles, n = 7) or Cachd1 (green circles, n = 6). The application of ω-conotoxin GVIA (1 μM) produced a complete block of WT Ca_V_2.2 co-expressed with α_2_δ-1 (red triangles, n = 7) or Cachd1 (green triangles, n = 6). (G) Mean (± SEM) current-voltage relationships for Ca_V_2.1 co-expressed with β1b and either no α_2_δ (blue solid circles, n = 12), α_2_δ-1 (red solid circles, n = 14), or Cachd1 (green solid circles, n = 12). The individual and mean data were fit with a modified Boltzmann equation (STAR Methods).

In agreement with the coIP results, we found that expression of rCachd1 produced a consistent increase (4.5-fold) in WT Ca_V_2.2 currents (in the additional presence of β1b) despite its disrupted MIDAS motif (Figures 2A–2C).

We then examined the effect of the D122A mutation on the ability of α_2_δ-1 and Cachd1 to increase Ca_V_2.2 currents. We found that, although α_2_δ-1 increased the maximum conductance (G_max_) of WT Ca_V_2.2 by 11.5-fold, it produced no increase in the case of D122A Ca_V_2.2, for which the currents were of the same amplitude as WT Ca_V_2.2 without α_2_δ (Figures 2A–2C). Very similar results to those observed with α_2_δ-1 were obtained for α_2_δ-3 (Figure S2).

By contrast, we found that Cachd1 produced a similar increase (5.2-fold) in G_max_ for D122A Ca_V_2.2 to that observed for WT Ca_V_2.2 (Figures 2A–2C). This result indicates that the effect of Cachd1 is unlikely to be dependent on co-ordination of a divalent cation between its disrupted MIDAS motif and loop I of the α1 subunit and, therefore, might involve other interactions with Cachd1. Like α_2_δ-1, Cachd1 induced a shift of current activation to more hyperpolarized potentials for both WT and D122A Ca_V_2.2, as shown in the current-voltage (I-V) relationships (Figure 2B).

It is noteworthy that, for both WT Ca_V_2.2 and D122A Ca_V_2.2, we observed that the barium current (I_Ba_) in the presence of rCachd1 had an apparent reversal potential that was ∼11.6 mV more negative compared with WT Ca_V_2.2 currents in the presence of α_2_δ-1, suggesting a possible effect of Cachd1 on ion selectivity (Figure 2D). Under the same recording conditions, no effect was observed of rCachd1, expressed alone, on endogenous conductances in tsA-201 cells, which might independently account for this effect on the reversal potential. Furthermore, the ω-conotoxin GVIA (GVIA) completely abolished Ca_V_2.2 currents when coexpressed with β1b and rCachd1, as it did when β1b and α_2_δ-1 (Figures 2E and 2F). Note that the negative shift in reversal potential induced by Cachd1, relative to α_2_δ-1, remains present in this dataset prior to ω-conotoxin GVIA application (Figure 2F).

Surprisingly, rCachd1 did not increase currents through the related Ca_V_2.1 channel under the same conditions, although α_2_δ-1 produced the expected effect (Figure 2G; Figures S3A and S3B), indicating that there is selectivity in the effect of Cachd1 for specific calcium channel isoforms.

### The D122A Mutation in Ca_V_2.2 Reduces the Effect of α_2_δ but Not Cachd1 on Ca_V_2.2 at the Plasma Membrane

**Figure 3:**
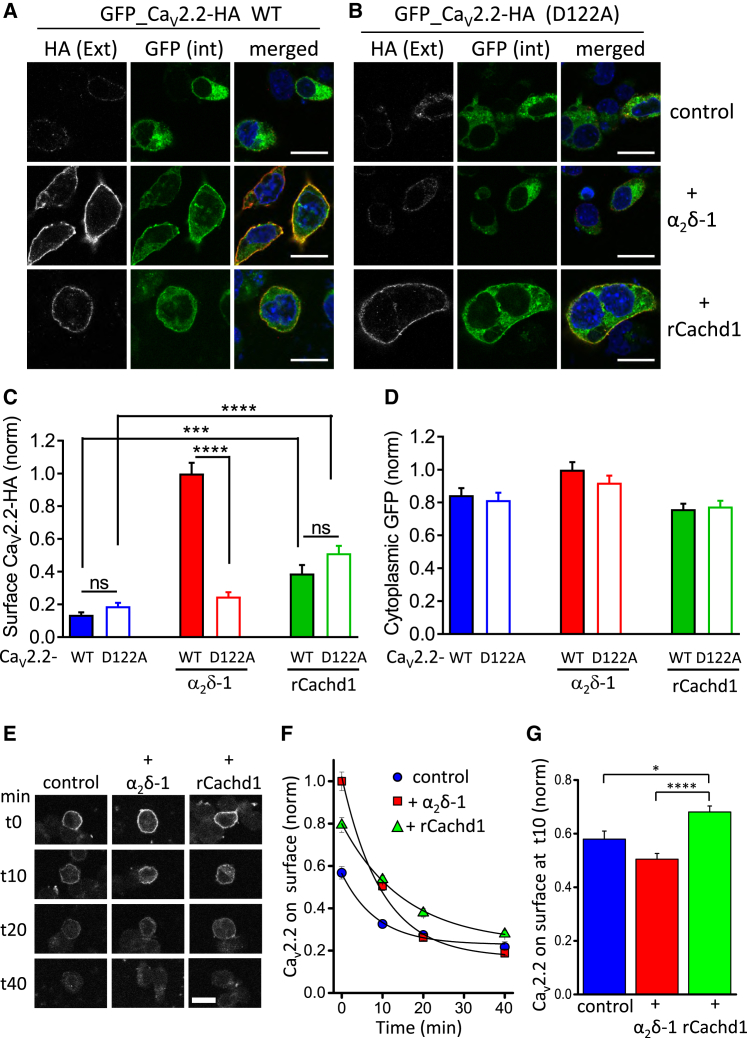
D122A Mutation in Ca_V_2.2 Prevents Effect of α_2_δ-1 but Not Cachd1 on Ca_V_2.2 Cell Surface Expression in N2A Cells (A and B) Representative confocal images of N2A cells expressing GFP_Ca_V_2.2-HA WT (A) or D122A (B) with β1b in the absence of α_2_δ (control, top row) with α_2_δ-1 (center row) or rCachd1 (bottom row). Intact cells (non-permeabilized) were incubated with rat anti-HA Ab for 1 hr to visualize extracellular HA staining on the plasma membrane (left, white) to be compared with intracellular GFP fluorescence (center). Merged images (with HA in red) are shown on the right; DAPI was used to stain the nuclei (blue). Scale bars, 20 μm. (C and D) Bar charts showing cell surface expression of WT (closed bars) and D122A Ca_V_2.2-HA (open bars), determined by HA staining prior to permeabilization (C), and cytoplasmic expression determined by GFP fluorescence (D). Blue bars are for the control condition without α_2_δ or Cachd1, red bars are with α_2_δ-1, and green bars are with rCachd1. Data (mean ± SEM) for 164 (WT − α_2_δ-1), 220 (WT + α_2_δ-1), 185 (WT + Cachd1), 165 (D122A − α_2_δ-1), 203 (D122A + α_2_δ-1), and 232 (D122A + rCachd1) cells from 3 experiments were normalized to the WT Ca_V_2.2-HA + α_2_δ-1 condition in each experiment. ^∗∗∗^p < 0.001, ^∗∗∗∗^p < 0.0001 (one-way ANOVA and Sidak’s *post hoc* test correcting for multiple comparisons). (E) Representative confocal images of N2A cells expressing Ca_V_2.2-bungarotoxin binding site (BBS) and labeled with BTX-488. Cells were co-transfected with β1b and either empty vector (control, left), α_2_δ-1 (center), or rCachd1 (right). Cells were incubated at 17°C with BTX-488 for 30 min and then imaged at different time points, from zero (t0) to 40 min (t40). Scale bar, 20 μm. (F) Time course of endocytosis of cell surface Ca_V_2.2-BBS in control + β1b alone (blue circles), + α_2_δ-1 (red squares), and + rCachd1 (green triangles). BTX-488 fluorescence was normalized to the mean fluorescence of the + α_2_δ-1 condition at t0. The results are shown as the mean ± SEM. The number of cells (n) obtained from 5 independent experiments varies from 349 to 789 for each time point and condition. The data were fitted with single exponentials. The time constants of the fits were 8.5 min, 9.9 min, and 15.4 min for control, + α_2_δ-1, and + rCachd1, respectively. (G) Bar chart (mean ± SEM) comparing the reduction of cell surface Ca_V_2.2-BBS at 10 min for the 3 conditions. BTX-488 fluorescence was normalized to t0 for each condition. BTX-488 fluorescence was reduced by 42% ± 3% for control (blue bar, n = 743 cells), 50% ± 2% for + α_2_δ-1 (red bar, n = 646 cells), and 32% ± 2% for + rCachd1 (green bar, n = 784 cells). ^∗^p = 0.0109, ^∗∗∗∗^p < 0.0001 (one-way ANOVA and Bonferroni’s *post hoc* test for multiple comparisons).

We then compared the cell surface expression of WT and D122A Ca_V_2.2-HA, either in the presence or absence of α_2_δ-1 or Cachd1, using N2A cells. All conditions included the β subunit β1b, and Ca_V_2.2-HA was N-terminally GFP-tagged to identify all transfected cells. WT Ca_V_2.2-HA was well expressed at the cell surface when co-expressed with α_2_δ-1, which resulted in a 7.3-fold increase compared with its cell surface expression in the absence of α_2_δ (Figures 3A and 3C). In contrast, D122A Ca_V_2.2-HA exhibited a very low expression level at the plasma membrane, which was similar in the presence and absence of α_2_δ-1 (Figures 3B and 3C). In contrast, intracellular expression of WT or D122A Ca_V_2.2-HA (Figures 3A and 3B) was not significantly different with and without α_2_δ-1 (Figure 3D).

In the same experiment, we also investigated the effect of rCachd1 on cell surface expression of Ca_V_2.2-HA. We found that it produced an increase of 2.9-fold in cell surface expression of WT Ca_V_2.2-HA (Figures 3A and 3C). Very similar results were obtained for zCachd1 in tsA-201 cells (Figures S4A and S4B). Of great interest, and similar to its effect on calcium currents, is that rCachd1 increased cell surface expression of D122A Ca_V_2.2-HA by an extent similar to its effect on WT Ca_V_2.2-HA (a 2.7-fold increase compared with D122A Ca_V_2.2-HA alone; Figures 3B and 3C). Intracellular expression of WT or D122A Ca_V_2.2-HA (Figures 3A and 3B) was not significantly different with or without rCachd1 (Figure 3D).

To understand the mechanism of action of Cachd1, we compared the endocytosis rates of Ca_V_2.2 in the presence of β1b and either without α_2_δ or plus either α_2_δ-1 or Cachd1 (Figures 3E–3G), using a method described previously (Cassidy et al., 2014). We found that Cachd1 reduced the endocytosis rate of Ca_V_2.2 (Figures 3E–3G). The mean endocytosis time constant was increased from 8.5 min for Ca_V_2.2 + β1b to 15.4 min in the additional presence of Cachd1 (Figure 3F). This is unlike α_2_δ-1, which has no effect on Ca_V_2.2 endocytosis (Cassidy et al., 2014), a result confirmed here. This effect of Cachd1 on endocytosis may therefore contribute to the increased cell surface expression of Ca_V_2.2.

### The D122A Mutation Abolishes the Effect of α_2_δ-1 on the Trafficking of Ca_V_2.2 into Cultured Hippocampal Neurites

**Figure 4:**
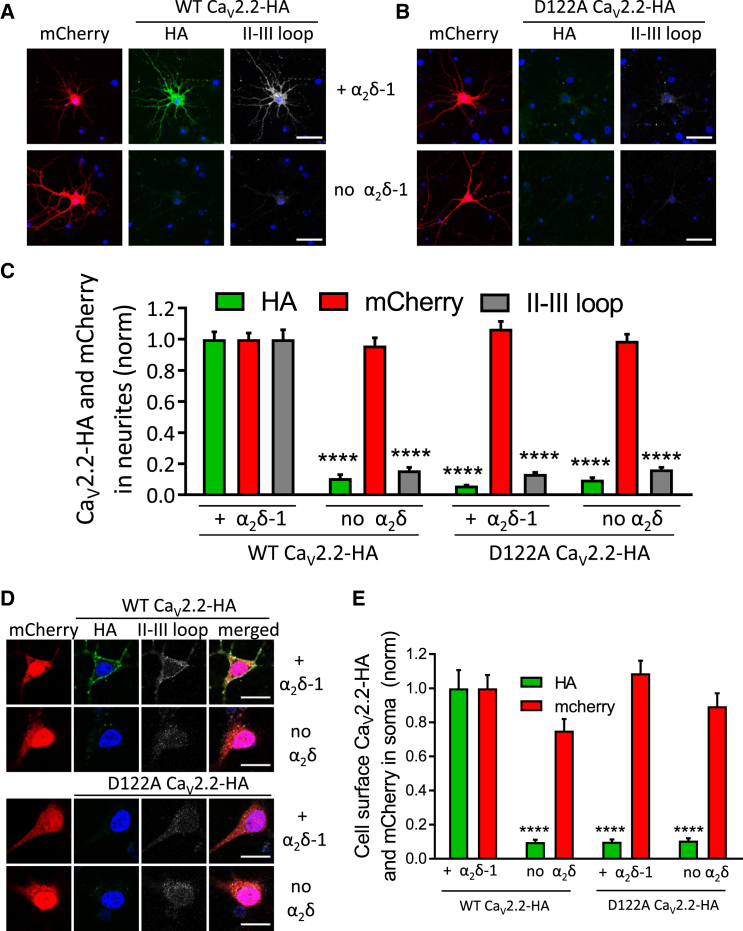
D122A Mutation in Ca_V_2.2 Prevents the Effect of α_2_δ-1 on Ca_V_2.2-HA Cell Surface Expression in Hippocampal Neurites and Somata (A) Representative confocal images showing neurites of hippocampal neurons expressing Ca_V_2.2-HA WT together with β1b and mCherry in the presence (top row) or absence (bottom row) of α_2_δ-1. Expression of mCherry is shown in red (left). Ca_v_2.2-HA (green, center) was stained using rat anti-HA Ab prior to permeabilization, and the rabbit II-III loop Ab (white, right) after permeabilization. DAPI was used to visualize the nucleus (blue). Scale bars, 50 μm. (B) As for (A) but for hippocampal neurons co-expressing Ca_v_2.2-HA D122A with β1b and mCherry in the presence (top row) or absence (bottom row) of α_2_δ-1. (C) Bar chart (mean ± SEM) showing expression of WT and D122A Ca_V_2.2-HA, determined by both HA staining prior to permeabilization (green bars) and II-III loop staining after permeabilization (gray bars), together with the expression marker mCherry. Data for 197 (WT + α_2_δ-1), 130 (WT − α_2_δ-1), 174 (D122A + α_2_δ-1), and 211 (D122A − α_2_δ-1) neurites from 4 separate transfections in 2 experiments were normalized to the WT Ca_V_2.2-HA + α_2_δ-1 condition in each experiment. ^∗∗∗∗^p < 0.0001 (1-way ANOVA compared with WT Ca_V_2.2 + α_2_δ-1, with Sidak’s *post hoc* analysis correcting for multiple comparisons). (D) Representative confocal images showing hippocampal somata expressing Ca_V_2.2-HA WT (top two rows) or Ca_v_2.2-HA D122A (bottom two rows) together with β1b and mCherry in the presence (top row) or absence (bottom row, control) of α_2_δ-1. Expression of mCherry is shown in red (first panel). Ca_v_2.2-HA (green, second panel) was stained using rat anti-HA Ab in non-permeabilized cells, and the rabbit II-III loop Ab (white, third panel) after permeabilization. DAPI was used to visualize the nucleus (blue), and the merged image is shown in the fourth panel. Scale bars, 20 μm. (E) Bar chart (mean ± SEM) showing expression of WT and D122A Ca_V_2.2-HA, determined by HA staining prior to permeabilization (green bars), together with expression marker mCherry (red bars). Data for 32 (WT + α_2_δ-1), 26 (WT - α_2_δ-1), 37 (D122A + α_2_δ-1), and 35 (D122A − α_2_δ-1) cell bodies from 4 separate transfections in 2 experiments were normalized to the WT Ca_V_2.2-HA + α_2_δ-1 condition in each experiment. ^∗∗∗∗^p < 0.0001 (1-way ANOVA compared with WT Ca_V_2.2 + α_2_δ-1, with Sidak’s *post hoc* analysis correcting for multiple comparisons).

Because we have found the presence of α_2_δ to be a key regulator of trafficking of Ca_V_2.2 into neuronal processes (Kadurin et al., 2016), we investigated whether the D122A mutation would influence this. Cultured hippocampal neurons were transfected after 7 days in culture, by which time there was already extensive neurite outgrowth. All conditions included β1b and mCherry as a control for successful transfection. After ∼7 days of expression, as expected, WT Ca_V_2.2-HA was strongly trafficked into hippocampal neuronal processes when co-expressed with α_2_δ-1 (Figures 4A and S4C). In contrast, there was almost no trafficking of D122A Ca_v_2.2-HA into hippocampal neurites when co-expressed with α_2_δ-1 (Figures 4B and 4C). Its level in the neurites was only 6% of that of WT Ca_V_2.2-HA with α_2_δ-1 (Figure 4C). Similarly, staining the intracellular pool of Ca_V_2.2 using the II-III linker Ab showed that the level of D122A Ca_V_2.2-HA was only 13% of WT Ca_V_2.2-HA (Figure 4C), further indicating that the effect of α_2_δ-1 is on trafficking Ca_V_2.2 into the neurites.

In the absence of α_2_δ-1, there was almost no trafficking of WT Ca_V_2.2-HA into hippocampal neurites (Figures 4A and 4C). The same was true for D122A Ca_V_2.2-HA, its level being similar in the presence and absence of α_2_δ-1 (Figures 4B and 4C).

We also analyzed cell surface expression of Ca_V_2.2 in the cell bodies of these hippocampal neurons and found essentially the same result; the increase in cell surface expression resulting from α_2_δ-1 was abrogated by the D122A mutation (Figures 4D and 4E), although an intracellular signal was present for both WT and D122A Ca_V_2.2-HA (Figure 4D).

### Cachd1 Increases the Trafficking of Ca_V_2.2 into Hippocampal Neurites

**Figure 5:**
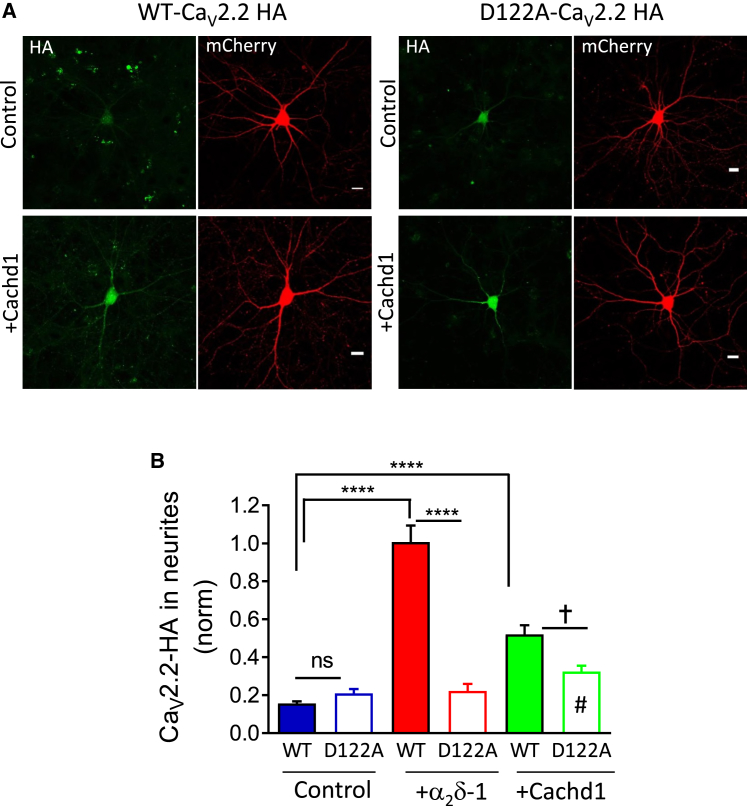
Cachd1 Promotes Ca_V_2.2-HA Distribution in Hippocampal Neurites (A) Representative confocal images showing neurites of hippocampal neurons expressing Ca_V_2.2-HA WT (left) or Ca_v_2.2-HA D122A (right) together with β1b and mCherry in the absence (top row) or presence (bottom row) of Cachd1. Expression of mCherry is shown in red. Scale bars, 20 μm. (B) Bar chart (mean ± SEM) showing neurite expression of WT and D122A Ca_V_2.2-HA, determined by HA staining of intact cells prior to permeabilization. Shown are data for 137 (WT, blue solid), 144 (D122A, blue open), 200 (WT + α_2_δ-1, red solid), 111 (D122A + α_2_δ-1, red open), 175 (WT + Cachd1, green solid), and 152 (D122A + Cachd1, green open) neurites from 3 experiments. Data were normalized to the WT Ca_V_2.2-HA + α_2_δ-1 condition in each experiment. ^∗∗∗∗^p < 0.0001, †p = 0.0473 between WT Ca_V_2.2-HA + Cachd1 and D122A Ca_V_2.2-HA + Cachd1, #p = 0.5563 between D122A Ca_V_2.2-HA and D122A Ca_V_2.2-HA + Cachd1 (1-way ANOVA and Sidak’s *post hoc* analysis correcting for multiple comparisons).

We therefore also investigated the effect of Cachd1 on trafficking of Ca_V_2.2-HA into hippocampal neurites (Figure 5A). We found that it produced a consistent increase of WT Ca_V_2.2-HA by 3.3-fold (Figures 5A and 5B), although this was less than the 6.8-fold increase produced by α_2_δ-1 in the same experiment. However, in this experimental context, Cachd1 was much less able to traffic D122A Ca_V_2.2-HA into neurites than WT Ca_V_2.2-HA (Figures 5A and 5B), unlike the result observed in the N2A cell line. This result is in agreement with our previous finding that trafficking of Ca_V_2.2 is more stringently controlled in neurons than in cell lines (Kadurin et al., 2016).

### Cachd1 Competes with α_2_δ-1 for Interaction with Ca_V_2.2

**Figure 6:**
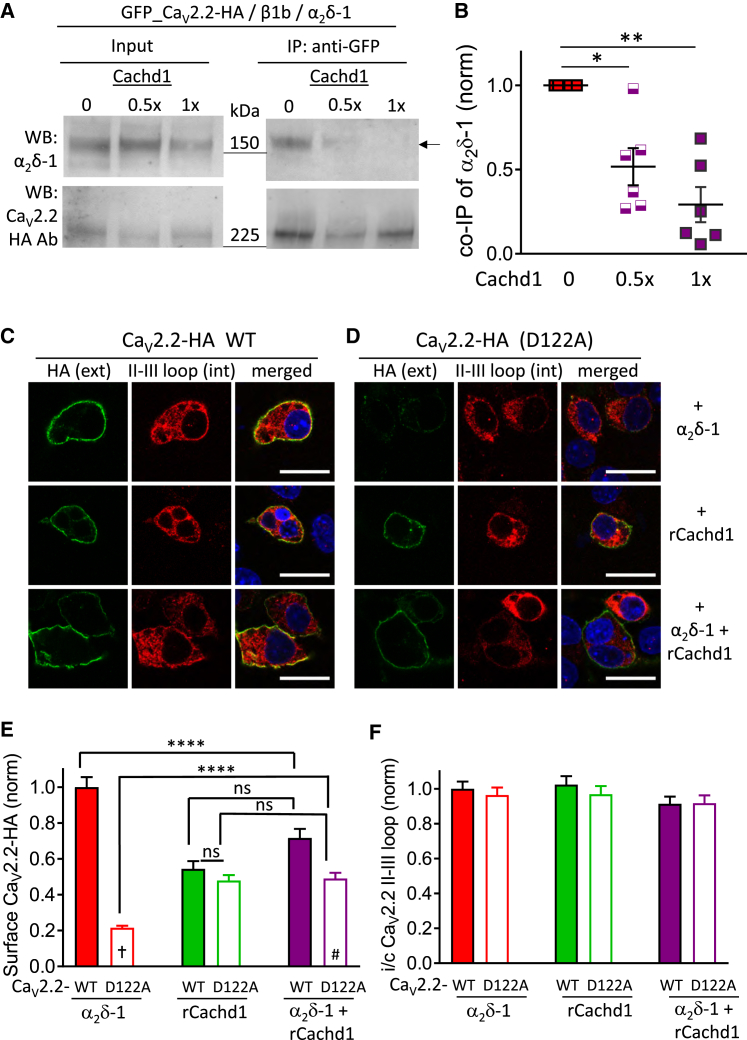
Cachd1 Competes with α_2_δ-1 for Interaction with Ca_V_2.2 (A) IP of GFP_Ca_V_2.2-HA and coIP of α_2_δ-1 in the absence of Cachd1 (left lane, 0; includes TASK3 cDNA as a control) or presence of 0.5× or 1× Cachd1 (center and right lanes, with 0.5× or no TASK3 cDNA, respectively). See STAR Methods for details regarding the cDNA mixes. Shown are WCL input (left) and IP (right) for α_2_δ-1 (top) and GFP_Ca_V_2.2-HA (bottom, detected with HA Ab). The IP was performed with GFP Ab. Co-IP of α_2_δ-1 is shown at the top right (arrow). (B) Scatterplot showing the effect of Cachd1 on α_2_δ-1 co-IP with GFP_Ca_V_2.2-HA for 6 experiments, including that in (A). Shown are no Cachd1 (red squares), 0.5× Cachd1 (purple half-closed squares), and 1× Cachd1 (purple squares). Data are the ratio of α_2_δ-1 in IP/input, normalized to no Cachd1 in each experiment. Mean and SEM are also shown; ^∗^p = 0.0144, ^∗∗^p = 0.0021 (1-way ANOVA with repeated measures and Sidak’s *post hoc* test with multiple comparisons correction, comparing +Cachd1 to no Cachd1). (C and D) Representative confocal images of N2A cells expressing Ca_V_2.2-HA WT (C) or D122A (D) with β1b in the presence of α_2_δ-1 (top row), rCachd1 (center row), or both α_2_δ-1 and rCachd1 (bottom row). Intact cells (non-permeabilized) were incubated with rat anti-HA Ab for 1 hr to show extracellular HA staining on the plasma membrane (left, green). The cells were then permeabilized and stained with the Ca_V_2.2 II-III loop Ab (center, red). Merged images are shown on the right; DAPI was used to stain the nuclei (blue). Scale bars, 20 μm. (E and F) Bar charts showing cell surface expression of WT (closed bars) and D122A Ca_V_2.2-HA (open bars), determined by HA staining prior to permeabilization (E), and cytoplasmic expression determined by II-III loop staining after permeabilization (F). Ca_V_2.2 with α_2_δ-1 is shown in red, with rCachd1 in green, and with both α_2_δ-1 and rCachd1 in purple. Data for 328 (WT + α_2_δ-1), 255 (WT + rCachd1), 231 (WT + both), 272 (D122A + α_2_δ-1), 270 (D122A + rCachd1), and 225 (D122A + both) cells from 3 experiments were normalized to the WT Ca_V_2.2-HA + α_2_δ-1 condition in each experiment. ^∗∗∗∗^p < 0.0001, †p < 0.0001 versus WT, #p = 0.005 versus WT (1-way ANOVA with Sidak’s *post hoc* test, comparing all columns and correcting for multiple comparisons).

The preceding experiments indicate that Cachd1 does not utilize the domain I D122 interaction site on Ca_V_2.2, which is required by α_2_δ-1 for interaction via its MIDAS motif. Because Cachd1 still has a VWA domain, albeit with a disrupted MIDAS motif, we wondered whether Cachd1 might potentially be an antagonist at this site, interfering with the effect of α_2_δ-1. We found that Cachd1 concentration-dependently reduced the coIP of α_2_δ-1 with GFP_Ca_V_2.2, by 71% when both cDNAs were transfected in equal amounts (Figures 6A and 6B), indicating that Cachd1 can obstruct the interaction site on Ca_V_2.2 utilized by α_2_δ-1.

Furthermore, in experiments measuring Ca_V_2.2 cell surface expression (Figures 6C and 6E), the additional presence of Cachd1 significantly reduced the effect of α_2_δ-1 on cell surface expression of Ca_V_2.2-HA by 28.4% (Figure 6E, purple bar) but had no effect on its intracellular expression (Figure 6F). In this experiment, the increase in cell surface expression of Ca_V_2.2-HA in the presence of Cachd1 alone was 54.4% of the Ca_V_2.2-HA + α_2_δ-1 level (Figure 6E, green bar), and this increase with Cachd1 was still observed for D122A Ca_V_2.2 (47.9% of the Ca_V_2.2 + α_2_δ-1 level; Figure 6E, open green bar). The additional presence of α_2_δ-1 had no effect on the increase of D122A Ca_V_2.2 cell surface expression in the presence of Cachd1 (Figure 6E, open purple bar).

**Figure 7:**
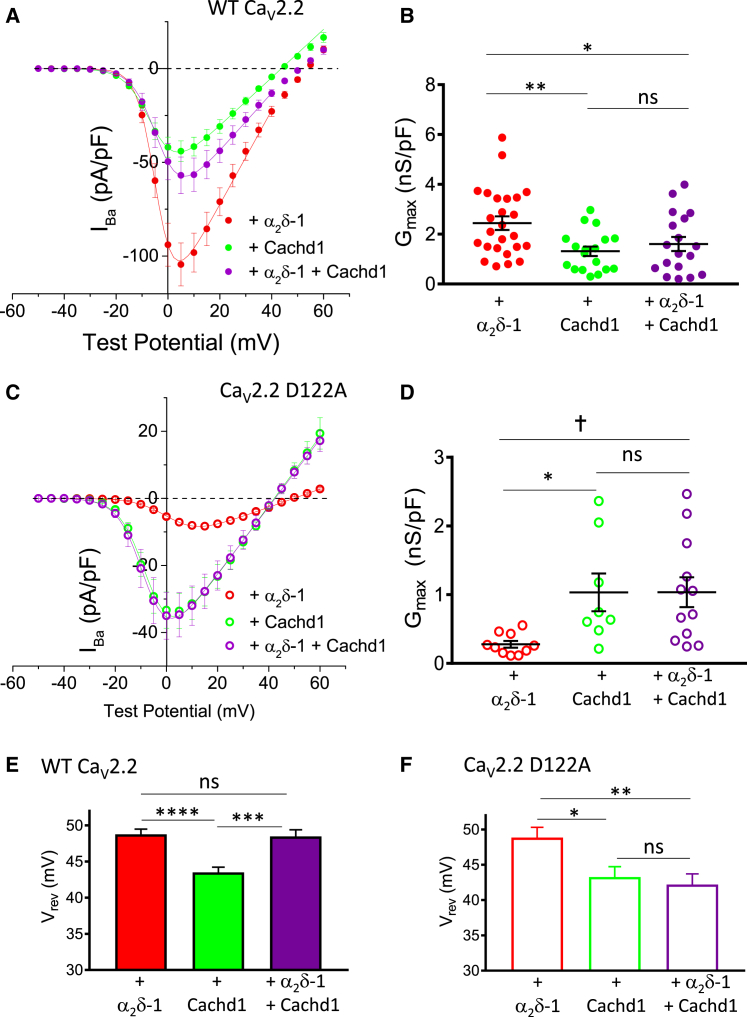
Cachd1 Competes with α_2_δ-1 for Effects on Ca_V_2.2 Currents (A) Mean (± SEM) current-voltage relationships for WT Ca_V_2.2-HA co-expressed with β1b and either α_2_δ-1 (red solid circles, n = 25), Cachd1 (green solid circles, n = 18), or α_2_δ-1 and Cachd1 (purple solid circles, n = 18). The individual and mean data were fit with a modified Boltzmann equation (STAR Methods). (B) G_max_ (nS/pF) from the current-voltage relationships shown in (A). Individual data (same symbols as in A) and mean ± SEM are plotted. ^∗^p = 0.0483, ^∗∗^p = 0.0086 (1-way ANOVA with Holm-Sidak’s *post hoc* test correcting for multiple comparisons). (C) Mean (± SEM) current-voltage relationships for D122A Ca_V_2.2-HA co-expressed with β1b and either α_2_δ-1 (red open circles, n = 10), Cachd1 (green open circles, n = 8), or α_2_δ-1 and Cachd1 (purple open circles, n = 12). (D) G_max_ (nS/pF) from the current-voltage relationships shown in (C). Individual data (same symbols as in C) and mean ± SEM are plotted. ^∗^p = 0.0342, †p = 0.0265 (1-way ANOVA with Holm-Sidak’s *post hoc* test correcting for multiple comparisons). (E and F) Bar charts of mean (± SEM) V_rev_ (millivolt) for the conditions shown in (A) and (C), respectively. ^∗^p = 0.0396, ^∗∗^p = 0.0088, ^∗∗∗^p = 0.0002, ^∗∗∗∗^p < 0.0001 (1-way ANOVA with Holm-Sidak’s *post hoc* test correcting for multiple comparisons).

In a direct parallel with these results, we observed that Cachd1 co-expression significantly reduced Ca_V_2.2 currents in the presence of α2δ-1, almost to the level of Ca_V_2.2 currents in the presence of Cachd1 alone (Figures 7A and 7B), but α_2_δ-1 had no effect on the ability of Cachd1 to increase D122A Ca_V_2.2 currents (Figures 7C and 7D). Interestingly, when α_2_δ-1 and Cachd1 were co-expressed, the reversal potential for WT Ca_V_2.2 currents was identical to that observed with α_2_δ-1 alone (Figure 7E), pointing to preferential α_2_δ-1 interaction on the cell surface. By contrast, for D122A Ca_V_2.2 currents, the reversal potential in the presence of both α_2_δ-1 and Cachd1 was similar to that for Cachd1 alone (Figure 7F), reinforcing the evidence for a lack of interaction of this mutant with α_2_δ-1.

## Discussion

In this study, we uncovered a mechanism for influencing Ca_V_2.2 channel trafficking and function mediated by the α_2_δ-like protein Cachd1, despite its VWA domain having a disrupted MIDAS motif.

We first established the importance of interaction of Ca_V_2.2 with the α_2_δ-1 VWA domain for its cell surface expression and function by mutating the predicted α_2_δ interaction site in Ca_V_2.2 (D122), which is in the first extracellular loop of domain I. This mutation completely abolished the ability of α_2_δ-1 to increase the trafficking of Ca_V_2.2 and to increase Ca_V_2.2 currents, indicating that it is the main interaction site between the channel and α_2_δ. This was confirmed by our coIP results.

Surprisingly, Cachd1 consistently produced a 4.5-fold increase in Ca_V_2.2 currents and also increased the cell surface expression of Ca_V_2.2 by 2.9-fold. However, in contrast to α_2_δ subunits, neither the trafficking effects of Cachd1 in N2A cells nor its effect on calcium channel currents were affected by the presence of the D122A mutation in Ca_V_2.2. Therefore, these effects of Cachd1 are likely not to be mediated via its disrupted MIDAS motif but, rather, due to interactions of the Cache or other domains in the protein. Interestingly, our results indicate that the effect of Cachd1 on cell surface expression of Ca_V_2.2 involves a reduction in Ca_V_2.2 endocytosis. It is highly unlikely that this is a non-specific effect because we have previously provided many examples of protein constructs that do not increase Ca_V_2.2 currents or cell surface expression (Ferron et al., 2008, Kadurin et al., 2016, Kadurin et al., 2017, Macabuag and Dolphin, 2015).

In α_2_δ-1 and α_2_δ-2, the key MIDAS motif in the VWA domain contains three polar or negatively charged residues and has the sequence DVSGS. It is these three residues (D259, S261, and S263 in rat α_2_δ-1 used here), plus two others (T331 and D363) in separate loops of the VWA domain that, together with the VWA protein ligand (Ca_V_2.2 in this study), coordinate a divalent cation. In α_2_δ-3 and α_2_δ-4, one of these other coordinating residues is non-polar, but the MIDAS motif is intact. We confirm here that the increase in Ca_V_2.2 currents caused by α_2_δ-3 is also abolished by the D122A mutation in Ca_V_2.2. A similar result was found for the interaction of Ca_V_1.2 with α_2_δ-1 in an extensive site-directed mutagenesis study (Bourdin et al., 2017).

By contrast, Cachd1 contains a VWA domain that has a disrupted MIDAS motif (DHGAS), a sequence that is conserved in the human, rat, mouse, and zebrafish Cachd1 proteins. This conservation across species supports the possibility that it may retain some function. Indeed, the ability of Cachd1 to increase the trafficking of Ca_V_2.2 into hippocampal neurites was significantly reduced for D122A Ca_V_2.2, suggesting that the disrupted MIDAS motif in Cachd1 may play some role in the interaction required for Ca_V_2.2 trafficking into neurites. This result also indicates that there may be more stringent trafficking requirements for this channel in neurons. We drew a similar conclusion in a previous study, in which we showed that immature pro-α_2_δ-1 could traffic Ca_V_2.2 to the cell surface in non-neuronal cells but not into hippocampal neurites, where mature proteolytically processed α_2_δ-1 was required (Kadurin et al., 2016).

We have shown previously that, when the three polar or charged residues of the α_2_δ-1 MIDAS motif are mutated to alanine, α_2_δ-1 still associates with Ca_V_2.2, as judged by its ability to occlude antigenic epitopes within the Cache domains of α_2_δ-1 (Cassidy et al., 2014), although it fails to promote Ca_V_2.2 trafficking. This indicates that there are certainly additional interaction sites as well as the MIDAS site interaction between α_2_δ-1 and Ca_V_2.2. The cryo-EM structure of the skeletal muscle calcium channel complex indicates clearly that α_2_δ-1 interacts with Ca_V_1.1 via multiple sites in addition to the divalent cation-mediated VWA domain interaction, including an interaction of a Cache domain with the turret of pore loop 5 in domain III (Wu et al., 2016). Such an interaction with the pore domain of Ca_V_2.2 could also potentially explain the effect of Cachd1 on the apparent reversal potential. Because Cachd1 was able to coIP Ca_V_2.2, partially co-localized with Ca_V_2.2 on the cell surface of transfected cells, and also affected the reversal potential of these channels, it is clear that Cachd1 is not solely a trafficking protein but influences functional channels in the plasma membrane. The influence of another protein on the reversal potential of a channel, interpreted as an effect on its selectivity filter, has been observed previously (Stephan et al., 2018). The lack of effect of Cachd1 on Ca_V_2.1 currents may relate to a particular splice variant or be common to all isoforms of Ca_V_2.1 and should allow us to localize the site of selective interaction of Cachd1 with Ca_V_2.2 in the future.

The finding that Cachd1 was able to inhibit the co-IP between Ca_V_2.2 and α_2_δ-1 and reduce the effect of α_2_δ-1 on Ca_V_2.2 cell surface expression and Ca_V_2.2 currents indicates that, *in vivo*, it could play either a positive or an inhibitory role on Ca_V_2.2 currents, depending on the degree of association of the Ca_V_2.2 channels with α_2_δ. From mRNA expression screens, Cachd1 is widely expressed in many tissues, including the brain, lungs, and small intestine. Of particular interest here is that Cachd1 mRNA expression was highest in dorsal root ganglia of all mouse tissues examined (see the transcriptome database described in Ray et al., 2018), raising the intriguing possibility that Cachd1 may modulate the efficacy of α_2_δ-1 following neuropathic injury, a hypothesis that we will investigate in future studies.

Within the brain, there is strong expression of α_2_δ-1 mRNA in the mouse hippocampus (Schlick et al., 2010). It is expressed strongly in CA1 and also present in dentate granule neurons. Cachd1 mRNA is also expressed in the mouse hippocampus, and, within the pyramidal cell layer, it is particularly prominent in CA3 but also in CA1 (Allen Mouse Brain Atlas; mouse.brain-map.org/api/index.html). Thus, Cachd1 and α_2_δ-1 are likely to be expressed in overlapping cell types in the hippocampus. Within the rat hippocampus, there is a robust signal for α_2_δ-1 protein in synaptic regions, including the dentate gyrus molecular layer, the *stratum lucidum* of CA3, and the CA1 *stratum oriens* and *stratum radiatum* (Nieto-Rostro et al., 2014, Taylor and Garrido, 2008); however, there are no equivalent data available for Cachd1 because of the paucity of antibodies and lack of knockout control tissue. Furthermore, in a large-scale proteomic study of non-neuronal cell lines, several proteins interacting with Cachd1 have been described recently (Huttlin et al., 2017); this suggests other potential roles for this protein in non-excitable cells (Rutledge et al., 2017).

In the future, it will be of great interest to determine the effect of Cachd1 on native calcium channels and whether its expression is altered in conditions such as neuropathic injury of primary afferent neurons to further elucidate its physiological role and to understand whether it competes endogenously with α_2_δ-1 or other α_2_δ subunits.

## STAR★Methods

### Key Resources Table

**Table:** 

REAGENT or RESOURCE	SOURCE	IDENTIFIER
**Antibodies**		

α_2_δ-1 Ab	Sigma-Aldrich	Cat # C5105; RRID:AB_258885
Anti-Ca_V_2.2 II-III loop Ab (rabbit polyclonal)	(Raghib et al., 2001)	n/a
Anti-HA Ab rat monoclonal	Sigma-Aldrich	Cat# 11815016001; RRID:AB_390914
Anti-HA Ab rabbit	Sigma-Aldrich	Cat # H6908; RRID:AB_260070
Anti-GAPDH Ab	Ambion	Cat # AM4300; RRID:AB_2536381
Anti-GFP Ab (Living Colors, rabbit polyclonal)	Takara Bio Clontech	Cat # 632375
Anti-rabbit Alexa fluor 594	Thermo Fisher	Cat # R37117; RRID:AB_2556545
Anti-rat Alexa fluor 488	Thermo Fisher	Cat # A-11006; RRID:AB_2534074
Anti-mouse Alexa fluor 647	Thermo Fisher	Cat # A32728; RRID:AB_2633277
Anti-rat fluorescein isothiocyanate	Sigma-Aldrich	Cat # F1763; RRID:AB_259443
Goat anti-rabbit HRP	Biorad	Cat # 1706515; RRID:AB_11125142
Goat anti-rat HRP	Biorad	Cat # 5204-2504; RRID:AB_619913
Goat anti-mouse HRP	Biorad,	Cat # 1721011; RRID:AB_11125936
Affinity-purified Cachd1 rabbit polyclonal Ab	G. T. Powell and S.W Wilson, UCL.	n/a

**Chemicals, Peptides, and Recombinant Proteins**		

ω-conotoxin GVIA	Alomone	Cat # C-300
Penicillin-Streptomycin (10,000 U/mL)	Invitrogen	Cat # 15140-122
Poly-L-lysine	Sigma-Aldrich	Cat # P.6282
Dulbecco’s modified Eagle’s medium	Thermo Fisher	Cat #4 1965-039
GlutaMAX	Invitrogen	Cat # 35050-038
Fugene	Promega	Cat # E2311
Polyjet	Tebu-bio Ltd	Cat # 189-SL100688-1
Opti-MEM	Thermo Fisher	Cat # 41965-039
Neurobasal Medium	Invitrogen	Cat # 10888-022
B27	Thermo Fisher	Cat # 17504044
HEPES	Sigma-Aldrich	Cat # H3375
Horse serum	Invitrogen	Cat # 26050-088
Lipofectamine 2000	Invitrogen	Cat # L3000-008
Premium Grade EZ-link Sulfo-NHS-LC-Biotin	Thermo Fisher	Cat # 21335
Glycine	Sigma-Aldrich	Cat # G8898
SDS	VWR	Cat # 444062F
Protease Inhibitors	Roche	Cat # 11697498001
DTT	Melford	Cat # MB1015
SDS-polyacrylamide gel electrophoresis	Invitrogen	Cat # EA0375BOX
polyvinylidene fluoride (PVDF) membrane	Biorad	Cat # 1620177
streptavidin-agarose beads	Thermo Fisher	Cat # 20347
Igepal	Sigma-Aldrich	Cat # I3021
PNGase-F	Roche Applied Science	Cat # 11365177001
Digitonin	Millipore	Cat # 300410
A/G PLUS Agarose slurry Santa Cruz	Santa Cruz	Cat # Sc-2003
Paraformaldehyde	Sigma-Aldrich	Cat # P6148
Goat serum	Invitrogen	Cat # 6210-072
Triton X-100	Thermo Fisher	Cat # 28314
4’,6-diamidine-2′-phenylindole dihydrochloride (DAPI)	Molecular probes	Cat # nl5995050
VectaShield	Vector Laboratories	Cat # H1000
papain	Sigma-Aldrich	Cat # P4762
L-cysteine	Sigma-Aldrich	Cat # c7755
bovine serum albumin	First Link UK ltd	Cat # 41-00-410
DNase	Sigma-Aldrich	Cat # D5025
Hank’s basal salt solution	Thermo Fisher	Cat # 14175-053
α-bungarotoxin Alexa Fluor® 488 conjugate (BTX488)	Thermo Fisher	Cat # B13422
fetal bovine serum	Invitrogen	Cat # 10270

**Critical Commercial Assays**		

Bradford Assay	Biorad	Cat # 500-0006
ECL 2	Thermo Fisher	Cat # 32132

**Experimental Models: Cell Lines**		

tsA-201 cells	ECACC	Cat # 96121229
Neuro2A cells	ATCC	CCL-131

**Experimental Models: Organisms/Strains**		

Rat Sprague Dawley male	UCL bred in house	n/a

**Oligonucleotides**		

Primer for introducing the D122A mutation. reverse 5′-GACATAGGCGTCTTGGCCCCGTCAG-3′	this paper	n/a
Primer for introducing the D122A mutation forward 5′-CTGACGGGGCCAAGACGCCTATGTC-3′	this paper	n/a

**Recombinant DNA**		

Rabbit Ca_V_2.2 HA	(Cassidy et al., 2014)	n/a
Rat β1b (X61394)	(Pragnell et al., 1991)	n/a
Rat α_2_δ-1 (M86621)	(Kim et al., 1993)	n/a
HA tagged α_2_δ-1	(Kadurin et al., 2012)	n/a
Rat Ca_V_2.1	(Brodbeck et al., 2002)	n/a
Human TASK3 (KCNK9) (NM_001282534)	obtained from Prof. A Mathie	n/a
Zebrafish zCachd1	G. T. Powell and S.W Wilson, UCL.	n/a
Rat rCachd1	OriGene	Cat # RN217577
GFP_Ca_V_2.2-HA	(Macabuag and Dolphin, 2015)	n/a
Ca_V_2.2-BBS	(Cassidy et al., 2014)	n/a
Ca_V_2.2-HA D122A	This paper	n/a
GFP_Ca_V_2.2-HA D122A	This paper	n/a
rCachd1_GFP	This paper	n/a
zCachd1_GFP	G. T. Powell and S.W Wilson, UCL.	n/a
Mouse α_2_δ-3 (AJ010949)	(Klugbauer et al., 1999)	n/a
mcherry (AY678264)	(Shaner et al., 2004)	n/a
CD8	(Shy et al., 2014)	n/a

**Software and Algorithms**		

ImageJ	National Institutes of Health https://imagej.nih.gov/ij/	RRID:SCR_003070
GraphPad Prism 5 or 7	https://www.graphpad.com	n/a
Origin-Pro 2015	Microcal Origin, Northampton, MA	n/a
pCLAMP 9	Molecular Devices	n/a

### Contact for Reagent and Resource Sharing

Further information and requests for resources and reagents should be directed to and will be fulfilled where possible by the Lead Contact, Annette Dolphin (a.dolphin@ucl.ac.uk).

### Experimental Model and Subject Details

#### Cell lines

Cell lines were plated onto cell culture flasks, coverslips coated with poly-L-lysine, and cultured in a 5% CO_2_ incubator at 37°C. tsA-201 cells (ECACC, female sex) were cultured in Dulbecco’s modified Eagle’s medium in the presence of 10% fetal bovine serum, penicillin, streptomycin and 2% GlutaMAX (Invitrogen). N2A cells (ATCC, male sex) used for immunocytochemistry experiments, were cultured in DMEM and OPTI-MEM (1:1), supplemented with FBS (5%), penicillin (1 unit/ml), streptomycin (1 μg/ml), and GlutaMAX (1%).

#### Primary Hippocampal cultures

Hippocampal neurons were obtained from P0 rat pups (Sprague-Dawley, male), as previously described (Morales et al., 2000). All experiments were performed in accordance with the UK Home Office Animals (Scientific procedures) Act 1986, using a Schedule 1 method, with UCL ethical approval. Briefly, hippocampi were dissected and treated for 40 min at 37°C with a papain solution containing: 70 units /ml of papain, 0.2 mg/ml L-cysteine, 0.2 mg/ml bovine serum albumin (BSA), 1 mg/ml DNase and 5mg/ml glucose (all from Sigma Aldrich) in Hank’s basal salt solution (HBSS) medium (Invitrogen). Hippocampi were then washed twice with plating solution (Neurobasal medium supplemented with B27 (Thermo Fisher Scientific; 2%), HEPES (10 mM), horse serum (5%), glutamine (0.5 mM) and 1 unit/ml penicillin, 1 μg/ml streptomycin), and the neurons were mechanically dissociated using fire-polished glass Pasteur pipettes with decreasing diameter. Approximately 75 × 10^3^ cells in 100 μl of plating solution were seeded onto sterile poly-lysine-coated glass coverslips. After 2 h, the plating solution was replaced with 1 ml of growth medium (serum-free Neurobasal medium supplemented with B27 (4%), 2-mercaptoethanol (25 μM), glutamine (0.5 mM), and 1 unit/ml penicillin, 1 μg/ml streptomycin), half of which was replaced every 3-4 days. At 7 days *in vitro* and 2 h before transfection, half of the medium was removed, and kept as ‘conditioned’ medium, and 500 μl of fresh medium was added.

### Method Details

#### Molecular biology and constructs

cDNAs encoding the following proteins were used: calcium channel Ca_V_2.2 (rabbit, GenBank: D14157), containing an extracellular HA tag (Cassidy et al., 2014), β1b (rat, GenBank: X61394), α_2_δ-1 (rat, GenBank: M86621), HA-tagged α_2_δ-1 (Kadurin et al., 2012), rat Ca_v_2.1 (GenBank: M64373), human TASK3 (KCNK9) cDNA (GenBank: NM_001282534) and mCherry. Zebrafish zCachd1 was cloned from a zebrafish cDNA library. Rat rCachd1 cDNA (GenBank: NM_001191758) was purchased from OriGene. Note that the *Cachd1* gene was misnamed *Cacna2d4* in the original bioinformatics paper in which it was identified as α_2_δ-like (Whittaker and Hynes, 2002). All cDNAs were subcloned into the expression vectors pMT2, pcDNA3 and pCAGGS. In some experiments, Ca_V_2.2-HA also had the green fluorescent protein, mut3bGFP (GFP), fused to the N terminus (Macabuag and Dolphin, 2015). The D122A mutation was introduced into Ca_V_2.2 by mutating aspartate at position 122 of rabbit Ca_V_2.2 to alanine by PCR. C-terminal GFP fusion proteins of both zCachd1 and rCachd1 were made by standard techniques, and used where stated. The sequences of all constructs were confirmed by DNA sequencing.

#### Antibodies and other materials

Ca channel Abs used were: α_2_δ-1 Ab (mouse monoclonal against α_2_-1 moiety, Sigma-Aldrich, epitope identified in (Cassidy et al., 2014)), anti-Ca_V_2.2 II-III loop Ab (rabbit polyclonal) (Raghib et al., 2001). A bespoke, affinity-purified Cachd1 rabbit polyclonal Ab was raised by Cambridge Research Biochemicals (Billingham, UK) against the predicted extracellular domain of zCachd1 protein, produced by transient transfection of mammalian cells (Durocher et al., 2002) (G.T.P., S.W.W., and Gavin J. Wright, unpublished data). Purified Ab activity was confirmed by enzyme-linked immunosorbent assay. Other Abs used were anti-HA (rat monoclonal, Roche), anti-HA (rabbit polyclonal, Sigma), anti-GAPDH Ab (mouse monoclonal, Ambion), and GFP Ab (Living Colors, rabbit polyclonal; BD Biosciences). For immunocytochemistry, secondary Abs (1:500) used were anti-rabbit-Alexa Fluor 594, anti-rat-Alexa Fluor 488, anti-mouse-Alexa Fluor 647 (Life Technologies) or anti-rat fluorescein isothiocyanate (Sigma-Aldrich). The secondary Abs used for Western Blotting were goat anti-rabbit, goat anti-rat, and goat-anti-mouse Abs coupled to horseradish peroxidase (HRP) (Biorad). ω-conotoxin GVIA was purchased from Alomone, and applied by local perfusion.

#### Cell line transfection

For co-IPs and electrophysiological studies, tsA-201 cells were transfected using Fugene6 (Promega, Fitchburg, WI) according to the manufacturer’s protocol. For immuno-cytochemistry, tsA-201 cells were transfected using PolyJet (SignaGen) according to the manufacturer’s protocol. N2A cells were re-plated onto poly-lysine coated coverslips and transfections were carried out using PolyJet (SignaGen) at a ratio of 3:1 to DNA mix according to manufacturer’s instructions. For all electrophysiology and imaging experiments transfections, the cDNA mix consisted of cDNAs encoding WT or D122A Ca_V_2.2, β1b, α_2_δ-1 in a ratio of 3:2:2. The α_2_δ-1 was replaced with Cachd1 or empty vector where appropriate. When these experiments involved both α_2_δ-1 and Cachd1, Ca_V_2.2, β1b, α_2_δ-1 and Cachd1 were added in a ratio of 3:2:2:2, with empty vector replacing α_2_δ-1 or Cachd1 where appropriate. For co-IP experiments Ca_V_2.2 (with or without GFP and HA tags, as stated), β1b and α_2_δ-1 were transfected in a ratio of 2:1:2. For co-IP competition experiments the transfection mix contained Ca_V_2.2: β1b: α_2_δ-1: (TASK3, Cachd1 or a 1:1 mix of both) in a ratio of 2:1:2:1. For reverse co-IP experiments, Cachd1_GFP, β1b, and Ca_V_2.2 were transfected in a ratio of 2:1:2.

#### Neuronal transfection

The hippocampal cultures were then transfected using Lipofectamine 2000, at a ratio of 1:2 to DNA mix (1 μg/μl). After 2 h, the transfection mixes were replaced with growth medium consisting of 50% conditioned and 50% fresh medium. The DNA mix consisted of cDNAs in pCAGGS encoding WT Ca_V_2.2 or D122A Ca_V_2.2, α_2_δ-1, β1b and mCherry, at a ratio of 3:2:2:0.5. α_2_δ-1 was replaced by empty vector or rCachd1 when appropriate.

#### Cell surface biotinylation, cell lysis, deglycosylation and immunoblotting

The procedures were modified from those described in more detail previously (Kadurin et al., 2012, Kadurin et al., 2016). Briefly, 72 h after transfection, tsA-201 cells were incubated for 30 min at room temperature with 0.5 mg/ml Premium Grade EZ-link Sulfo-NHS-LC-Biotin (Thermo Scientific) in PBS and the reaction was quenched with 200 mM glycine. The cells were resuspended in PBS, pH 7.4 at 4°C containing 1% Igepal; 0.1% SDS and protease inhibitors (PI, cOmplete, Roche), to allow cell lysis, cleared by centrifugation at 18,000 × g and assayed for total protein (Bradford assay, Biorad). Cleared WCL corresponding to 20 – 40 μg total protein was mixed with Laemmli sample buffer (Davies et al., 2010) supplemented with 100 mM dithiothreitol (DTT), resolved by SDS-polyacrylamide gel electrophoresis (PAGE) on 3%–8% Tris-Acetate (Invitrogen) and transferred to polyvinylidene fluoride (PVDF) membrane (Biorad). The proteins were revealed by immunoblotting performed with the corresponding Abs essentially as described previously (Kadurin et al., 2012). The signal was obtained by HRP reaction with fluorescent product (ECL 2; Thermo Scientific) and membranes were scanned on a Typhoon 9410 phosphorimager (GE Healthcare). Biotinylated lysates (equalized to between 0.5 and 1 mg/ml total protein concentration) were applied to 40 μl prewashed streptavidin-agarose beads (Thermo Scientific) and rotated overnight at 4°C. The beads were then washed 3 times with PBS containing 0.1% Igepal and, when required, the streptavidin beads were deglycosylated for 3 h at 37°C with 1 unit of PNGase F (Roche Applied Science). The samples containing precipitated cell surface protein fractions were then analyzed by immunoblotting with the indicated Abs as described previously (Kadurin et al., 2012).

#### Co–Immunoprecipitation

The protocol described below was adapted from a procedure described previously (Gurnett et al., 1997). A tsA-201 cell pellet derived from one confluent 75 cm^2^ flask was resuspended in co-IP buffer (20 mM HEPES (pH 7.4), 300 mM NaCl, 1% Digitonin and PI), sonicated for 8 s at 20 kHz and rotated for 1 h at 4°C. The samples were then diluted with an equal volume of 20 mM HEPES (pH 7.4), 300 mM NaCl, 1 mM CaCl_2_, 1 mM MgCl_2_, with PI (to 0.5% final concentration of Digitonin), mixed by pipetting and centrifuged at 20,000 x g for 20 min. The supernatants were collected and assayed for total protein (Bradford assay; Biorad). 1 mg of total protein was adjusted to 2 mg/ml with co-IP buffer and incubated overnight at 4°C with anti-GFP polyclonal Ab (1:200; BD Biosciences). 30 μl A/G PLUS Agarose slurry (Santa Cruz) was added to each tube and further rotated for 2 h at 4°C. The beads were then washed three times with co-IP buffer containing 0.2% Digitonin. The beads were then resuspended in 2 x Laemmli buffer with 100 mM DTT and analyzed alongside equalized aliquots of the initial lysate prior to co-IP by SDS-PAGE and western blotting as described above. The reverse co-IP experiments between Cachd1_GFP and Ca_V_2.2-HA were performed under identical conditions except that the NaCl concentration in the co-IP buffer was 150 mM, and the beads were washed two times in co-IP buffer containing 0.1% Digitonin.

#### Immunocytochemistry

Cells were fixed with 4% paraformaldehyde in phosphate-buffered saline (PBS, pH 7.4) for 5 min, incubated with blocking buffer (20% goat serum, 4% BSA in PBS) for 1 h at room temperature before being incubated with rat anti-HA (Roche) diluted 1:200 in 0.5x blocking buffer for 1 h at room temperature. When permeabilization was included, cells were permeabilized with 0.2% Triton X-100 for 5 min before being incubated with the second primary Ab, rabbit anti-Ca_V_2.2 II-II loop (1:250), for 1 h at room temperature. For hippocampal neurons, primary Ab incubation was carried out at 4°C overnight. After washing, samples were incubated with secondary Abs, anti-rat Alexa Fluor 488, anti-rat Alexa Fluor 594 and anti-rabbit Alexa Fluor 488, at a dilution of 1:500 for 1 h at room temperature. 4’,6-diamidine-2′-phenylindole dihydrochloride (DAPI) was used to visualize the nuclei. Coverslips were washed and mounted in VectaShield (Vector Laboratories).

#### Endocytosis assay

N2A cells were transfected with a Ca_V_2.2 construct tagged with a double bungarotoxin binding site epitope (Ca_V_2.2-BBS) (Cassidy et al., 2014), β1b and either empty vector, α_2_δ-1 or rCachd1. After 40 h expression, cells were washed twice with Krebs-Ringer solution with HEPES (KRH) (in mM; 125 NaCl, 5 KCl, 1.1 MgCl_2_, 1.2 KH_2_PO_4_, 2 CaCl_2_, 6 Glucose, 25 HEPES, 1 NaHCO_3_) and incubated with 10 μg/ml α-bungarotoxin Alexa Fluor® 488 conjugate (BTX-488) (Thermo Fisher Scientific) at 17°C for 30 min. The unbound BTX-488 was removed by washing with KRH, and the labeled cells were returned to 37°C for the kinetic assay. Endocytosis was terminated by fixing the cells with cold 4% PFA-sucrose in PBS at the specified time. The cells were then permeabilized and intracellular Ca_V_2.2 was labeled using the rabbit anti-Ca_V_2.2 II-III loop Ab as described above.

#### Image Analysis

N2A and tsA-201 cell samples were viewed on an LSM 780 confocal microscope (Zeiss) using either 63x/1.4 or 40x/1.3 numerical aperture oil-immersion objective in 16-bit mode. The tile function (3x3 tiles, each tile consisting of 1024x1024 pixels) was used and every transfected cell within the image was analyzed to remove collection bias. Hippocampal neurons were viewed using a 20x objective (neuronal processes) or 63x objective (soma); individual neurons were selected on the basis of mCherry expression. Acquisition settings, chosen to ensure that images were not saturated, were kept constant for each experiment. Images are individual optical sections, unless otherwise stated.

Images were analyzed using ImageJ (National Institutes of Health). For N2A cells, the freehand line tool (5 pixels) was used to manually trace the plasma membrane to measure the mean intensity of cell-surface staining. Intracellular staining was measured using the freehand selection tool, excluding the nucleus and the plasma membrane. For hippocampal neurons, two concentric circles (100 and 150 μm diameter) were centered on the soma and the freehand line tool (3 pixels) was used to trace the neuronal processes between the circles, using the mCherry image as the template. The background fluorescence was measured in an area with no transfected cells and subtracted from the mean intensity.

#### Electrophysiology

Calcium channel currents in transfected tsA-201 cells were investigated by whole cell patch-clamp recording. The patch pipette solution contained in mM: Cs-aspartate, 140; EGTA, 5; MgCl_2_, 2; CaCl_2_, 0.1; K_2_ATP, 2; HEPES, 10; pH 7.2, 310 mOsm with sucrose. The external solution for recording Ba^2+^ currents contained in mM: tetraethylammonium (TEA) Br, 160; KCl, 3; NaHCO_3_, 1.0; MgCl_2_, 1.0; HEPES, 10; glucose, 4; BaCl_2_, 1, pH 7.4, 320 mOsm with sucrose. 1 mM extracellular Ba^2+^ was the charge carrier. Pipettes of resistance 2-4 MΩ were used. An Axopatch 1D or Axon 200B amplifier was used, and whole cell voltage-clamp recordings were sampled at 10 kHz frequency, filtered at 2 kHz and digitized at 1 kHz. 70%–80% series resistance compensation was applied, and all recorded currents were leak subtracted using P/8 protocol. Membrane potential was held at – 80 mV. Analysis was performed using pCLAMP 9 (Molecular Devices) and Origin 7 (Microcal Origin, Northampton, MA). *IV* relationships were fit by a modified Boltzmann equation as follows: *I = G*_*max*_*^∗^(V-V*_*rev*_*)/(1+exp(-(V-V*_*50, act*_*)/k))* where *I* is the current density (in pA/pF), *G*_max_ is the maximum conductance (in nS/pF), *V*_rev_ is the apparent reversal potential, *V*_50, act_ is the midpoint voltage for current activation, and *k* is the slope factor.

### Quantification and Statistical Analysis

Data were analyzed with GraphPad Prism 7 (GraphPad software, San Diego, CA) or Origin-Pro 2015 (OriginLab Corporation, Northampton, MA, USA). All data are shown as mean ± SEM; “n” refers to number of cells or neurites, unless indicated otherwise, and is given in the figure legends, together with details of statistical tests used. Experiments where representative data are shown were repeated at least 3 times, unless otherwise stated. Graphpad Prism 7 was used for statistical analysis. Statistical significance between two groups was assessed by Student’s t test, as stated. One-way ANOVA and the stated post hoc analysis was used for comparison of means between three or more groups.

## Supplemental Information

Document S1. Figures S1–S4

Document S2. Article plus Supplemental Information
