# Supplementary material for: The α2δ-like Protein Cachd1 Increases N-type Calcium Currents and Cell Surface Expression and Competes with α2δ-1
Source: Cell Rep. 2018 Nov 6;25(6):1610–1621.e5. doi: 10.1016/j.celrep.2018.10.033 (PMC6231325; doi:10.1016/j.celrep.2018.10.033)
Supplement: Document S1. Figures S1–S4 [file mmc1.pdf]

Cell Reports, Volume *25*

Supplemental Information

The $\alpha_2\delta$-like Protein Cachd1 Increases N-type Calcium Currents and Cell Surface Expression and Competes with $\alpha_2\delta$-1

Shehrazade Dahimene, Karen M. Page, Ivan Kadurin, Laurent Ferron, Dominique Y. Ho, Gareth T. Powell, Wendy S. Pratt, Stephen W. Wilson, and Annette C. Dolphin

SUPPLEMENTARY FIGURES

Figure S1 (relates to Figure 1): Cachd1 is a membrane protein and sequence of rat Cachd1 showing predicted N-glycosylation sites.

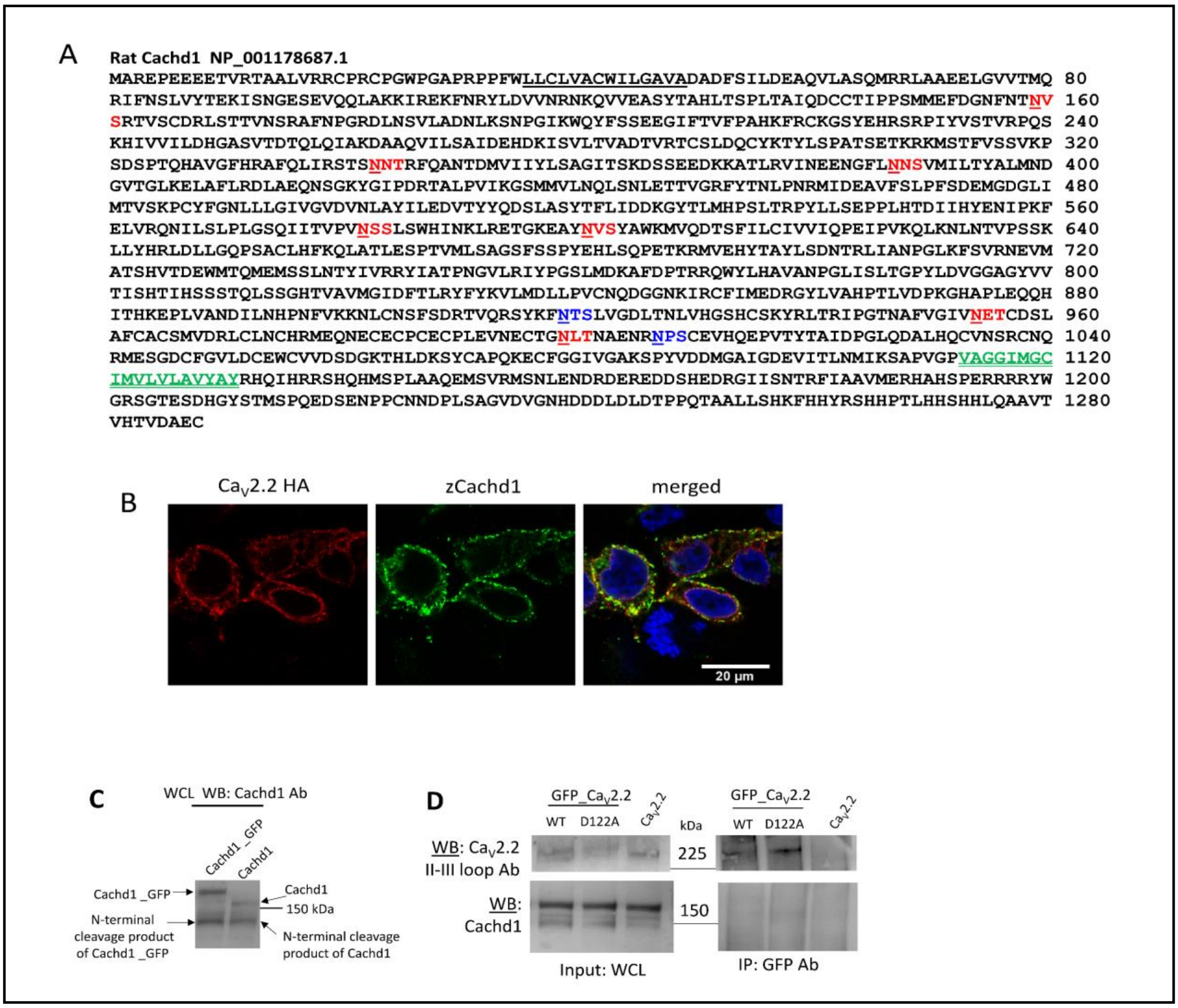

(A) Sequence of rCachd1. Highly predicted N-glycosylation NxS/T sequences are shown in red, sequences with a sub-threshold prediction level are in blue. The approximate predicted position of the N-terminal signal sequence is underlined, and the approximate predicted position of the transmembrane segment is underlined in green. Predictions are from Signal P4.1, NetNglyc 1.0 and ExPASy TMpred.
(B) Representative confocal images of tsA-201 cells expressing $Ca_V2.2$ HA WT (left) with β1b and zCachd1. Cells were permeabilized, and incubated with rat anti-HA and rabbit anti-Cachd1 Abs for 1 h to show distribution of HA staining (left panel, red) and Cachd1 (middle panel, green). Merged images (with co-localization in yellow) are shown in the right-hand panel; DAPI was used to stain the nuclei (blue). Scale bars are 20 µm.
(C) WCL input for experiment shown in Figure 1G, blotted with Cachd1 Ab. zCachd1_GFP (left lane) and untagged zCachd1 (right lane). The Cachd1 Ab shows two bands for both species, whereas the GFP Ab shows a single band for zCachd1_GFP (Figure 1G), indicating that the lower band (which is the same MW in both lanes) is a cleavage product containing the N-terminus of the protein.
(**D**) WCL input (left panels) and IP (right panels) for WT and D122A mutant GFP_$Ca_V2.2$, and untagged $Ca_V2.2$ control (upper panels), and for rCachd1 (lower panels). IP was performed with GFP Ab, and pulled down both WT and D122A GFP_$Ca_V2.2$ (upper right panel). Lack of co-IP of Cachd1 is shown in lower right panel. Representative of 3 experiments.

Figure S2 (relates to Figure 2): Effect of D122A mutation of $Ca_V2.2$ on $Ca_V2.2$ currents enhancement by $\alpha_2\delta$-3

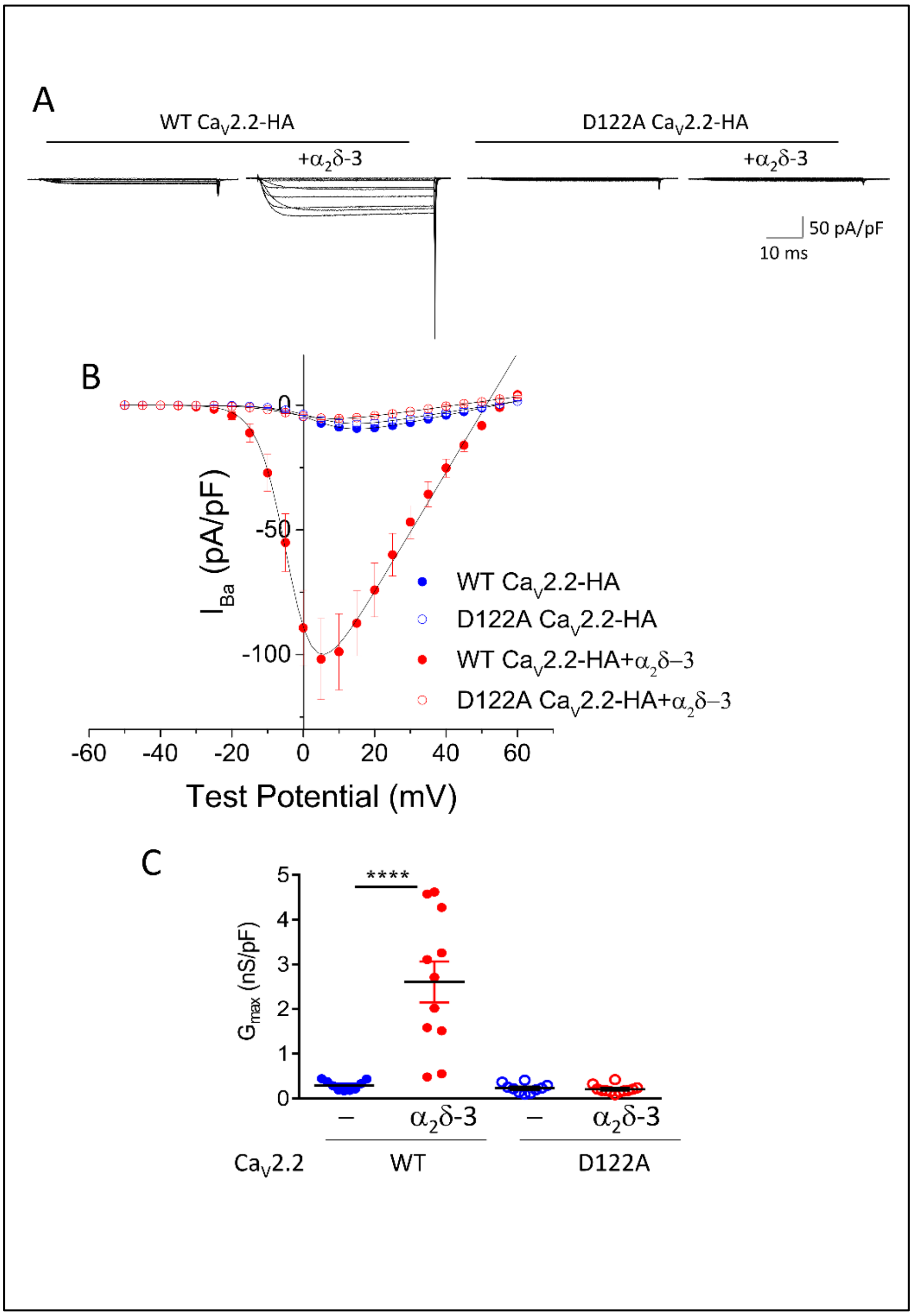

(A) Example families of $Ca_V2.2$ currents for WT $Ca_V2.2$-HA (left two) and D122A $Ca_V2.2$-HA (right two), co-expressed with β1b and either no $\alpha_2\delta$ (left), $\alpha_2\delta$-3 (right). Holding potential -80 mV, steps between -50 and +60 mV for 50 ms. Calibration bars apply to all traces.

(B) Mean (± SEM) current-voltage relationships for the conditions shown in (A). WT $Ca_V2.2$-HA (solid circles) and D122A $Ca_V2.2$-HA (open circles), co-expressed with β1b and either no $\alpha_2\delta$ (blue) or $\alpha_2\delta$-3 (red). The individual and mean data were fit with a modified Boltzmann equation (see Methods).

(C) G_{max} (nS/pF) from the current-voltage relationships shown in (B). Individual data (same symbols as B) and mean ± SEM are plotted. ****P<0.0001.

Figure S3 (relates to Figure 2G): Cachd1 does not increase Ca_V2.1 currents

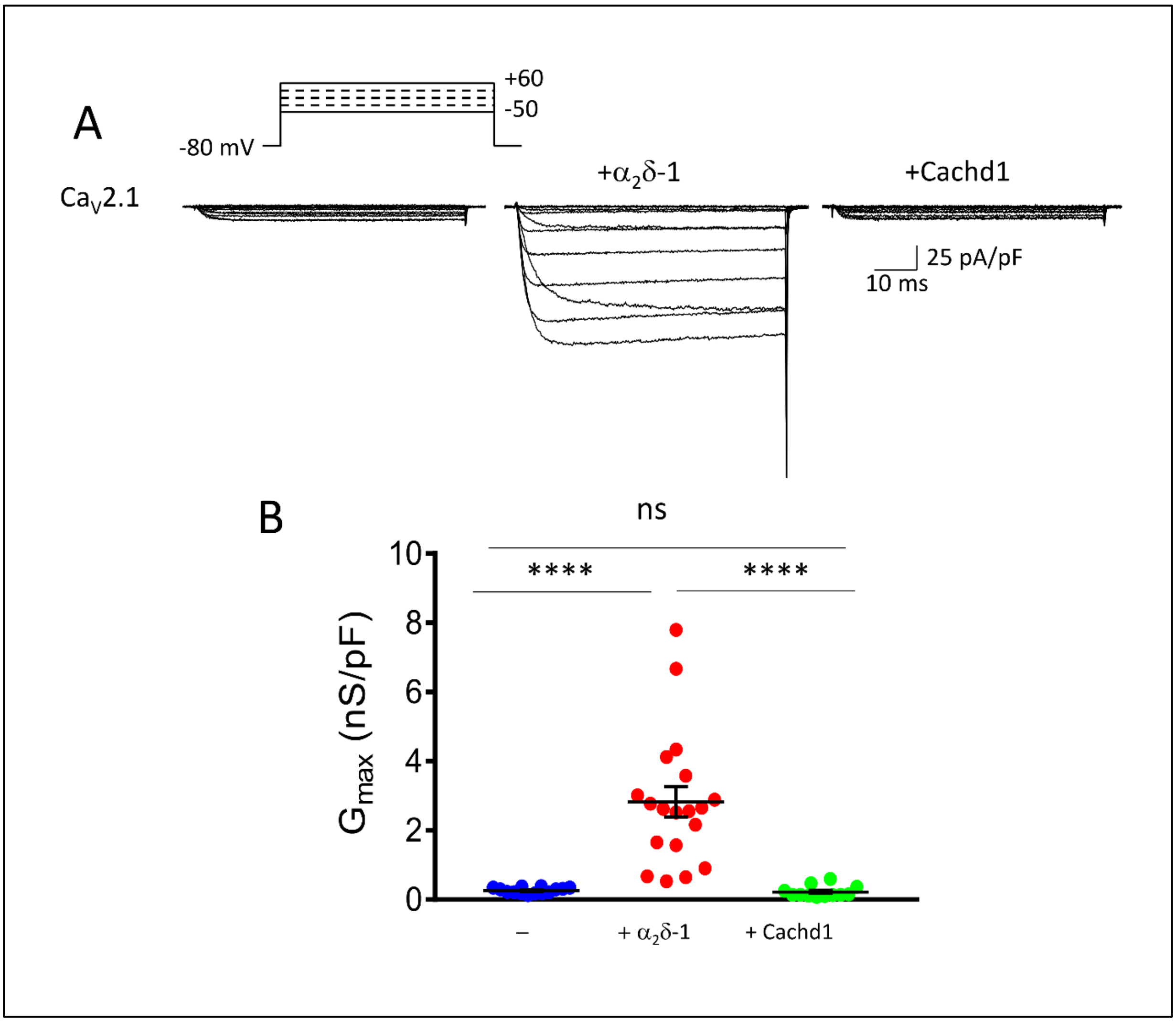

(A) Example current traces of Ca_V2.1 co-expressed with β1b and either no $\alpha_2\delta$ (left), $\alpha_2\delta$-1 (middle) or Cachd1 (right). Holding potential -80 mV, steps between -50 and +60 mV for 50 ms.

(B) Maximum conductance G_{max} (nS/pF) from the current-voltage relationships shown in Figure 2G. Individual data for Ca_V2.1 co-expressed with β1b and either no $\alpha_2\delta$ (blue solid circles), $\alpha_2\delta$-1 (red solid circles) or Cachd1 (green solid circles) and mean ± SEM are plotted. ns, not significant; **** P<0.0001 (one way ANOVA and Sidak's post-hoc test correcting for multiple comparisons). A similar result was observed for zCachd1 (data not shown).

Figure S4 (relates to Figure 3): $\alpha_2\delta$-1 and Cachd1 expression and effect on $Ca_V2.2$ cell surface expression

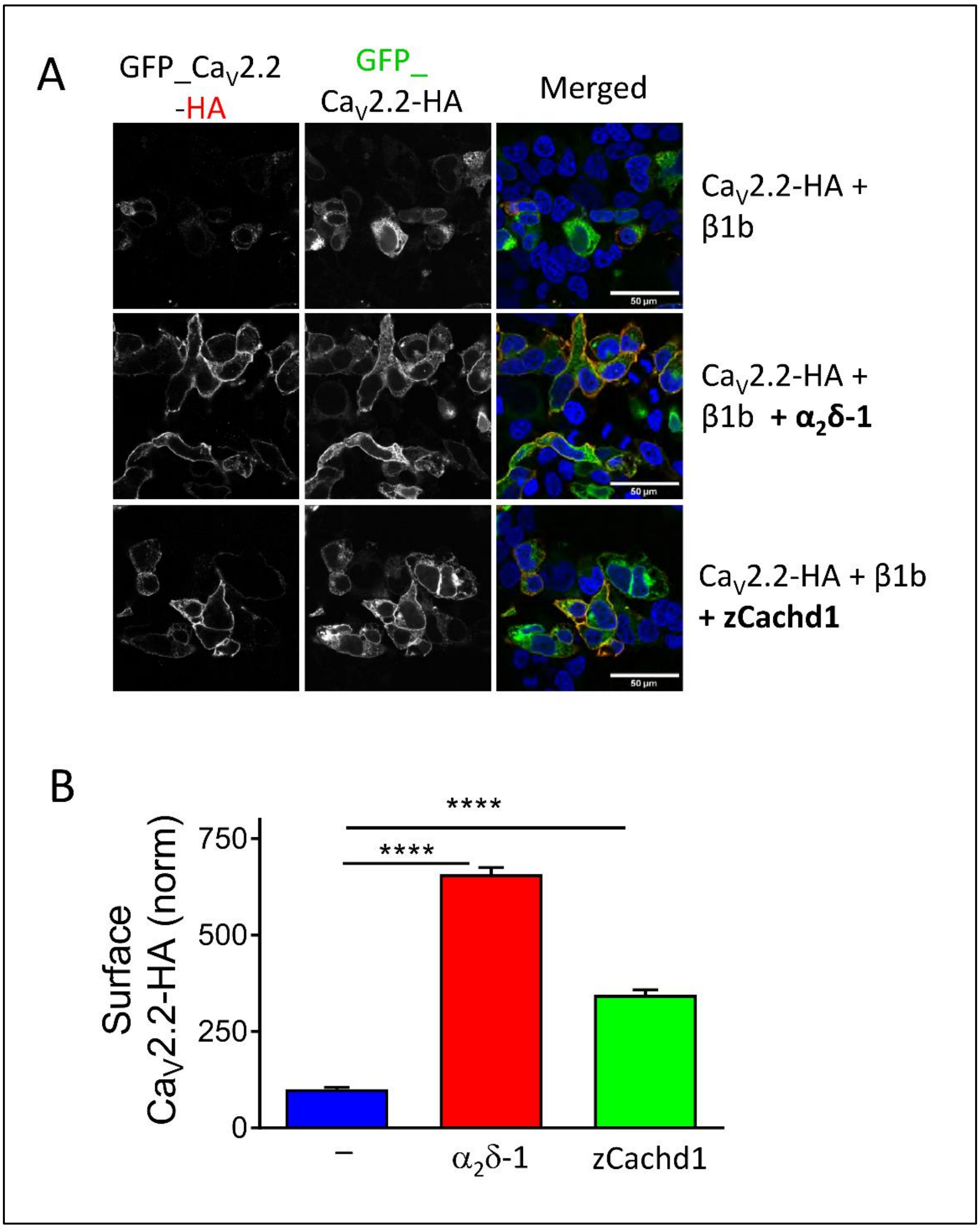

(A) Representative confocal images of tsA-201 cells expressing GFP_$Ca_V2.2$-HA WT with β1b in the absence of $\alpha_2\delta$ (control, top row) with $\alpha_2\delta$-1 (middle row) or zCachd1 (bottom row). Cells were not permeabilized and incubated with rat anti-HA antibody for 1 h to show extracellular HA staining on the plasma membrane (left panels, white), to be compared with intracellular GFP fluorescence (middle panels). Merged images (with HA in red) are shown in the right-hand panels; DAPI was used to stain the nuclei (blue). Scale bars are 50 µm.

(B) Bar chart showing cell surface expression of $Ca_V2.2$-HA, determined by HA staining in the absence of permeabilization. Control condition without $\alpha_2\delta$ or zCachd1 (blue, normalized to 100%), with $\alpha_2\delta$-1 (red) and zCachd1 (green). Data for 901 (- $\alpha_2\delta$-1), 921 (+ $\alpha_2\delta$-1), 970 (+ zCachd1) cells were normalized to the $Ca_V2.2$-HA condition in each experiment. **** $P<0.0001$ (1-way ANOVA and Sidak's post-hoc test correcting for multiple comparisons).
