# Supplementary material for: The α2δ-like Protein Cachd1 Increases N-type Calcium Currents and Cell Surface Expression and Competes with α2δ-1
Source: Cell Rep. 2018 Nov 6;25(6):1610–1621.e5. doi: 10.1016/j.celrep.2018.10.033 (PMC6231325; doi:10.1016/j.celrep.2018.10.033)
Supplement: Document S2. Article plus Supplemental Information [file mmc2.pdf]

Cell Reports

Article

The $\alpha_2\delta$-like Protein Cachd1 Increases N-type Calcium Currents and Cell Surface Expression and Competes with $\alpha_2\delta$-1

Graphical Abstract

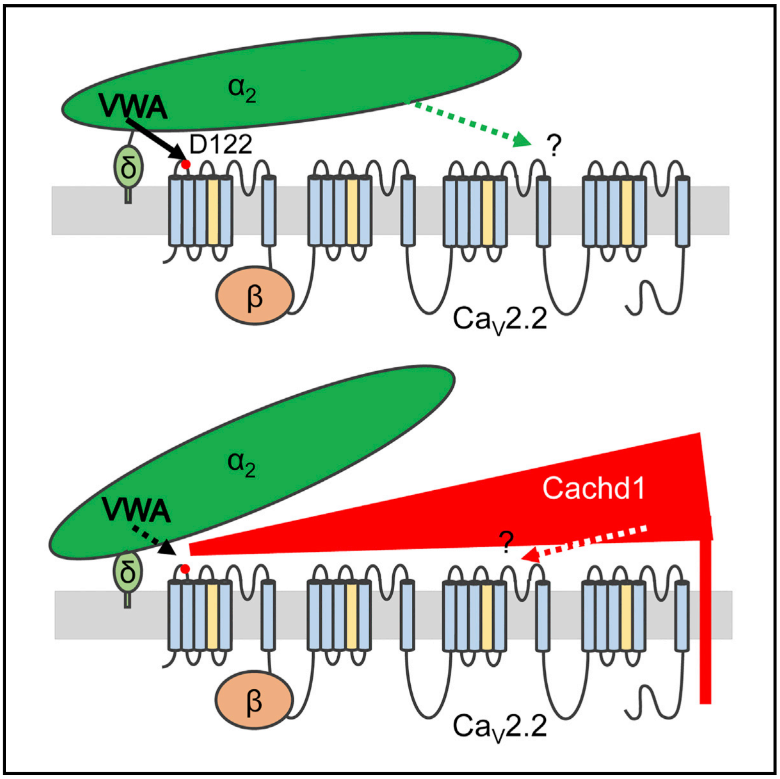

Highlights

- Cachd1 enhances $Ca_V2.2$ currents and increases $Ca_V2.2$ surface expression
- Effects of Cachd1 are not prevented by mutation in $Ca_V2.2$ VWA interaction site
- The effects of $\alpha_2\delta$-1 are prevented by the same mutation in $Ca_V2.2$
- Cachd1 competes with $\alpha_2\delta$-1 for its effects on $Ca_V2.2$

Authors

Shehrazade Dahimene, Karen M. Page, Ivan Kadurin, ..., Wendy S. Pratt, Stephen W. Wilson, Annette C. Dolphin

Correspondence

a.dolphin@ucl.ac.uk

In Brief

Dahimene et al. examine the role of Cachd1, a protein with similarity to the auxiliary $\alpha_2\delta$ subunits of voltage-gated calcium channels. They find that Cachd1 increases N-type calcium currents substantially despite having a disrupted VWA interaction domain. Cachd1 also enhances channel trafficking and inhibits responses to $\alpha_2\delta$-1.

Dahimene et al., 2018, Cell Reports *25*, 1610–1621
November 6, 2018 © 2018 The Authors.
https://doi.org/10.1016/j.celrep.2018.10.033

OPEN ACCESS
CellPress

Cell Reports
Article

The $\alpha_2\delta$-like Protein Cachd1 Increases N-type Calcium Currents and Cell Surface Expression and Competes with $\alpha_2\delta$-1

Shehrazade Dahimene,[1,3] Karen M. Page,[1,3] Ivan Kadurin,[1] Laurent Ferron,[1] Dominique Y. Ho,[1] Gareth T. Powell,[2] Wendy S. Pratt,[1] Stephen W. Wilson,[2] and Annette C. Dolphin[1,4,*]
[1]Department of Neuroscience, Physiology and Pharmacology, Division of Biosciences, University College London, London WC1E 6BT, UK
[2]Department of Cell and Developmental Biology, Division of Biosciences, University College London, London WC1E 6BT, UK
[3]These authors contributed equally
[4]Lead Contact
*Correspondence: a.dolphin@ucl.ac.uk
https://doi.org/10.1016/j.celrep.2018.10.033

SUMMARY

Voltage-gated calcium channel auxiliary α2δ subunits are important for channel trafficking and function. Here, we compare the effects of α2δ-1 and an α2δ-like protein called Cachd1 on neuronal N-type ($Ca_V2.2$) channels, which are important in neurotransmission. Previous structural studies show the α2δ-1 VWA domain interacting with the first loop in $Ca_V1.1$ domain-I via its metal ion-dependent adhesion site (MIDAS) motif and additional Cache domain interactions. Cachd1 has a disrupted MIDAS motif. However, Cachd1 increases $Ca_V2.2$ currents substantially (although less than α2δ-1) and increases $Ca_V2.2$ cell surface expression by reducing endocytosis. Although the effects of α2δ-1 are abolished by mutation of Asp122 in $Ca_V2.2$ domain-I, which mediates interaction with its VWA domain, the Cachd1 responses are unaffected. Furthermore, Cachd1 co-immunoprecipitates with $Ca_V2.2$ and inhibits co-immunoprecipitation of α2δ-1 by $Ca_V2.2$. Cachd1 also competes with α2δ-1 for effects on trafficking. Thus, Cachd1 influences both $Ca_V2.2$ trafficking and function and can inhibit responses to α2δ-1.

INTRODUCTION

Voltage-gated calcium (Ca_V) channels are key constituents of excitable cells, including muscles, neurons, and secretory cells, and are essential for their function (for a review, see Zamponi et al., 2015). The neuronal N-type ($Ca_V2.2$) and P/Q-type ($Ca_V2.1$) channels are critical for presynaptic release of neurotransmitters (for a review, see Nanou and Catterall, 2018), with N-type calcium channels playing a particularly important role in primary afferent neurotransmission involving pain pathways (for a review, see McGivern and McDonough, 2004). Ca_V α1 subunits form the pore of the channels, determining their main biophysical and pharmacological properties (Zamponi et al., 2015), but the associated β and $\alpha_2\delta$ proteins represent auxiliary subunits that are important contributors to the trafficking and biophysical properties of the channel complexes (Gurnett et al., 1996; Leung et al., 1987; Pragnell et al., 1994; Takahashi et al., 1987). The β subunits increase Ca_V currents by binding to the intracellular I-II linker (Pragnell et al., 1994), promoting folding (Van Petegem et al., 2004), hyperpolarizing current activation (Stea et al., 1993), preventing polyubiquitination (Page et al., 2016), and inhibiting proteasomal degradation (Altier et al., 2011; Waithe et al., 2011).

By contrast, the mechanism by which the $\alpha_2\delta$ subunits increase trafficking and function of channel complexes is less well understood (Cantí et al., 2005; Cassidy et al., 2014; Ferron et al., 2018; Kadurin et al., 2016; Savalli et al., 2016). The $\alpha_2\delta$-1 subunit, in combination with neuronal calcium channels, is the therapeutic target for gabapentinoid drugs, used for the alleviation of neuropathic pain conditions and as an add-on therapy in certain epilepsies (Field et al., 2006), and it is therefore important to understand its mechanism of action. The $\alpha_2\delta$ proteins undergo several post-translational processing steps, including N-glycosylation, proteolytic cleavage into α_2 and δ (De Jongh et al., 1990; Ellis et al., 1988; Jay et al., 1991), and glycosyl-phosphatidylinositol (GPI) anchoring (Davies et al., 2010).

The recent structure of the skeletal muscle $Ca_V1.1$ complex (Wu et al., 2016) has revealed a complex interaction of $\alpha_2\delta$-1 with several extracellular loops in domains I-III of $Ca_V1.1$. In the present study, we have taken advantage of the insights provided by this structure to probe the role of the von Willebrand factor A (VWA) domain and investigate whether there is a role for other $\alpha_2\delta$ domains in Ca_V channel function. In previous studies, by mutating the metal ion-dependent adhesion site (MIDAS) motif in the VWA domain of $\alpha_2\delta$ subunits, we have shown that the VWA domains of both $\alpha_2\delta$-1 and $\alpha_2\delta$-2 are key to promoting calcium channel trafficking and function (Cantí et al., 2005; Cassidy et al., 2014; Hoppa et al., 2012). The structure confirms the interaction of the MIDAS motif with the $Ca_V1.1$ α1 subunit (Wu et al., 2016). However, we also found that mutating the MIDAS motif reduced the trafficking of $\alpha_2\delta$-1 itself when it was expressed alone (Cassidy et al., 2014). In the present study, we have therefore taken the reciprocal step of mutating the residue in $Ca_V2.2$ with which $\alpha_2\delta$-1 is predicted to bind to examine whether other regions, such as their Cache domains, play a role in promoting

This is an open access article under the CC BY license (http://creativecommons.org/licenses/by/4.0/).

$Ca_V2.2$ trafficking and function. The Cache domains in $\alpha_2\delta$-1, which have homology to domains in bacterial chemotaxis receptors (Anantharaman and Aravind, 2000), have also been shown to interact with the $Ca_V1.1$ $\alpha1$ subunit (Wu et al., 2016). We have compared the effect of $\alpha_2\delta$-1 with that of Cachd1, identified bioinformatically to be related to $\alpha_2\delta$ proteins (Whittaker and Hynes, 2002). Cachd1 has a VWA domain with a disrupted MIDAS motif but retains multiple predicted Cache domains. Surprisingly, we found that expression of Cachd1 increased both $Ca_V2.2$ currents and cell surface trafficking in both cell lines and neurons. By contrast, expression of Cachd1 did not increase the closely related $Ca_V2.1$ currents, indicating that this effect shows specificity for certain calcium channels. Furthermore, Cachd1 competed with $\alpha_2\delta$-1 for binding to $Ca_V2.2$ and for its functional effects and can therefore inhibit responses to $\alpha_2\delta$-1.

RESULTS

Disruption of the Interaction Site between $Ca_V2.2$ and the $\alpha_2\delta$-1 VWA Domain Prevents the Interaction between $\alpha_2\delta$-1 and $Ca_V2.2$

In previous studies, we found that mutation of the MIDAS motif in $\alpha_2\delta$-1 and $\alpha_2\delta$-2 prevented the ability of these proteins to traffic Ca_V2 channels and abolished the increase in Ca_V1 and Ca_V2 currents, normally seen with wild-type (WT) $\alpha_2\delta$-1 and $\alpha_2\delta$-2 (Cantí et al., 2005; Cassidy et al., 2014; Hoppa et al., 2012). However, trafficking of the $\alpha_2\delta$-1 MIDAS mutant alone to the cell surface was also impaired (Cassidy et al., 2014), and our data indicate that $\alpha_2\delta$-1 also interacts with the trafficking protein LRP1 via its VWA domain (Kadurin et al., 2017). Therefore, in the present study, we took advantage of the recently described structure of the skeletal muscle calcium channel complex (Wu et al., 2016) and mutated the residue in $Ca_V2.2$ likely to coordinate the divalent cation together with the MIDAS interaction site of $\alpha_2\delta$-1. The structure of $Ca_V1.1$ shows this to be residue D78, which is in the first extracellular loop of domain I; it corresponds by alignment to D122 in $Ca_V2.2$ (Figures 1A and 1B). This residue was mutated to uncharged alanine to disrupt the interaction with $\alpha_2\delta$-1. D122A $Ca_V2.2$ was expressed at the same level as WT $Ca_V2.2$ in tsA-201 cells in the presence of β1b and $\alpha_2\delta$-1 (Figure 1C). As we found previously (Kadurin et al., 2016), $Ca_V2.2$ showed robust co-immunoprecipitation with $\alpha_2\delta$-1 (Figures 1C and 1D). In contrast, D122A $Ca_V2.2$ exhibited only very weak co-immunoprecipitation (coIP) with $\alpha_2\delta$-1 (Figures 1C and 1D), confirming a key role for D122 in this interaction.

The $\alpha_2\delta$ Homolog Cachd1 Is Expressed on the Cell Surface and Interacts with $Ca_V2.2$

The cryoelectron microscopy (cryo-EM) structure of $Ca_V1.1$ shows that $\alpha_2\delta$-1 has four Cache domains (Anantharaman and Aravind, 2000; Wu et al., 2016), and there are interactions of the $\alpha1$ subunit with these domains as well as with the VWA domain (Wu et al., 2016). The $\alpha_2\delta$-like protein Cachd1 contains Cache domains, similar to the $\alpha_2\delta$ subunits, but its VWA domain has a highly disrupted MIDAS motif (Whittaker and Hynes, 2002). Indeed, in a preliminary report, Cachd1 was found to have no effect on $Ca_V2.2$ currents (Soubrane et al., 2012). Because our experiments also suggest that the VWA domain has a dominant role in mediating the effects of $\alpha_2\delta$-1, we decided to investigate whether Cachd1 showed any residual functional effect on $Ca_V2.2$ function.

We initially used a construct encoding zebrafish Cachd1 (zCachd1) that had been generated in a study to identify genes underlying particular nervous system development phenotypes (H. Stickney, A. Faro, G.T.P., and S.W.W., unpublished data). We subsequently confirmed our results with the rat construct rCachd1. There is very high sequence conservation, the two proteins being 85.6% identical at the amino acid level. Using a polyclonal antibody (Ab) raised against the predicted extracellular domain of zCachd1, which also recognizes human CACHD1 (G.T.P., G.J. Wright, and S.W.W., unpublished data), we observed a major band of the predicted molecular weight (MW) in whole-cell lysate (WCL) of tsA-201 cells transfected to express either zCachd1 or rCachd1 (Figure 1E). Cachd1 is predicted to be an N-glycosylated protein (Figure S1A). For rCachd1, the MW was ~168 kDa when glycosylated and ~148 kDa following deglycosylation with N-Glycosidase F (PNGase F), indicating that it has up to 7 N-glycosylation sites (Figure 1E), agreeing with the predicted number (Figure S1A). The glycosylation pattern is also compatible with the prediction that Cachd1 is a type I membrane protein (Figure S1A), in contrast to the GPI-anchored $\alpha_2\delta$ proteins (Davies et al., 2010). In addition to the major Cachd1 protein band, two lower MW minor bands were observed. For rCachd1, these were ~148 and ~137 kDa, reduced to ~133 and ~119 kDa following deglycosylation (Figure 1E). Similar results were found for zCachd1 (Figure 1E). Cell surface biotinylation indicated that the major band was the species on the plasma membrane (Figure 1E), suggesting that membrane-associated Cachd1 does not undergo post-translational proteolytic processing, unlike $\alpha_2\delta$ proteins.

To determine whether Cachd1 was co-localized on the cell surface with $Ca_V2.2$, we expressed the proteins in N2A or tsA-201 cells and imaged their localization. We found that both rCachd1 (Figure 1F) and zCachd1 (Figure S1B) were present on the cell surface, together with either WT $Ca_V2.2$ or D122A $Ca_V2.2$ and β1b. Partial co-localization of Cachd1 with $Ca_V2.2$-hemagglutinin (HA) on the cell surface was observed (Figure 1F, yellow regions). Even in permeabilized cells, most of the Cachd1 appeared to be associated with the cell surface (Figure S1B).

We then co-expressed $Ca_V2.2$ with a C-terminally GFP-tagged Cachd1 and found that immunoprecipitation (IP) of Cachd1_GFP with GFP Ab was able to coIP $Ca_V2.2$. As a control, there was no coIP of $Ca_V2.2$ using Cachd1 without a GFP tag (Figure 1G), expression of which was confirmed using Cachd1 Ab (Figure S1C). The interaction of Cachd1 with $Ca_V2.2$ was likely to be weaker than that observed for $\alpha_2\delta$-1 because no coIP of Cachd1 with GFP_$Ca_V2.2$ was observed in experiments performed under conditions similar to those shown for $\alpha_2\delta$-1 in Figure 1C (Figure S1D).

The D122A Mutation in $Ca_V2.2$ Prevents the Effect of $\alpha_2\delta$-1 but Not Cachd1 on $Ca_V2.2$ Currents

In agreement with the coIP results, we found that expression of rCachd1 produced a consistent increase (4.5-fold) in WT $Ca_V2.2$ currents (in the additional presence of β1b) despite its disrupted MIDAS motif (Figures 2A–2C).

A

$Ca_V1.1$ #	70	71	72	73	74	75	76	77	78	79	80	81
Rabbit $Ca_V1.1$	V	Y	L	P	M	P	E	D	D	N	N	S
Rabbit $Ca_V2.2$	L	E	Q	H	L	P	D	G	D	K	T	P
$Ca_V2.2$ #	114	115	116	117	118	119	120	121	122	123	124	125

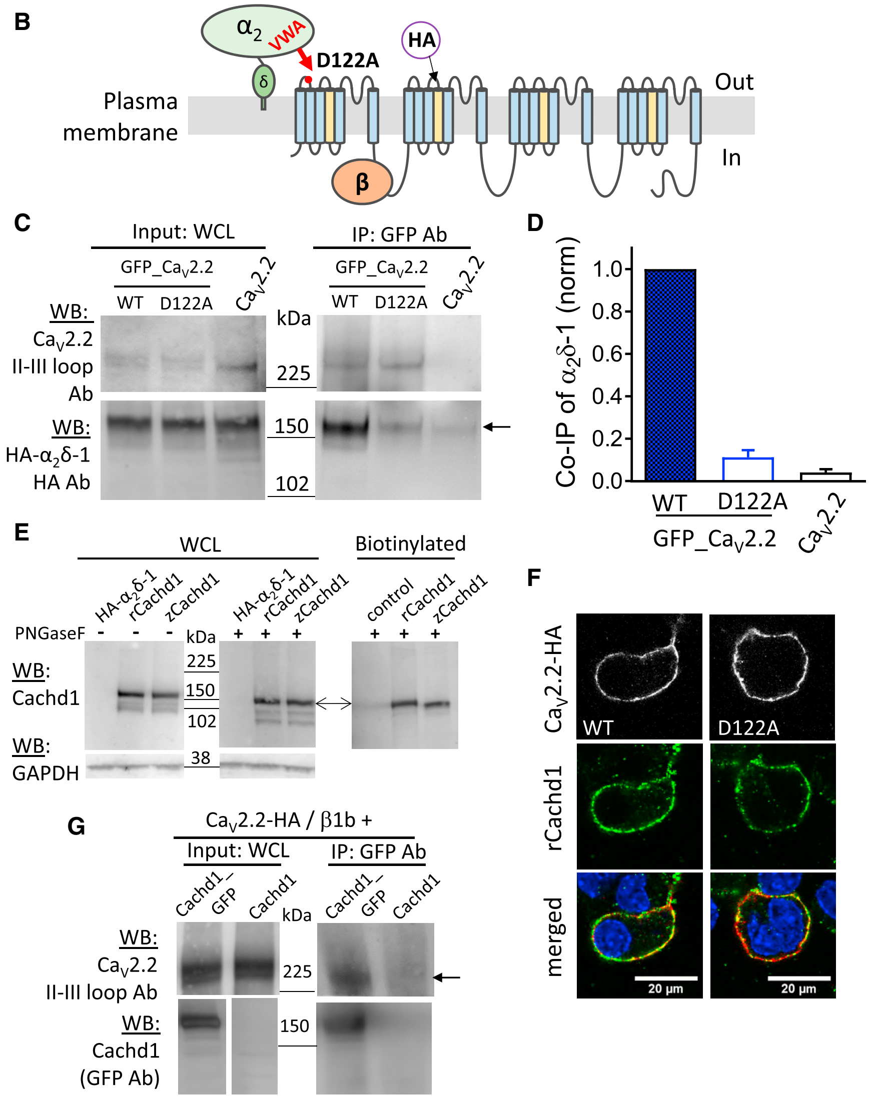

Figure 1. Effect of D122A Mutation in $Ca_V2.2$ on Interaction with $\alpha_2\delta$-1 and Cachd1

(A) Sequence alignment of the VWA domain interaction site on $Ca_V1.1$ in comparison with the rabbit $Ca_V2.2$ used in this study, showing the position of D122 in the first extracellular loop of $Ca_V2.2$. Residue numbering is shown (#).

(B) Diagram of the putative $Ca_V2.2$ interaction site with the VWA domain of $\alpha_2\delta$-1, showing the position of the D122A mutation and the HA epitope tag.

(C) IP of GFP_$Ca_V2.2$, and co-immunoprecipitation (coIP) of $\alpha_2\delta$-1. WCL input (left) and IP (right) for WT and D122A mutant GFP_$Ca_V2.2$ and untagged $Ca_V2.2$ control (top) and for HA-tagged $\alpha_2\delta$-1 (bottom). IP was performed with GFP Ab and pulled down both WT and D122A GFP_$Ca_V2.2$ (top right). CoIP of HA-tagged $\alpha_2\delta$-1 is shown in at the bottom right (arrow).

(D) Quantification of coIP of $\alpha_2\delta$-1 with WT GFP_$Ca_V2.2$ (solid blue bar) compared with D122A GFP_$Ca_V2.2$ (open blue bar) and control $Ca_V2.2$ (open black bar); mean ± SEM of 5 experiments.

(E) Western blot using Cachd1 Ab of WCL from tsA-201 cells transfected with $\alpha_2\delta$-1 as a control (lane 1), rCachd1 (lane 2), and zCachd1 (lane 3). Left: prior to deglycosylation with PNGase F. Center: after deglycosylation. Bottom: glyceraldehyde 3-phosphate dehydrogenase (GAPDH) loading control. Right: a separate experiment after cell surface biotinylation and deglycosylation; the control here was untransfected cells. The arrow indicates a major Cachd1 band.

(F) Representative confocal images of N2A cells expressing $Ca_V2.2$ HA WT (left) or D122A (right) with β1b and rCachd1. Cells were not permeabilized and incubated with rat anti-HA and rabbit anti-Cachd1 Abs for 1 hr to show extracellular HA staining on the plasma membrane (top row, white) and Cachd1 (center row, green). Merged images (with HA in red and co-localization in yellow) are shown at the bottom; DAPI was used to stain the nuclei (blue). Scale bars, 20 μm.

(G) IP of Cachd1_GFP and coIP of $Ca_V2.2$. Shown are WCL input (left) and IP (right). Top: $Ca_V2.2$. Bottom: zCachd1_GFP (left lane) and untagged zCachd1 (right lane; both lanes are from the same blot). IP was performed with GFP Ab and pulled down both Cachd1_GFP (bottom) and $Ca_V2.2$ (top right, arrow). Lack of coIP of $Ca_V2.2$ with untagged Cachd1 is shown in the right lane. Data are representative of n = 6 experiments. zCachd1 expression in WCL is confirmed in Figure S1C.

We then examined the effect of the D122A mutation on the ability of $\alpha_2\delta$-1 and Cachd1 to increase $Ca_V2.2$ currents. We found that, although $\alpha_2\delta$-1 increased the maximum conductance (G_{max}) of WT $Ca_V2.2$ by 11.5-fold, it produced no increase in the case of D122A $Ca_V2.2$, for which the currents were of the same amplitude as WT $Ca_V2.2$ without $\alpha_2\delta$ (Figures 2A–2C). Very similar results to those observed with $\alpha_2\delta$-1 were obtained for $\alpha_2\delta$-3 (Figure S2).

By contrast, we found that Cachd1 produced a similar increase (5.2-fold) in G_{max} for D122A $Ca_V2.2$ to that observed for WT $Ca_V2.2$ (Figures 2A–2C). This result indicates that the effect of Cachd1 is unlikely to be dependent on co-ordination of a divalent cation between its disrupted MIDAS motif and loop I of the α1 subunit and, therefore, might involve other interactions with Cachd1. Like $\alpha_2\delta$-1, Cachd1 induced a shift of current activation to more hyperpolarized potentials for both WT and D122A $Ca_V2.2$, as shown in the current-voltage (I-V) relationships (Figure 2B).

It is noteworthy that, for both WT $Ca_V2.2$ and D122A $Ca_V2.2$, we observed that the barium current (I_{Ba}) in the presence of rCachd1 had an apparent reversal potential that was ~11.6 mV more negative compared with WT $Ca_V2.2$ currents in the

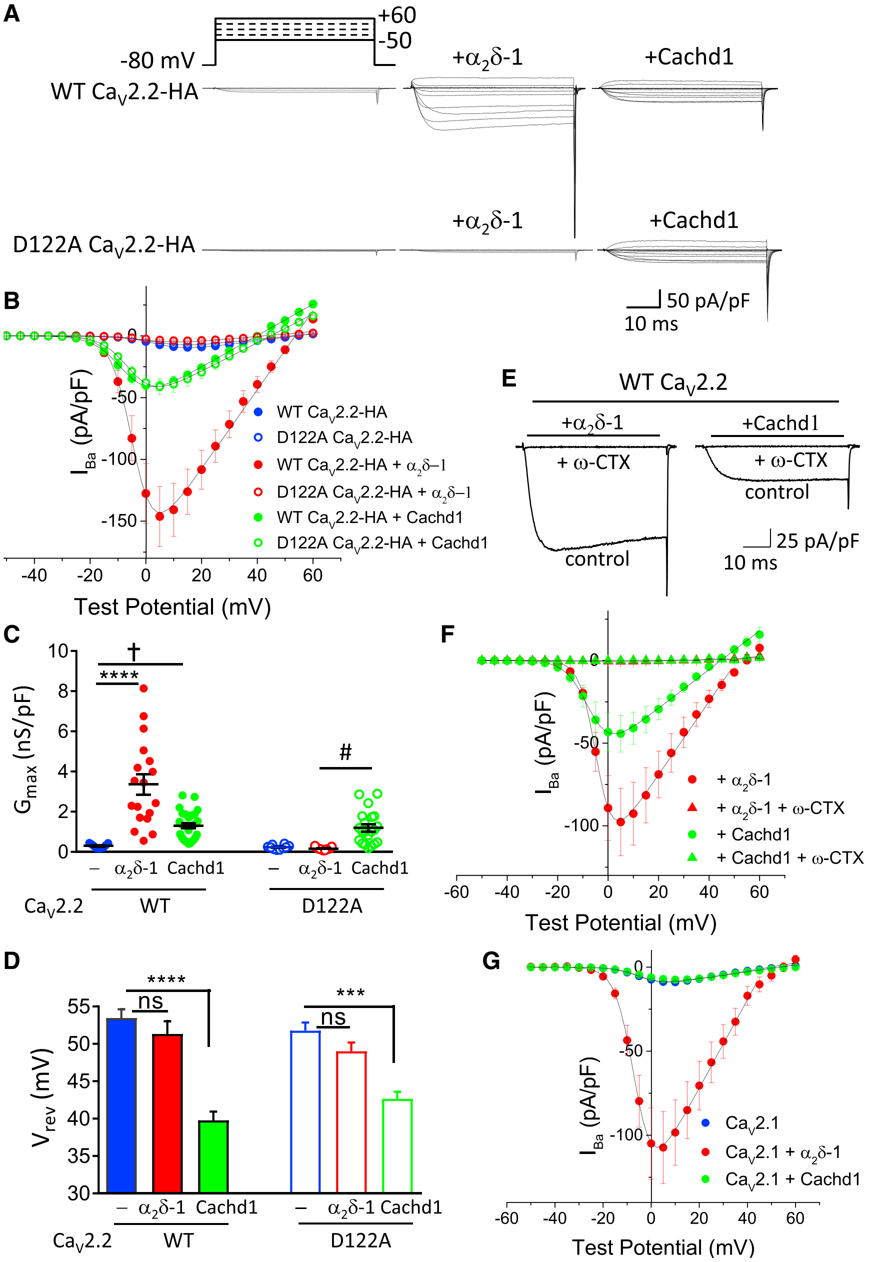

Figure 2. D122A Mutation of $Ca_V2.2$ Abolishes $Ca_V2.2$ Current Enhancement by $\alpha_2\delta$-1 but Not Cachd1

(A) Example families of $Ca_V2.2$ currents for WT $Ca_V2.2$-HA (top row) and D122A $Ca_V2.2$-HA (bottom row), co-expressed with β1b and either no $\alpha_2\delta$ (left), $\alpha_2\delta$-1 (center), or rCachd1 (right). Holding potential −80 mV, steps between −50 and +60 mV for 50 ms (top, applies to all traces).

(B) Mean (± SEM) current-voltage relationships for the conditions shown in (A). WT $Ca_V2.2$-HA (solid circles; n = 9, 18, and 27 for no $\alpha_2\delta$, $\alpha_2\delta$-1, and Cachd1, respectively) and D122A $Ca_V2.2$-HA (open circles; n = 10, 11, and 21 for no $\alpha_2\delta$, $\alpha_2\delta$-1, and Cachd1, respectively) were co-expressed with β1b and either no $\alpha_2\delta$ (blue), $\alpha_2\delta$-1 (red), or Cachd1 (green). The individual and mean data were fit with a modified Boltzmann equation (STAR Methods). The potential for half-maximal activation ($V_{50,act}$) (mV) was +4.9 ± 1.3, −2.9 ± 1.6, and −4.6 ± 0.5 for WT $Ca_V2.2$-HA with no $\alpha_2\delta$, $\alpha_2\delta$-1 and Cachd1, respectively, and +4.5 ± 0.7, +3.7 ± 0.9, and −3.6 ± 0.7 for D122A $Ca_V2.2$-HA with no $\alpha_2\delta$, $\alpha_2\delta$-1, and Cachd1, respectively.

(C) G_{max} (nanosiemens [nS]/picofarad [pF]) from the current-voltage relationships shown in (B). Individual data (same symbols as in B) and mean ± SEM are plotted. †p = 0.0483, #p = 0.0357, ****p < 0.0001 (1-way ANOVA and Sidak's *post hoc* test correcting for multiple comparisons).

(D) Bar charts of mean ± SEM for reversal potential (V_{rev}) (millivolt) for the conditions shown in (B). WT $Ca_V2.2$-HA (solid bars) and D122A $Ca_V2.2$-HA (open bars) were co-expressed with β1b and either no $\alpha_2\delta$ (blue), $\alpha_2\delta$-1 (red), or Cachd1 (green). ns, not significant; ***p < 0.001, ****p < 0.0001 (1-way ANOVA and Sidak's *post hoc* test correcting for multiple comparisons).

(E) Examples of current traces at +5 mV of WT $Ca_V2.2$ co-expressed with β1b and either $\alpha_2\delta$-1 (left) or Cachd1 (right) before (control) and after application of 1 μM ω-conotoxin GVIA (+ ω-CTX).

(F) Mean (± SEM) current-voltage relationships before the application of ω-conotoxin GVIA for WT $Ca_V2.2$-HA co-expressed with β1b and either $\alpha_2\delta$-1 (red circles, n = 7) or Cachd1 (green circles, n = 6). The application of ω-conotoxin GVIA (1 μM) produced a complete block of WT $Ca_V2.2$ co-expressed with $\alpha_2\delta$-1 (red triangles, n = 7) or Cachd1 (green triangles, n = 6).

(G) Mean (± SEM) current-voltage relationships for $Ca_V2.1$ co-expressed with β1b and either no $\alpha_2\delta$ (blue solid circles, n = 12), $\alpha_2\delta$-1 (red solid circles, n = 14), or Cachd1 (green solid circles, n = 12). The individual and mean data were fit with a modified Boltzmann equation (STAR Methods).

presence of $\alpha_2\delta$-1, suggesting a possible effect of Cachd1 on ion selectivity (Figure 2D). Under the same recording conditions, no effect was observed of rCachd1, expressed alone, on endogenous conductances in tsA-201 cells, which might independently account for this effect on the reversal potential. Furthermore, the ω-conotoxin GVIA (GVIA) completely abolished $Ca_V2.2$ currents when coexpressed with β1b and rCachd1, as it did when β1b and $\alpha_2\delta$-1 (Figures 2E and 2F). Note that the negative shift in reversal potential induced by Cachd1, relative to $\alpha_2\delta$-1, remains present in this dataset prior to ω-conotoxin GVIA application (Figure 2F).

Surprisingly, rCachd1 did not increase currents through the related $Ca_V2.1$ channel under the same conditions, although $\alpha_2\delta$-1 produced the expected effect (Figure 2G; Figures S3A and S3B), indicating that there is selectivity in the effect of Cachd1 for specific calcium channel isoforms.

The D122A Mutation in $Ca_V2.2$ Reduces the Effect of $\alpha_2\delta$ but Not Cachd1 on $Ca_V2.2$ at the Plasma Membrane

We then compared the cell surface expression of WT and D122A $Ca_V2.2$-HA, either in the presence or absence of $\alpha_2\delta$-1 or Cachd1, using N2A cells. All conditions included the β subunit β1b, and

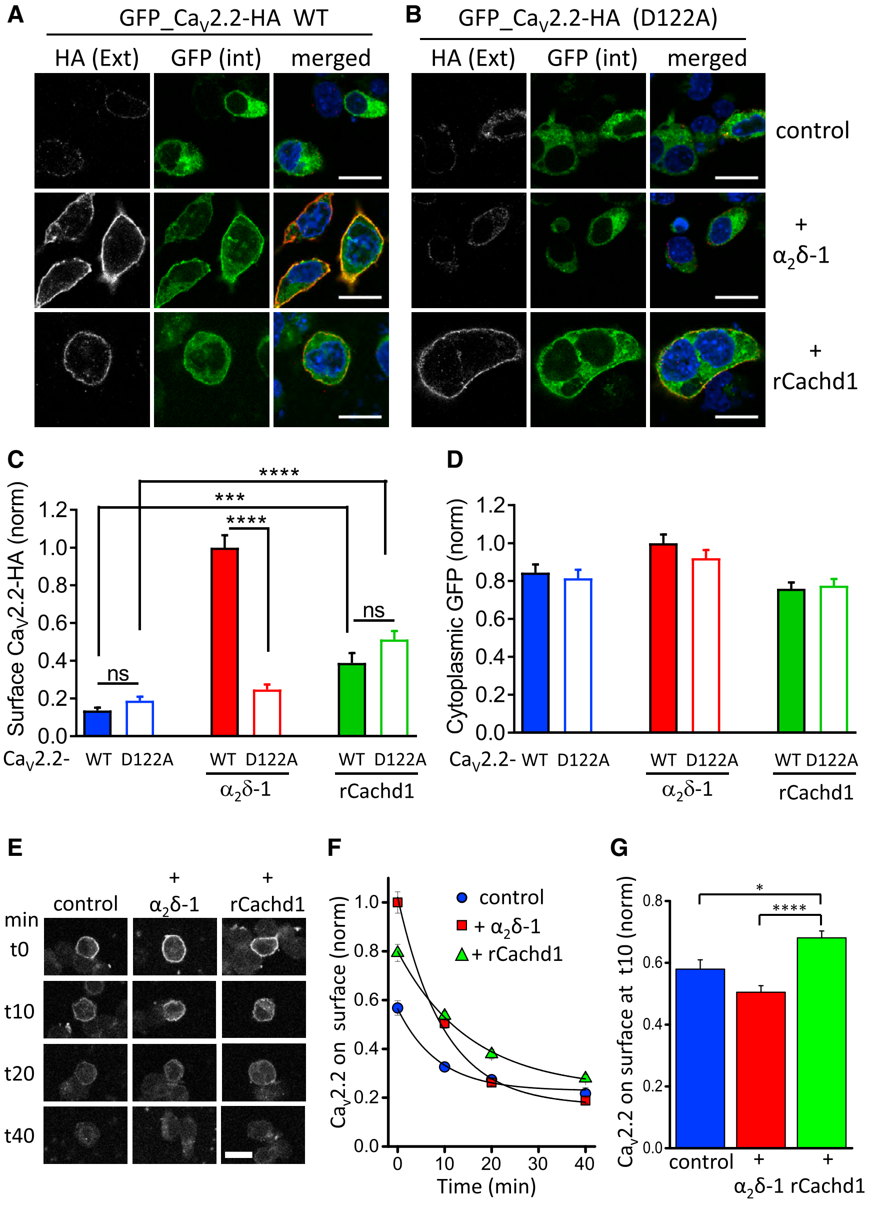

Figure 3. D122A Mutation in $Ca_V2.2$ Prevents Effect of $\alpha_2\delta$-1 but Not Cachd1 on $Ca_V2.2$ Cell Surface Expression in N2A Cells

(A and B) Representative confocal images of N2A cells expressing GFP_$Ca_V2.2$-HA WT (A) or D122A (B) with β1b in the absence of $\alpha_2\delta$ (control, top row) with $\alpha_2\delta$-1 (center row) or rCachd1 (bottom row). Intact cells (non-permeabilized) were incubated with rat anti-HA Ab for 1 hr to visualize extracellular HA staining on the plasma membrane (left, white) to be compared with intracellular GFP fluorescence (center). Merged images (with HA in red) are shown on the right; DAPI was used to stain the nuclei (blue). Scale bars, 20 μm.

(C and D) Bar charts showing cell surface expression of WT (closed bars) and D122A $Ca_V2.2$-HA (open bars), determined by HA staining prior to permeabilization (C), and cytoplasmic expression determined by GFP fluorescence (D). Blue bars are for the control condition without $\alpha_2\delta$ or Cachd1, red bars are with $\alpha_2\delta$-1, and green bars are with rCachd1. Data (mean ± SEM) for 164 (WT − $\alpha_2\delta$-1), 220 (WT + $\alpha_2\delta$-1), 185 (WT + Cachd1), 165 (D122A − $\alpha_2\delta$-1), 203 (D122A + $\alpha_2\delta$-1), and 232 (D122A + rCachd1) cells from 3 experiments were normalized to the WT $Ca_V2.2$-HA + $\alpha_2\delta$-1 condition in each experiment. ***$p < 0.001$, ****$p < 0.0001$ (one-way ANOVA and Sidak's *post hoc* test correcting for multiple comparisons).

(E) Representative confocal images of N2A cells expressing $Ca_V2.2$-bungarotoxin binding site (BBS) and labeled with BTX-488. Cells were co-transfected with β1b and either empty vector (control, left), $\alpha_2\delta$-1 (center), or rCachd1 (right). Cells were incubated at 17°C with BTX-488 for 30 min and then imaged at different time points, from zero (t0) to 40 min (t40). Scale bar, 20 μm.

(F) Time course of endocytosis of cell surface $Ca_V2.2$-BBS in control + β1b alone (blue circles), + $\alpha_2\delta$-1 (red squares), and + rCachd1 (green triangles). BTX-488 fluorescence was normalized to the mean fluorescence of the + $\alpha_2\delta$-1 condition at t0. The results are shown as the mean ± SEM. The number of cells (n) obtained from 5 independent experiments varies from 349 to 789 for each time point and condition. The data were fitted with single exponentials. The time constants of the fits were 8.5 min, 9.9 min, and 15.4 min for control, + $\alpha_2\delta$-1, and + rCachd1, respectively.

(G) Bar chart (mean ± SEM) comparing the reduction of cell surface $Ca_V2.2$-BBS at 10 min for the 3 conditions. BTX-488 fluorescence was normalized to t0 for each condition. BTX-488 fluorescence was reduced by 42% ± 3% for control (blue bar, n = 743 cells), 50% ± 2% for + $\alpha_2\delta$-1 (red bar, n = 646 cells), and 32% ± 2% for + rCachd1 (green bar, n = 784 cells). *$p = 0.0109$, ****$p < 0.0001$ (one-way ANOVA and Bonferroni's *post hoc* test for multiple comparisons).

$Ca_V2.2$-HA was N-terminally GFP-tagged to identify all transfected cells. WT $Ca_V2.2$-HA was well expressed at the cell surface when co-expressed with $\alpha_2\delta$-1, which resulted in a 7.3-fold increase compared with its cell surface expression in the absence of $\alpha_2\delta$ (Figures 3A and 3C). In contrast, D122A $Ca_V2.2$-HA exhibited a very low expression level at the plasma membrane, which was similar in the presence and absence of $\alpha_2\delta$-1 (Figures 3B and 3C). In contrast, intracellular expression of WT or D122A $Ca_V2.2$-HA (Figures 3A and 3B) was not significantly different with and without $\alpha_2\delta$-1 (Figure 3D).

In the same experiment, we also investigated the effect of rCachd1 on cell surface expression of $Ca_V2.2$-HA. We found that it produced an increase of 2.9-fold in cell surface expression of WT $Ca_V2.2$-HA (Figures 3A and 3C). Very similar results were obtained for zCachd1 in tsA-201 cells (Figures S4A and S4B). Of great interest, and similar to its effect on calcium currents, is that rCachd1 increased cell surface expression of D122A $Ca_V2.2$-HA by an extent similar to its effect on WT $Ca_V2.2$-HA (a 2.7-fold increase compared with D122A $Ca_V2.2$-HA alone; Figures 3B and 3C). Intracellular expression of WT or D122A $Ca_V2.2$-HA (Figures 3A and 3B) was not significantly different with or without rCachd1 (Figure 3D).

To understand the mechanism of action of Cachd1, we compared the endocytosis rates of $Ca_V2.2$ in the presence of

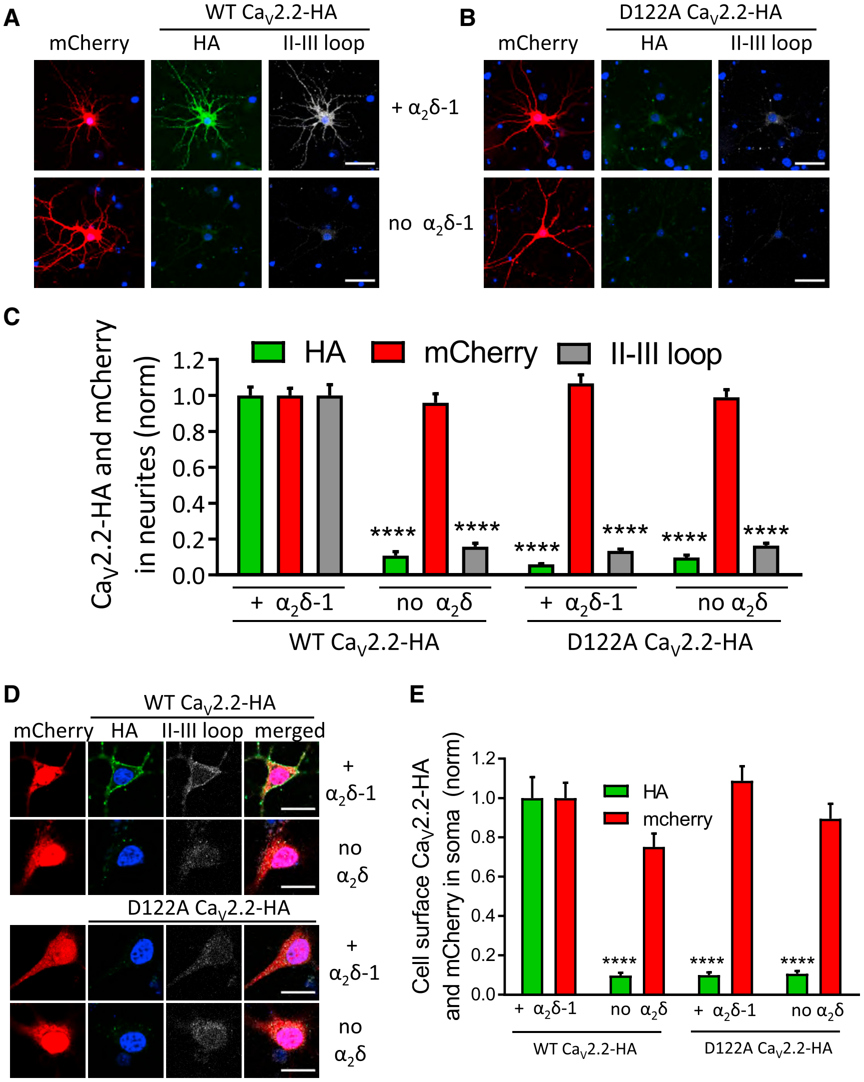

Figure 4. D122A Mutation in $Ca_V2.2$ Prevents the Effect of $\alpha_2\delta$-1 on $Ca_V2.2$-HA Cell Surface Expression in Hippocampal Neurites and Somata

(A) Representative confocal images showing neurites of hippocampal neurons expressing $Ca_V2.2$-HA WT together with β1b and mCherry in the presence (top row) or absence (bottom row) of $\alpha_2\delta$-1. Expression of mCherry is shown in red (left). $Ca_V2.2$-HA (green, center) was stained using rat anti-HA Ab prior to permeabilization, and the rabbit II-III loop Ab (white, right) after permeabilization. DAPI was used to visualize the nucleus (blue). Scale bars, 50 μm.

(B) As for (A) but for hippocampal neurons co-expressing $Ca_V2.2$-HA D122A with β1b and mCherry in the presence (top row) or absence (bottom row) of $\alpha_2\delta$-1.

(C) Bar chart (mean ± SEM) showing expression of WT and D122A $Ca_V2.2$-HA, determined by both HA staining prior to permeabilization (green bars) and II-III loop staining after permeabilization (gray bars), together with the expression marker mCherry. Data for 197 (WT + $\alpha_2\delta$-1), 130 (WT − $\alpha_2\delta$-1), 174 (D122A + $\alpha_2\delta$-1), and 211 (D122A − $\alpha_2\delta$-1) neurites from 4 separate transfections in 2 experiments were normalized to the WT $Ca_V2.2$-HA + $\alpha_2\delta$-1 condition in each experiment. ****$p < 0.0001$ (1-way ANOVA compared with WT $Ca_V2.2$ + $\alpha_2\delta$-1, with Sidak's *post hoc* analysis correcting for multiple comparisons).

(D) Representative confocal images showing hippocampal somata expressing $Ca_V2.2$-HA WT (top two rows) or $Ca_V2.2$-HA D122A (bottom two rows) together with β1b and mCherry in the presence (top row) or absence (bottom row, control) of $\alpha_2\delta$-1. Expression of mCherry is shown in red (first panel). $Ca_V2.2$-HA (green, second panel) was stained using rat anti-HA Ab in non-permeabilized cells, and the rabbit II-III loop Ab (white, third panel) after permeabilization. DAPI was used to visualize the nucleus (blue), and the merged image is shown in the fourth panel. Scale bars, 20 μm.

(E) Bar chart (mean ± SEM) showing expression of WT and D122A $Ca_V2.2$-HA, determined by HA staining prior to permeabilization (green bars), together with expression marker mCherry (red bars). Data for 32 (WT + $\alpha_2\delta$-1), 26 (WT - $\alpha_2\delta$-1), 37 (D122A + $\alpha_2\delta$-1), and 35 (D122A − $\alpha_2\delta$-1) cell bodies from 4 separate transfections in 2 experiments were normalized to the WT $Ca_V2.2$-HA + $\alpha_2\delta$-1 condition in each experiment. ****$p < 0.0001$ (1-way ANOVA compared with WT $Ca_V2.2$ + $\alpha_2\delta$-1, with Sidak's *post hoc* analysis correcting for multiple comparisons).

β1b and either without $\alpha_2\delta$ or plus either $\alpha_2\delta$-1 or Cachd1 (Figures 3E–3G), using a method described previously (Cassidy et al., 2014). We found that Cachd1 reduced the endocytosis rate of $Ca_V2.2$ (Figures 3E–3G). The mean endocytosis time constant was increased from 8.5 min for $Ca_V2.2$ + β1b to 15.4 min in the additional presence of Cachd1 (Figure 3F). This is unlike $\alpha_2\delta$-1, which has no effect on $Ca_V2.2$ endocytosis (Cassidy et al., 2014), a result confirmed here. This effect of Cachd1 on endocytosis may therefore contribute to the increased cell surface expression of $Ca_V2.2$.

The D122A Mutation Abolishes the Effect of $\alpha_2\delta$-1 on the Trafficking of $Ca_V2.2$ into Cultured Hippocampal Neurites

Because we have found the presence of $\alpha_2\delta$ to be a key regulator of trafficking of $Ca_V2.2$ into neuronal processes (Kadurin et al., 2016), we investigated whether the D122A mutation would influence this. Cultured hippocampal neurons were transfected after 7 days in culture, by which time there was already extensive neurite outgrowth. All conditions included β1b and mCherry as a control for successful transfection. After ~7 days of expression, as expected, WT $Ca_V2.2$-HA was strongly trafficked into hippocampal neuronal processes when co-expressed with $\alpha_2\delta$-1 (Figures 4A and S4C). In contrast, there was almost no trafficking of D122A $Ca_V2.2$-HA into hippocampal neurites when co-expressed with $\alpha_2\delta$-1 (Figures 4B and 4C). Its level in the neurites was only 6% of that of WT $Ca_V2.2$-HA with $\alpha_2\delta$-1 (Figure 4C). Similarly, staining the intracellular pool of $Ca_V2.2$ using the II-III linker Ab showed that the level of D122A $Ca_V2.2$-HA was only 13% of WT $Ca_V2.2$-HA (Figure 4C), further indicating that the effect of $\alpha_2\delta$-1 is on trafficking $Ca_V2.2$ into the neurites.

OPEN ACCESS
CellPress

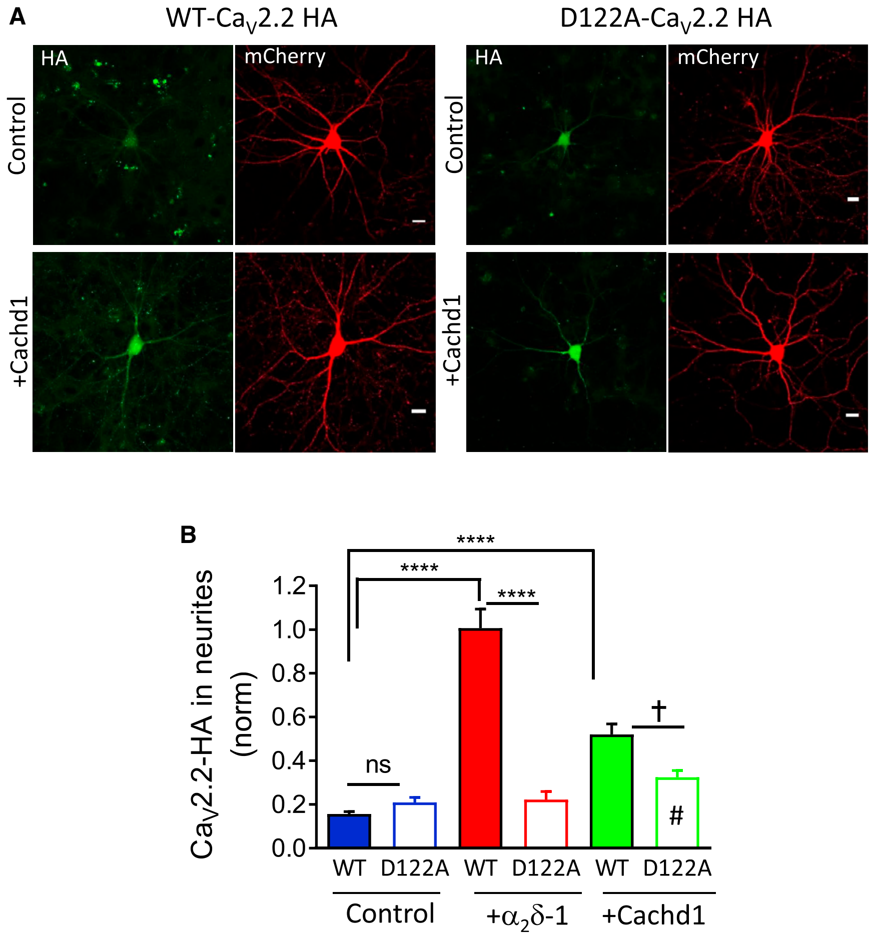

Figure 5. Cachd1 Promotes Ca_V2.2-HA Distribution in Hippocampal Neurites

(A) Representative confocal images showing neurites of hippocampal neurons expressing Ca_V2.2-HA WT (left) or Ca_V2.2-HA D122A (right) together with β1b and mCherry in the absence (top row) or presence (bottom row) of Cachd1. Expression of mCherry is shown in red. Scale bars, 20 μm.

(B) Bar chart (mean ± SEM) showing neurite expression of WT and D122A Ca_V2.2-HA, determined by HA staining of intact cells prior to permeabilization. Shown are data for 137 (WT, blue solid), 144 (D122A, blue open), 200 (WT + $\alpha_2\delta$-1, red solid), 111 (D122A + $\alpha_2\delta$-1, red open), 175 (WT + Cachd1, green solid), and 152 (D122A + Cachd1, green open) neurites from 3 experiments. Data were normalized to the WT Ca_V2.2-HA + $\alpha_2\delta$-1 condition in each experiment. ****p < 0.0001, †p = 0.0473 between WT Ca_V2.2-HA + Cachd1 and D122A Ca_V2.2-HA + Cachd1, #p = 0.5563 between D122A Ca_V2.2-HA and D122A Ca_V2.2-HA + Cachd1 (1-way ANOVA and Sidak's *post hoc* analysis correcting for multiple comparisons).

In the absence of $\alpha_2\delta$-1, there was almost no trafficking of WT Ca_V2.2-HA into hippocampal neurites (Figures 4A and 4C). The same was true for D122A Ca_V2.2-HA, its level being similar in the presence and absence of $\alpha_2\delta$-1 (Figures 4B and 4C).

We also analyzed cell surface expression of Ca_V2.2 in the cell bodies of these hippocampal neurons and found essentially the same result; the increase in cell surface expression resulting from $\alpha_2\delta$-1 was abrogated by the D122A mutation (Figures 4D and 4E), although an intracellular signal was present for both WT and D122A Ca_V2.2-HA (Figure 4D).

Cachd1 Increases the Trafficking of Ca_V2.2 into Hippocampal Neurites

We therefore also investigated the effect of Cachd1 on trafficking of Ca_V2.2-HA into hippocampal neurites (Figure 5A). We found that it produced a consistent increase of WT Ca_V2.2-HA by 3.3-fold (Figures 5A and 5B), although this was less than the 6.8-fold increase produced by $\alpha_2\delta$-1 in the same experiment. However, in this experimental context, Cachd1 was much less able to traffic D122A Ca_V2.2-HA into neurites than WT Ca_V2.2-HA (Figures 5A and 5B), unlike the result observed in the N2A cell line. This result is in agreement with our previous finding that trafficking of Ca_V2.2 is more stringently controlled in neurons than in cell lines (Kadurin et al., 2016).

Cachd1 Competes with $\alpha_2\delta$-1 for Interaction with Ca_V2.2

The preceding experiments indicate that Cachd1 does not utilize the domain I D122 interaction site on Ca_V2.2, which is required by $\alpha_2\delta$-1 for interaction via its MIDAS motif. Because Cachd1 still has a VWA domain, albeit with a disrupted MIDAS motif, we wondered whether Cachd1 might potentially be an antagonist at this site, interfering with the effect of $\alpha_2\delta$-1. We found that Cachd1 concentration-dependently reduced the coIP of $\alpha_2\delta$-1 with GFP_Ca_V2.2, by 71% when both cDNAs were transfected in equal amounts (Figures 6A and 6B), indicating that Cachd1 can obstruct the interaction site on Ca_V2.2 utilized by $\alpha_2\delta$-1.

Furthermore, in experiments measuring Ca_V2.2 cell surface expression (Figures 6C and 6E), the additional presence of Cachd1 significantly reduced the effect of $\alpha_2\delta$-1 on cell surface expression of Ca_V2.2-HA by 28.4% (Figure 6E, purple bar) but had no effect on its intracellular expression (Figure 6F). In this experiment, the increase in cell surface expression of Ca_V2.2-HA in the presence of Cachd1 alone was 54.4% of the Ca_V2.2-HA + $\alpha_2\delta$-1 level (Figure 6E, green bar), and this increase with Cachd1 was still observed for D122A Ca_V2.2 (47.9% of the Ca_V2.2 + $\alpha_2\delta$-1 level; Figure 6E, open green bar). The additional presence of $\alpha_2\delta$-1 had no effect on the increase of D122A Ca_V2.2 cell surface expression in the presence of Cachd1 (Figure 6E, open purple bar).

In a direct parallel with these results, we observed that Cachd1 co-expression significantly reduced Ca_V2.2 currents in the presence of $\alpha2\delta$-1, almost to the level of Ca_V2.2 currents in the presence of Cachd1 alone (Figures 7A and 7B), but $\alpha_2\delta$-1 had no effect on the ability of Cachd1 to increase D122A Ca_V2.2

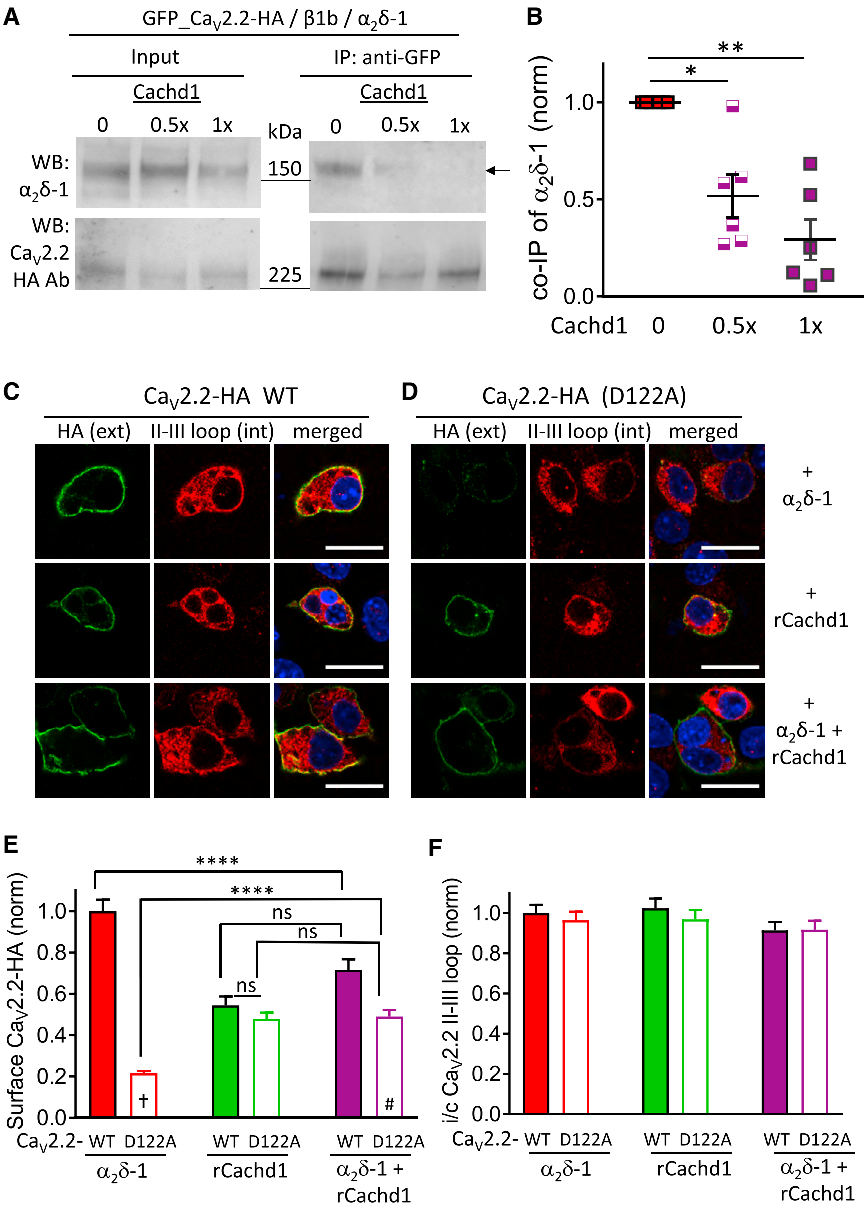

Figure 6. Cachd1 Competes with $\alpha_2\delta$-1 for Interaction with $Ca_V2.2$

(A) IP of GFP_$Ca_V2.2$-HA and coIP of $\alpha_2\delta$-1 in the absence of Cachd1 (left lane, 0; includes TASK3 cDNA as a control) or presence of 0.5× or 1× Cachd1 (center and right lanes, with 0.5× or no TASK3 cDNA, respectively). See STAR Methods for details regarding the cDNA mixes. Shown are WCL input (left) and IP (right) for $\alpha_2\delta$-1 (top) and GFP_$Ca_V2.2$-HA (bottom, detected with HA Ab). The IP was performed with GFP Ab. Co-IP of $\alpha_2\delta$-1 is shown at the top right (arrow).

(B) Scatterplot showing the effect of Cachd1 on $\alpha_2\delta$-1 co-IP with GFP_$Ca_V2.2$-HA for 6 experiments, including that in (A). Shown are no Cachd1 (red squares), 0.5× Cachd1 (purple half-closed squares), and 1× Cachd1 (purple squares). Data are the ratio of $\alpha_2\delta$-1 in IP/input, normalized to no Cachd1 in each experiment. Mean and SEM are also shown; *p = 0.0144, **p = 0.0021 (1-way ANOVA with repeated measures and Sidak's *post hoc* test with multiple comparisons correction, comparing +Cachd1 to no Cachd1).

(C and D) Representative confocal images of N2A cells expressing $Ca_V2.2$-HA WT (C) or D122A (D) with β1b in the presence of $\alpha_2\delta$-1 (top row), rCachd1 (center row), or both $\alpha_2\delta$-1 and rCachd1 (bottom row). Intact cells (non-permeabilized) were incubated with rat anti-HA Ab for 1 hr to show extracellular HA staining on the plasma membrane (left, green). The cells were then permeabilized and stained with the $Ca_V2.2$ II-III loop Ab (center, red). Merged images are shown on the right; DAPI was used to stain the nuclei (blue). Scale bars, 20 μm.

(E and F) Bar charts showing cell surface expression of WT (closed bars) and D122A $Ca_V2.2$-HA (open bars), determined by HA staining prior to permeabilization (E), and cytoplasmic expression determined by II-III loop staining after permeabilization (F). $Ca_V2.2$ with $\alpha_2\delta$-1 is shown in red, with rCachd1 in green, and with both $\alpha_2\delta$-1 and rCachd1 in purple. Data for 328 (WT + $\alpha_2\delta$-1), 255 (WT + rCachd1), 231 (WT + both), 272 (D122A + $\alpha_2\delta$-1), 270 (D122A + rCachd1), and 225 (D122A + both) cells from 3 experiments were normalized to the WT $Ca_V2.2$-HA + $\alpha_2\delta$-1 condition in each experiment. ****p < 0.0001, †p < 0.0001 versus WT, #p = 0.005 versus WT (1-way ANOVA with Sidak's *post hoc* test, comparing all columns and correcting for multiple comparisons).

currents (Figures 7C and 7D). Interestingly, when $\alpha_2\delta$-1 and Cachd1 were co-expressed, the reversal potential for WT $Ca_V2.2$ currents was identical to that observed with $\alpha_2\delta$-1 alone (Figure 7E), pointing to preferential $\alpha_2\delta$-1 interaction on the cell surface. By contrast, for D122A $Ca_V2.2$ currents, the reversal potential in the presence of both $\alpha_2\delta$-1 and Cachd1 was similar to that for Cachd1 alone (Figure 7F), reinforcing the evidence for a lack of interaction of this mutant with $\alpha_2\delta$-1.

DISCUSSION

In this study, we uncovered a mechanism for influencing $Ca_V2.2$ channel trafficking and function mediated by the $\alpha_2\delta$-like protein Cachd1, despite its VWA domain having a disrupted MIDAS motif.

We first established the importance of interaction of $Ca_V2.2$ with the $\alpha_2\delta$-1 VWA domain for its cell surface expression and function by mutating the predicted $\alpha_2\delta$ interaction site in $Ca_V2.2$ (D122), which is in the first extracellular loop of domain I. This mutation completely abolished the ability of $\alpha_2\delta$-1 to increase the trafficking of $Ca_V2.2$ and to increase $Ca_V2.2$ currents, indicating that it is the main interaction site between the channel and $\alpha_2\delta$. This was confirmed by our coIP results.

Surprisingly, Cachd1 consistently produced a 4.5-fold increase in $Ca_V2.2$ currents and also increased the cell surface expression of $Ca_V2.2$ by 2.9-fold. However, in contrast to $\alpha_2\delta$

OPEN
ACCESS
CellPress

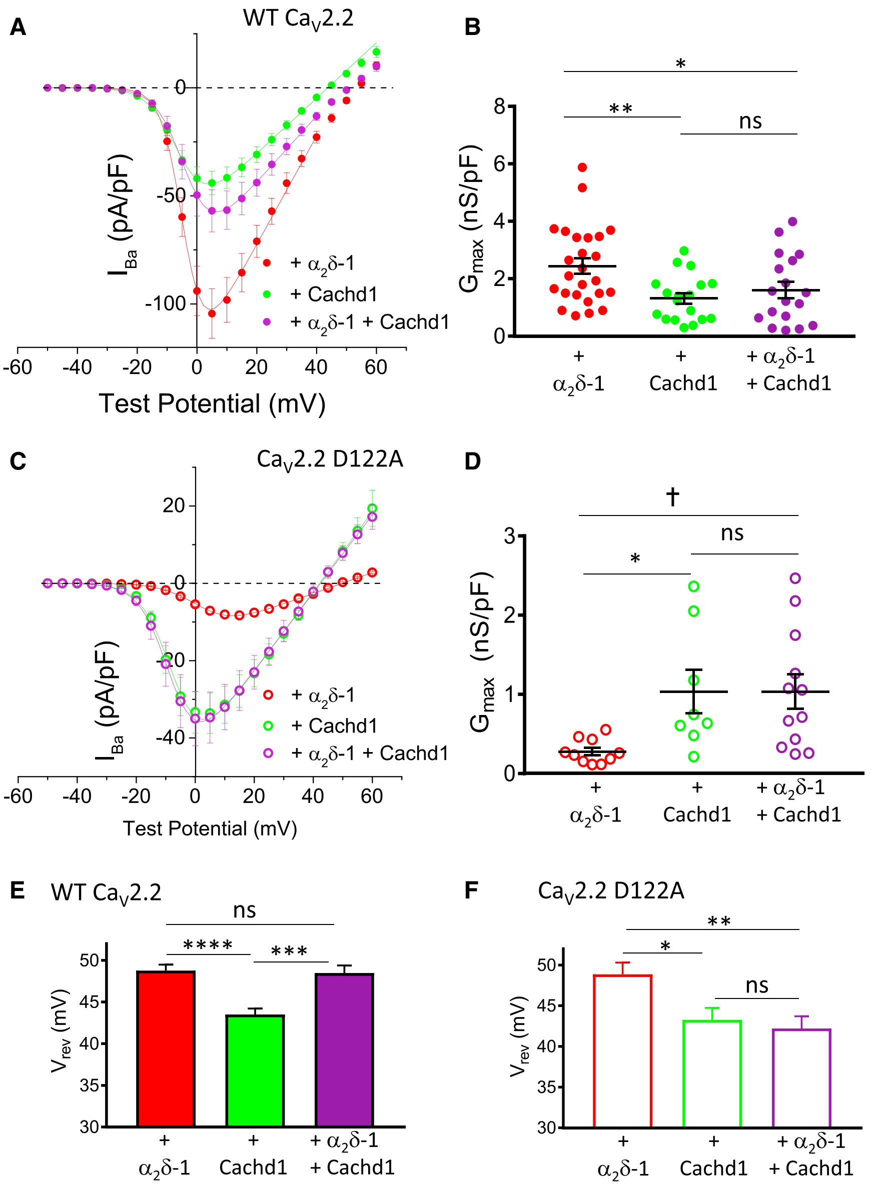

Figure 7. Cachd1 Competes with $\alpha_2\delta$-1 for Effects on $Ca_V2.2$ Currents

(A) Mean (± SEM) current-voltage relationships for WT $Ca_V2.2$-HA co-expressed with β1b and either $\alpha_2\delta$-1 (red solid circles, n = 25), Cachd1 (green solid circles, n = 18), or $\alpha_2\delta$-1 and Cachd1 (purple solid circles, n = 18). The individual and mean data were fit with a modified Boltzmann equation (STAR Methods).

(B) G_{max} (nS/pF) from the current-voltage relationships shown in (A). Individual data (same symbols as in A) and mean ± SEM are plotted. *p = 0.0483, **p = 0.0086 (1-way ANOVA with Holm-Sidak's *post hoc* test correcting for multiple comparisons).

(C) Mean (± SEM) current-voltage relationships for D122A $Ca_V2.2$-HA co-expressed with β1b and either $\alpha_2\delta$-1 (red open circles, n = 10), Cachd1 (green open circles, n = 8), or $\alpha_2\delta$-1 and Cachd1 (purple open circles, n = 12).

(D) G_{max} (nS/pF) from the current-voltage relationships shown in (C). Individual data (same symbols as in C) and mean ± SEM are plotted. *p = 0.0342, †p = 0.0265 (1-way ANOVA with Holm-Sidak's *post hoc* test correcting for multiple comparisons).

(E and F) Bar charts of mean (± SEM) V_{rev} (millivolt) for the conditions shown in (A) and (C), respectively. *p = 0.0396, **p = 0.0088, ***p = 0.0002, ****p < 0.0001 (1-way ANOVA with Holm-Sidak's *post hoc* test correcting for multiple comparisons).

subunits, neither the trafficking effects of Cachd1 in N2A cells nor its effect on calcium channel currents were affected by the presence of the D122A mutation in $Ca_V2.2$. Therefore, these effects of Cachd1 are likely not to be mediated via its disrupted MIDAS motif but, rather, due to interactions of the Cache or other domains in the protein. Interestingly, our results indicate that the effect of Cachd1 on cell surface expression of $Ca_V2.2$ involves a reduction in $Ca_V2.2$ endocytosis. It is highly unlikely that this is a non-specific effect because we have previously provided many examples of protein constructs that do not increase $Ca_V2.2$ currents or cell surface expression (Ferron et al., 2008; Kadurin et al., 2016, 2017; Macabuag and Dolphin, 2015).

In $\alpha_2\delta$-1 and $\alpha_2\delta$-2, the key MIDAS motif in the VWA domain contains three polar or negatively charged residues and has the sequence DVSGS. It is these three residues (D259, S261, and S263 in rat $\alpha_2\delta$-1 used here), plus two others (T331 and D363) in separate loops of the VWA domain that, together with the VWA protein ligand ($Ca_V2.2$ in this study), coordinate a divalent cation. In $\alpha_2\delta$-3 and $\alpha_2\delta$-4, one of these other coordinating residues is non-polar, but the MIDAS motif is intact. We confirm here that the increase in $Ca_V2.2$ currents caused by $\alpha_2\delta$-3 is also abolished by the D122A mutation in $Ca_V2.2$. A similar result was found for the interaction of $Ca_V1.2$ with $\alpha_2\delta$-1 in an extensive site-directed mutagenesis study (Bourdin et al., 2017).

By contrast, Cachd1 contains a VWA domain that has a disrupted MIDAS motif (DHGAS), a sequence that is conserved in the human, rat, mouse, and zebrafish Cachd1 proteins. This conservation across species supports the possibility that it may retain some function. Indeed, the ability of Cachd1 to increase the trafficking of $Ca_V2.2$ into hippocampal neurites was significantly reduced for D122A $Ca_V2.2$, suggesting that the disrupted MIDAS motif in Cachd1 may play some role in the interaction required for $Ca_V2.2$ trafficking into neurites. This result also indicates that there may be more stringent trafficking requirements for this channel in neurons. We drew a similar conclusion in a previous study, in which we showed that immature pro-$\alpha_2\delta$-1 could traffic $Ca_V2.2$ to the cell surface in non-neuronal cells but not into hippocampal neurites, where

mature proteolytically processed $\alpha_2\delta$-1 was required (Kadurin et al., 2016).

We have shown previously that, when the three polar or charged residues of the $\alpha_2\delta$-1 MIDAS motif are mutated to alanine, $\alpha_2\delta$-1 still associates with $Ca_V2.2$, as judged by its ability to occlude antigenic epitopes within the Cache domains of $\alpha_2\delta$-1 (Cassidy et al., 2014), although it fails to promote $Ca_V2.2$ trafficking. This indicates that there are certainly additional interaction sites as well as the MIDAS site interaction between $\alpha_2\delta$-1 and $Ca_V2.2$. The cryo-EM structure of the skeletal muscle calcium channel complex indicates clearly that $\alpha_2\delta$-1 interacts with $Ca_V1.1$ via multiple sites in addition to the divalent cation-mediated VWA domain interaction, including an interaction of a Cache domain with the turret of pore loop 5 in domain III (Wu et al., 2016). Such an interaction with the pore domain of $Ca_V2.2$ could also potentially explain the effect of Cachd1 on the apparent reversal potential. Because Cachd1 was able to coIP $Ca_V2.2$, partially co-localized with $Ca_V2.2$ on the cell surface of transfected cells, and also affected the reversal potential of these channels, it is clear that Cachd1 is not solely a trafficking protein but influences functional channels in the plasma membrane. The influence of another protein on the reversal potential of a channel, interpreted as an effect on its selectivity filter, has been observed previously (Stephan et al., 2018). The lack of effect of Cachd1 on $Ca_V2.1$ currents may relate to a particular splice variant or be common to all isoforms of $Ca_V2.1$ and should allow us to localize the site of selective interaction of Cachd1 with $Ca_V2.2$ in the future.

The finding that Cachd1 was able to inhibit the co-IP between $Ca_V2.2$ and $\alpha_2\delta$-1 and reduce the effect of $\alpha_2\delta$-1 on $Ca_V2.2$ cell surface expression and $Ca_V2.2$ currents indicates that, *in vivo*, it could play either a positive or an inhibitory role on $Ca_V2.2$ currents, depending on the degree of association of the $Ca_V2.2$ channels with $\alpha_2\delta$. From mRNA expression screens, Cachd1 is widely expressed in many tissues, including the brain, lungs, and small intestine. Of particular interest here is that Cachd1 mRNA expression was highest in dorsal root ganglia of all mouse tissues examined (see the transcriptome database described in Ray et al., 2018), raising the intriguing possibility that Cachd1 may modulate the efficacy of $\alpha_2\delta$-1 following neuropathic injury, a hypothesis that we will investigate in future studies.

Within the brain, there is strong expression of $\alpha_2\delta$-1 mRNA in the mouse hippocampus (Schlick et al., 2010). It is expressed strongly in CA1 and also present in dentate granule neurons. Cachd1 mRNA is also expressed in the mouse hippocampus, and, within the pyramidal cell layer, it is particularly prominent in CA3 but also in CA1 (Allen Mouse Brain Atlas; mouse.brain-map.org/api/index.html). Thus, Cachd1 and $\alpha_2\delta$-1 are likely to be expressed in overlapping cell types in the hippocampus. Within the rat hippocampus, there is a robust signal for $\alpha_2\delta$-1 protein in synaptic regions, including the dentate gyrus molecular layer, the *stratum lucidum* of CA3, and the CA1 *stratum oriens* and *stratum radiatum* (Nieto-Rostro et al., 2014; Taylor and Garrido, 2008); however, there are no equivalent data available for Cachd1 because of the paucity of antibodies and lack of knockout control tissue. Furthermore, in a large-scale proteomic study of non-neuronal cell lines, several proteins interacting with Cachd1 have been described recently (Huttlin et al., 2017); this suggests other potential roles for this protein in non-excitable cells (Rutledge et al., 2017).

In the future, it will be of great interest to determine the effect of Cachd1 on native calcium channels and whether its expression is altered in conditions such as neuropathic injury of primary afferent neurons to further elucidate its physiological role and to understand whether it competes endogenously with $\alpha_2\delta$-1 or other $\alpha_2\delta$ subunits.

STAR★METHODS

Detailed methods are provided in the online version of this paper and include the following:

- KEY RESOURCES TABLE
- CONTACT FOR REAGENT AND RESOURCE SHARING
- EXPERIMENTAL MODEL AND SUBJECT DETAILS
  - Cell lines
  - Primary Hippocampal cultures
- METHOD DETAILS
  - Molecular biology and constructs
  - Antibodies and other materials
  - Cell line transfection
  - Neuronal transfection
  - Cell surface biotinylation, cell lysis, deglycosylation and immunoblotting
  - Co–Immunoprecipitation
  - Immunocytochemistry
  - Endocytosis assay
  - Image Analysis
  - Electrophysiology
- QUANTIFICATION AND STATISTICAL ANALYSIS

SUPPLEMENTAL INFORMATION

Supplemental Information includes four figures and can be found with this article online at https://doi.org/10.1016/j.celrep.2018.10.033.

ACKNOWLEDGMENTS

This work was supported by Wellcome Trust investigator awards (098360/Z/12/Z to A.C.D. and 104682/Z/14/Z to S.W.). We thank Kanchan Chaggar for tissue culture of N2A and tsA-201 cells, Alistair Mathie for TASK3 cDNA, Heather Stickney (S.W.W. lab) for the zebrafish *Cachd1* cDNA, and Gavin J. Wright (Wellcome Trust Sanger Institute) for facilities for protein production and Ab purification. S.W.W. and G.T.P. thank Ana Faro and other lab members for insightful discussions regarding Cachd1.

AUTHOR CONTRIBUTIONS

D.Y.H. and W.S.P. made cDNA constructs. G.T.P. made the Cachd1 Ab. S.D. performed all electrophysiology. K.M.P. and D.Y.H. performed imaging studies and analyses for studies using D122A $Ca_V2.2$. S.D. and K.M.P. performed imaging and analyses on Cachd1. I.K. performed all biochemistry. L.F. developed hippocampal culture and transfection methods and performed endocytosis experiments. A.C.D. conceived the study together with S.W.W. and G.T.P., who conceived the study of Cachd1. A.C.D. wrote the manuscript, aided by all other authors.

DECLARATION OF INTERESTS

The authors declare no competing financial interests.

Received: April 12, 2018
Revised: August 31, 2018
Accepted: October 5, 2018
Published: November 6, 2018

REFERENCES

Altier, C., Garcia-Caballero, A., Simms, B., You, H., Chen, L., Walcher, J., Tedford, H.W., Hermosilla, T., and Zamponi, G.W. (2011). The Cavβ subunit prevents RFP2-mediated ubiquitination and proteasomal degradation of L-type channels. Nat. Neurosci. *14*, 173–180.

Anantharaman, V., and Aravind, L. (2000). Cache - a signaling domain common to animal Ca(2+)-channel subunits and a class of prokaryotic chemotaxis receptors. Trends Biochem. Sci. *25*, 535–537.

Bourdin, B., Briot, J., Tétreault, M.P., Sauvé, R., and Parent, L. (2017). Negatively charged residues in the first extracellular loop of the L-type Ca_V1.2 channel anchor the interaction with the $Ca_V\alpha2\delta1$ auxiliary subunit. J. Biol. Chem. *292*, 17236–17249.

Brodbeck, J., Davies, A., Courtney, J.M., Meir, A., Balaguero, N., Canti, C., Moss, F.J., Page, K.M., Pratt, W.S., Hunt, S.P., et al. (2002). The ducky mutation in Cacna2d2 results in altered Purkinje cell morphology and is associated with the expression of a truncated alpha 2 delta-2 protein with abnormal function. J. Biol. Chem. *277*, 7684–7693.

Cantí, C., Nieto-Rostro, M., Foucault, I., Heblich, F., Wratten, J., Richards, M.W., Hendrich, J., Douglas, L., Page, K.M., Davies, A., and Dolphin, A.C. (2005). The metal-ion-dependent adhesion site in the Von Willebrand factor-A domain of alpha2delta subunits is key to trafficking voltage-gated Ca2+ channels. Proc. Natl. Acad. Sci. USA *102*, 11230–11235.

Cassidy, J.S., Ferron, L., Kadurin, I., Pratt, W.S., and Dolphin, A.C. (2014). Functional exofacially tagged N-type calcium channels elucidate the interaction with auxiliary α2δ-1 subunits. Proc. Natl. Acad. Sci. USA *111*, 8979–8984.

Davies, A., Kadurin, I., Alvarez-Laviada, A., Douglas, L., Nieto-Rostro, M., Bauer, C.S., Pratt, W.S., and Dolphin, A.C. (2010). The α2δ subunits of voltage-gated calcium channels form GPI-anchored proteins, a posttranslational modification essential for function. Proc. Natl. Acad. Sci. USA *107*, 1654–1659.

De Jongh, K.S., Warner, C., and Catterall, W.A. (1990). Subunits of purified calcium channels. α 2 and δ are encoded by the same gene. J. Biol. Chem. *265*, 14738–14741.

Durocher, Y., Perret, S., and Kamen, A. (2002). High-level and high-throughput recombinant protein production by transient transfection of suspension-growing human 293-EBNA1 cells. Nucleic Acids Res. *30*, E9.

Ellis, S.B., Williams, M.E., Ways, N.R., Brenner, R., Sharp, A.H., Leung, A.T., Campbell, K.P., McKenna, E., Koch, W.J., Hui, A., et al. (1988). Sequence and expression of mRNAs encoding the α_1 and α_2 subunits of a DHP-sensitive calcium channel. Science *241*, 1661–1664.

Ferron, L., Davies, A., Page, K.M., Cox, D.J., Leroy, J., Waithe, D., Butcher, A.J., Sellaturay, P., Bolsover, S., Pratt, W.S., et al. (2008). The stargazin-related protein gamma 7 interacts with the mRNA-binding protein heterogeneous nuclear ribonucleoprotein A2 and regulates the stability of specific mRNAs, including CaV2.2. J. Neurosci. *28*, 10604–10617.

Ferron, L., Kadurin, I., and Dolphin, A.C. (2018). Proteolytic maturation of $\alpha_2\delta$ controls the probability of synaptic vesicular release. eLife *7*, e37507.

Field, M.J., Cox, P.J., Stott, E., Melrose, H., Offord, J., Su, T.Z., Bramwell, S., Corradini, L., England, S., Winks, J., et al. (2006). Identification of the α2-δ-1 subunit of voltage-dependent calcium channels as a molecular target for pain mediating the analgesic actions of pregabalin. Proc. Natl. Acad. Sci. USA *103*, 17537–17542.

Gurnett, C.A., De Waard, M., and Campbell, K.P. (1996). Dual function of the voltage-dependent Ca^{2+} channel $\alpha_2\delta$ subunit in current stimulation and subunit interaction. Neuron *16*, 431–440.

Gurnett, C.A., Felix, R., and Campbell, K.P. (1997). Extracellular interaction of the voltage-dependent Ca^{2+} channel $\alpha_2\delta$ and α_1 subunits. J. Biol. Chem. *272*, 18508–18512.

Hoppa, M.B., Lana, B., Margas, W., Dolphin, A.C., and Ryan, T.A. (2012). α2δ expression sets presynaptic calcium channel abundance and release probability. Nature *486*, 122–125.

Huttlin, E.L., Bruckner, R.J., Paulo, J.A., Cannon, J.R., Ting, L., Baltier, K., Colby, G., Gebreab, F., Gygi, M.P., Parzen, H., et al. (2017). Architecture of the human interactome defines protein communities and disease networks. Nature *545*, 505–509.

Jay, S.D., Sharp, A.H., Kahl, S.D., Vedvick, T.S., Harpold, M.M., and Campbell, K.P. (1991). Structural characterization of the dihydropyridine-sensitive calcium channel α_2-subunit and the associated δ peptides. J. Biol. Chem. *266*, 3287–3293.

Kadurin, I., Alvarez-Laviada, A., Ng, S.F., Walker-Gray, R., D'Arco, M., Fadel, M.G., Pratt, W.S., and Dolphin, A.C. (2012). Calcium currents are enhanced by α2δ-1 lacking its membrane anchor. J. Biol. Chem. *287*, 33554–33566.

Kadurin, I., Ferron, L., Rothwell, S.W., Meyer, J.O., Douglas, L.R., Bauer, C.S., Lana, B., Margas, W., Alexopoulos, O., Nieto-Rostro, M., et al. (2016). Proteolytic maturation of $\alpha_2\delta$ represents a checkpoint for activation and neuronal trafficking of latent calcium channels. eLife *5*, e21143.

Kadurin, I., Rothwell, S.W., Lana, B., Nieto-Rostro, M., and Dolphin, A.C. (2017). LRP1 influences trafficking of N-type calcium channels via interaction with the auxiliary $\alpha_2\delta$-1 subunit. Sci. Rep. *7*, 43802.

Kim, M.-S., Morii, T., Sun, L.-X., Imoto, K., and Mori, Y. (1993). Structural determinants of ion selectivity in brain calcium channel. FEBS Lett. *318*, 145–148.

Klugbauer, N., Lacinová, L., Marais, E., Hobom, M., and Hofmann, F. (1999). Molecular diversity of the calcium channel α2δ subunit. J. Neurosci. *19*, 684–691.

Leung, A.T., Imagawa, T., and Campbell, K.P. (1987). Structural characterization of the 1,4-dihydropyridine receptor of the voltage-dependent Ca2+ channel from rabbit skeletal muscle. Evidence for two distinct high molecular weight subunits. J. Biol. Chem. *262*, 7943–7946.

Macabuag, N., and Dolphin, A.C. (2015). Alternative splicing in Ca(V)2.2 regulates neuronal trafficking via adaptor protein complex-1 adaptor protein motifs. J. Neurosci. *35*, 14636–14652.

McGivern, J.G., and McDonough, S.I. (2004). Voltage-gated calcium channels as targets for the treatment of chronic pain. Curr. Drug Targets CNS Neurol. Disord. *3*, 457–478.

Morales, M., Colicos, M.A., and Goda, Y. (2000). Actin-dependent regulation of neurotransmitter release at central synapses. Neuron *27*, 539–550.

Nanou, E., and Catterall, W.A. (2018). Calcium Channels, Synaptic Plasticity, and Neuropsychiatric Disease. Neuron *98*, 466–481.

Nieto-Rostro, M., Sandhu, G., Bauer, C.S., Jiruska, P., Jefferys, J.G., and Dolphin, A.C. (2014). Altered expression of the voltage-gated calcium channel subunit $\alpha_2\delta$-1: a comparison between two experimental models of epilepsy and a sensory nerve ligation model of neuropathic pain. Neuroscience *283*, 124–137.

Page, K.M., Rothwell, S.W., and Dolphin, A.C. (2016). The CaVβ subunit protects the I-II loop of the voltage-gated calcium channel CaV2.2, from proteasomal degradation but not oligoubiquitination. J. Biol. Chem. *291*, 20402–20416.

Pragnell, M., Sakamoto, J., Jay, S.D., and Campbell, K.P. (1991). Cloning and tissue-specific expression of the brain calcium channel β-subunit. FEBS Lett. *291*, 253–258.

Pragnell, M., De Waard, M., Mori, Y., Tanabe, T., Snutch, T.P., and Campbell, K.P. (1994). Calcium channel β-subunit binds to a conserved motif in the I-II cytoplasmic linker of the α_1-subunit. Nature *368*, 67–70.

Raghib, A., Bertaso, F., Davies, A., Page, K.M., Meir, A., Bogdanov, Y., and Dolphin, A.C. (2001). Dominant-negative synthesis suppression of voltage-gated calcium channel Ca_v2.2 induced by truncated constructs. J. Neurosci. *21*, 8495–8504.

Ray, P., Torck, A., Quigley, L., Wangzhou, A., Neiman, M., Rao, C., Lam, T., Kim, J.Y., Kim, T.H., Zhang, M.Q., et al. (2018). Comparative transcriptome profiling of the human and mouse dorsal root ganglia: an RNA-seq-based resource for pain and sensory neuroscience research. Pain *159*, 1325–1345.

Rutledge, E.A., Benazet, J.D., and McMahon, A.P. (2017). Cellular heterogeneity in the ureteric progenitor niche and distinct profiles of branching morphogenesis in organ development. Development *144*, 3177–3188.

Savalli, N., Pantazis, A., Sigg, D., Weiss, J.N., Neely, A., and Olcese, R. (2016). The α2δ-1 subunit remodels CaV1.2 voltage sensors and allows Ca2+ influx at physiological membrane potentials. J. Gen. Physiol. *148*, 147–159.

Schlick, B., Flucher, B.E., and Obermair, G.J. (2010). Voltage-activated calcium channel expression profiles in mouse brain and cultured hippocampal neurons. Neuroscience *167*, 786–798.

Shaner, N.C., Campbell, R.E., Steinbach, P.A., Giepmans, B.N., Palmer, A.E., and Tsien, R.Y. (2004). Improved monomeric red, orange and yellow fluorescent proteins derived from Discosoma sp. red fluorescent protein. Nat. Biotechnol. *22*, 1567–1572.

Shy, D., Gillet, L., Ogrodnik, J., Albesa, M., Verkerk, A.O., Wolswinkel, R., Rougier, J.S., Barc, J., Essers, M.C., Syam, N., et al. (2014). PDZ domain-binding motif regulates cardiomyocyte compartment-specific NaV1.5 channel expression and function. Circulation *130*, 147–160.

Soubrane, C.H., Stevens, E.B., and Stephens, G.J. (2012). Expression and functional studies of the novel CNS protein CACHD1. Proc. Phys. Soc. *27*, PC74.

Stea, A., Dubel, S.J., Pragnell, M., Leonard, J.P., Campbell, K.P., and Snutch, T.P. (1993). A β-subunit normalizes the electrophysiological properties of a cloned N-type Ca^{2+} channel α_1-subunit. Neuropharmacology *32*, 1103–1116.

Stephan, G., Huang, L., Tang, Y., Vilotti, S., Fabbretti, E., Yu, Y., Nörenberg, W., Franke, H., Gölöncsér, F., Sperlágh, B., et al. (2018). The ASIC3/P2X3 cognate receptor is a pain-relevant and ligand-gated cationic channel. Nat. Commun. *9*, 1354.

Takahashi, M., Seagar, M.J., Jones, J.F., Reber, B.F.X., and Catterall, W.A. (1987). Subunit structure of dihydropyridine-sensitive calcium channels from skeletal muscle. Proc. Natl. Acad. Sci. USA *84*, 5478–5482.

Taylor, C.P., and Garrido, R. (2008). Immunostaining of rat brain, spinal cord, sensory neurons and skeletal muscle for calcium channel alpha2-delta (alpha2-delta) type 1 protein. Neuroscience *155*, 510–521.

Van Petegem, F., Clark, K.A., Chatelain, F.C., and Minor, D.L., Jr. (2004). Structure of a complex between a voltage-gated calcium channel beta-subunit and an alpha-subunit domain. Nature *429*, 671–675.

Waithe, D., Ferron, L., Page, K.M., Chaggar, K., and Dolphin, A.C. (2011). β-subunits promote the expression of Ca(V)2.2 channels by reducing their proteasomal degradation. J. Biol. Chem. *286*, 9598–9611.

Whittaker, C.A., and Hynes, R.O. (2002). Distribution and evolution of von Willebrand/integrin A domains: widely dispersed domains with roles in cell adhesion and elsewhere. Mol. Biol. Cell *13*, 3369–3387.

Wu, J., Yan, Z., Li, Z., Qian, X., Lu, S., Dong, M., Zhou, Q., and Yan, N. (2016). Structure of the voltage-gated calcium channel Ca(v)1.1 at 3.6 Å resolution. Nature *537*, 191–196.

Zamponi, G.W., Striessnig, J., Koschak, A., and Dolphin, A.C. (2015). The Physiology, Pathology, and Pharmacology of Voltage-Gated Calcium Channels and Their Future Therapeutic Potential. Pharmacol. Rev. *67*, 821–870.

STAR★METHODS

KEY RESOURCES TABLE

REAGENT or RESOURCE	SOURCE	IDENTIFIER
Antibodies		
$\alpha_2\delta$-1 Ab	Sigma-Aldrich	Cat # C5105; RRID:AB_258885
Anti-Ca_V2.2 II-III loop Ab (rabbit polyclonal)	(Raghib et al., 2001)	n/a
Anti-HA Ab rat monoclonal	Sigma-Aldrich	Cat# 11815016001; RRID:AB_390914
Anti-HA Ab rabbit	Sigma-Aldrich	Cat # H6908; RRID:AB_260070
Anti-GAPDH Ab	Ambion	Cat # AM4300; RRID:AB_2536381
Anti-GFP Ab (Living Colors, rabbit polyclonal)	Takara Bio Clontech	Cat # 632375
Anti-rabbit Alexa fluor 594	Thermo Fisher	Cat # R37117; RRID:AB_2556545
Anti-rat Alexa fluor 488	Thermo Fisher	Cat # A-11006; RRID:AB_2534074
Anti-mouse Alexa fluor 647	Thermo Fisher	Cat # A32728; RRID:AB_2633277
Anti-rat fluorescein isothiocyanate	Sigma-Aldrich	Cat # F1763; RRID:AB_259443
Goat anti-rabbit HRP	Biorad	Cat # 1706515; RRID:AB_11125142
Goat anti-rat HRP	Biorad	Cat # 5204-2504; RRID:AB_619913
Goat anti-mouse HRP	Biorad,	Cat # 1721011; RRID:AB_11125936
Affinity-purified Cachd1 rabbit polyclonal Ab	G. T. Powell and S.W Wilson, UCL.	n/a
Chemicals, Peptides, and Recombinant Proteins		
ω-conotoxin GVIA	Alomone	Cat # C-300
Penicillin-Streptomycin (10,000 U/mL)	Invitrogen	Cat # 15140-122
Poly-L-lysine	Sigma-Aldrich	Cat # P.6282
Dulbecco's modified Eagle's medium	Thermo Fisher	Cat #4 1965-039
GlutaMAX	Invitrogen	Cat # 35050-038
Fugene	Promega	Cat # E2311
Polyjet	Tebu-bio Ltd	Cat # 189-SL100688-1
Opti-MEM	Thermo Fisher	Cat # 41965-039
Neurobasal Medium	Invitrogen	Cat # 10888-022
B27	Thermo Fisher	Cat # 17504044
HEPES	Sigma-Aldrich	Cat # H3375
Horse serum	Invitrogen	Cat # 26050-088
Lipofectamine 2000	Invitrogen	Cat # L3000-008
Premium Grade EZ-link Sulfo-NHS-LC-Biotin	Thermo Fisher	Cat # 21335
Glycine	Sigma-Aldrich	Cat # G8898
SDS	VWR	Cat # 444062F
Protease Inhibitors	Roche	Cat # 11697498001
DTT	Melford	Cat # MB1015
SDS-polyacrylamide gel electrophoresis	Invitrogen	Cat # EA0375BOX
polyvinylidene fluoride (PVDF) membrane	Biorad	Cat # 1620177
streptavidin-agarose beads	Thermo Fisher	Cat # 20347
Igepal	Sigma-Aldrich	Cat # I3021
PNGase-F	Roche Applied Science	Cat # 11365177001
Digitonin	Millipore	Cat # 300410
A/G PLUS Agarose slurry Santa Cruz	Santa Cruz	Cat # Sc-2003
Paraformaldehyde	Sigma-Aldrich	Cat # P6148
Goat serum	Invitrogen	Cat # 6210-072
Triton X-100	Thermo Fisher	Cat # 28314

(*Continued on next page*)

Continued

REAGENT or RESOURCE	SOURCE	IDENTIFIER
4',6-diamidine-2′-phenylindole dihydrochloride (DAPI)	Molecular probes	Cat # nl5995050
VectaShield	Vector Laboratories	Cat # H1000
papain	Sigma-Aldrich	Cat # P4762
L-cysteine	Sigma-Aldrich	Cat # c7755
bovine serum albumin	First Link UK ltd	Cat # 41-00-410
DNase	Sigma-Aldrich	Cat # D5025
Hank's basal salt solution	Thermo Fisher	Cat # 14175-053
α-bungarotoxin Alexa Fluor® 488 conjugate (BTX488)	Thermo Fisher	Cat # B13422
fetal bovine serum	Invitrogen	Cat # 10270
Critical Commercial Assays		
Bradford Assay	Biorad	Cat # 500-0006
ECL 2	Thermo Fisher	Cat # 32132
Experimental Models: Cell Lines		
tsA-201 cells	ECACC	Cat # 96121229
Neuro2A cells	ATCC	CCL-131
Experimental Models: Organisms/Strains		
Rat Sprague Dawley male	UCL bred in house	n/a
Oligonucleotides		
Primer for introducing the D122A mutation. reverse 5′-GACATAGGCGTCTTGGCCCCGTCAG-3′	this paper	n/a
Primer for introducing the D122A mutation forward 5′-CTGACGGGGCCAAGACGCCTATGTC-3′	this paper	n/a
Recombinant DNA		
Rabbit $Ca_V2.2$ HA	(Cassidy et al., 2014)	n/a
Rat β1b (X61394)	(Pragnell et al., 1991)	n/a
Rat $\alpha_2\delta$-1 (M86621)	(Kim et al., 1993)	n/a
HA tagged $\alpha_2\delta$-1	(Kadurin et al., 2012)	n/a
Rat $Ca_V2.1$	(Brodbeck et al., 2002)	n/a
Human TASK3 (KCNK9) (NM_001282534)	obtained from Prof. A Mathie	n/a
Zebrafish zCachd1	G. T. Powell and S.W Wilson, UCL.	n/a
Rat rCachd1	OriGene	Cat # RN217577
GFP_$Ca_V2.2$-HA	(Macabuag and Dolphin, 2015)	n/a
$Ca_V2.2$-BBS	(Cassidy et al., 2014)	n/a
$Ca_V2.2$-HA D122A	This paper	n/a
GFP_$Ca_V2.2$-HA D122A	This paper	n/a
rCachd1_GFP	This paper	n/a
zCachd1_GFP	G. T. Powell and S.W Wilson, UCL.	n/a
Mouse $\alpha_2\delta$-3 (AJ010949)	(Klugbauer et al., 1999)	n/a
mcherry (AY678264)	(Shaner et al., 2004)	n/a
CD8	(Shy et al., 2014)	n/a
Software and Algorithms		
ImageJ	National Institutes of Health https://imagej.nih.gov/ij/	RRID:SCR_003070
GraphPad Prism 5 or 7	https://www.graphpad.com	n/a
Origin-Pro 2015	Microcal Origin, Northampton, MA	n/a
pCLAMP 9	Molecular Devices	n/a

CONTACT FOR REAGENT AND RESOURCE SHARING

Further information and requests for resources and reagents should be directed to and will be fulfilled where possible by the Lead Contact, Annette Dolphin (a.dolphin@ucl.ac.uk).

EXPERIMENTAL MODEL AND SUBJECT DETAILS

Cell lines

Cell lines were plated onto cell culture flasks, coverslips coated with poly-L-lysine, and cultured in a 5% CO_2 incubator at 37°C. tsA-201 cells (ECACC, female sex) were cultured in Dulbecco's modified Eagle's medium in the presence of 10% fetal bovine serum, penicillin, streptomycin and 2% GlutaMAX (Invitrogen). N2A cells (ATCC, male sex) used for immunocytochemistry experiments, were cultured in DMEM and OPTI-MEM (1:1), supplemented with FBS (5%), penicillin (1 unit/ml), streptomycin (1 μg/ml), and GlutaMAX (1%).

Primary Hippocampal cultures

Hippocampal neurons were obtained from P0 rat pups (Sprague-Dawley, male), as previously described (Morales et al., 2000). All experiments were performed in accordance with the UK Home Office Animals (Scientific procedures) Act 1986, using a Schedule 1 method, with UCL ethical approval. Briefly, hippocampi were dissected and treated for 40 min at 37°C with a papain solution containing: 70 units /ml of papain, 0.2 mg/ml L-cysteine, 0.2 mg/ml bovine serum albumin (BSA), 1 mg/ml DNase and 5mg/ml glucose (all from Sigma Aldrich) in Hank's basal salt solution (HBSS) medium (Invitrogen). Hippocampi were then washed twice with plating solution (Neurobasal medium supplemented with B27 (Thermo Fisher Scientific; 2%), HEPES (10 mM), horse serum (5%), glutamine (0.5 mM) and 1 unit/ml penicillin, 1 μg/ml streptomycin), and the neurons were mechanically dissociated using fire-polished glass Pasteur pipettes with decreasing diameter. Approximately 75×10^3 cells in 100 μl of plating solution were seeded onto sterile poly-lysine-coated glass coverslips. After 2 h, the plating solution was replaced with 1 ml of growth medium (serum-free Neurobasal medium supplemented with B27 (4%), 2-mercaptoethanol (25 μM), glutamine (0.5 mM), and 1 unit/ml penicillin, 1 μg/ml streptomycin), half of which was replaced every 3-4 days. At 7 days *in vitro* and 2 h before transfection, half of the medium was removed, and kept as 'conditioned' medium, and 500 μl of fresh medium was added.

METHOD DETAILS

Molecular biology and constructs

cDNAs encoding the following proteins were used: calcium channel $Ca_V2.2$ (rabbit, GenBank: D14157), containing an extracellular HA tag (Cassidy et al., 2014), β1b (rat, GenBank: X61394), $\alpha_2\delta$-1 (rat, GenBank: M86621), HA-tagged $\alpha_2\delta$-1 (Kadurin et al., 2012), rat $Ca_v2.1$ (GenBank: M64373), human TASK3 (KCNK9) cDNA (GenBank: NM_001282534) and mCherry. Zebrafish zCachd1 was cloned from a zebrafish cDNA library. Rat rCachd1 cDNA (GenBank: NM_001191758) was purchased from OriGene. Note that the *Cachd1* gene was misnamed *Cacna2d4* in the original bioinformatics paper in which it was identified as $\alpha_2\delta$-like (Whittaker and Hynes, 2002). All cDNAs were subcloned into the expression vectors pMT2, pcDNA3 and pCAGGS. In some experiments, $Ca_V2.2$-HA also had the green fluorescent protein, mut3bGFP (GFP), fused to the N terminus (Macabuag and Dolphin, 2015). The D122A mutation was introduced into $Ca_V2.2$ by mutating aspartate at position 122 of rabbit $Ca_V2.2$ to alanine by PCR. C-terminal GFP fusion proteins of both zCachd1 and rCachd1 were made by standard techniques, and used where stated. The sequences of all constructs were confirmed by DNA sequencing.

Antibodies and other materials

Ca channel Abs used were: $\alpha_2\delta$-1 Ab (mouse monoclonal against α_2-1 moiety, Sigma-Aldrich, epitope identified in (Cassidy et al., 2014)), anti-$Ca_V2.2$ II-III loop Ab (rabbit polyclonal) (Raghib et al., 2001). A bespoke, affinity-purified Cachd1 rabbit polyclonal Ab was raised by Cambridge Research Biochemicals (Billingham, UK) against the predicted extracellular domain of zCachd1 protein, produced by transient transfection of mammalian cells (Durocher et al., 2002) (G.T.P., S.W.W., and Gavin J. Wright, unpublished data). Purified Ab activity was confirmed by enzyme-linked immunosorbent assay. Other Abs used were anti-HA (rat monoclonal, Roche), anti-HA (rabbit polyclonal, Sigma), anti-GAPDH Ab (mouse monoclonal, Ambion), and GFP Ab (Living Colors, rabbit polyclonal; BD Biosciences). For immunocytochemistry, secondary Abs (1:500) used were anti-rabbit-Alexa Fluor 594, anti-rat-Alexa Fluor 488, anti-mouse-Alexa Fluor 647 (Life Technologies) or anti-rat fluorescein isothiocyanate (Sigma-Aldrich). The secondary Abs used for Western Blotting were goat anti-rabbit, goat anti-rat, and goat-anti-mouse Abs coupled to horseradish peroxidase (HRP) (Biorad). ω-conotoxin GVIA was purchased from Alomone, and applied by local perfusion.

Cell line transfection

For co-IPs and electrophysiological studies, tsA-201 cells were transfected using Fugene6 (Promega, Fitchburg, WI) according to the manufacturer's protocol. For immuno-cytochemistry, tsA-201 cells were transfected using PolyJet (SignaGen) according to the manufacturer's protocol. N2A cells were re-plated onto poly-lysine coated coverslips and transfections were carried out using PolyJet

(SignaGen) at a ratio of 3:1 to DNA mix according to manufacturer's instructions. For all electrophysiology and imaging experiments transfections, the cDNA mix consisted of cDNAs encoding WT or D122A $Ca_V2.2$, β1b, $\alpha_2\delta$-1 in a ratio of 3:2:2. The $\alpha_2\delta$-1 was replaced with Cachd1 or empty vector where appropriate. When these experiments involved both $\alpha_2\delta$-1 and Cachd1, $Ca_V2.2$, β1b, $\alpha_2\delta$-1 and Cachd1 were added in a ratio of 3:2:2:2, with empty vector replacing $\alpha_2\delta$-1 or Cachd1 where appropriate. For co-IP experiments $Ca_V2.2$ (with or without GFP and HA tags, as stated), β1b and $\alpha_2\delta$-1 were transfected in a ratio of 2:1:2. For co-IP competition experiments the transfection mix contained $Ca_V2.2$: β1b: $\alpha_2\delta$-1: (TASK3, Cachd1 or a 1:1 mix of both) in a ratio of 2:1:2:1. For reverse co-IP experiments, Cachd1_GFP, β1b, and $Ca_V2.2$ were transfected in a ratio of 2:1:2.

Neuronal transfection

The hippocampal cultures were then transfected using Lipofectamine 2000, at a ratio of 1:2 to DNA mix (1 μg/μl). After 2 h, the transfection mixes were replaced with growth medium consisting of 50% conditioned and 50% fresh medium. The DNA mix consisted of cDNAs in pCAGGS encoding WT $Ca_V2.2$ or D122A $Ca_V2.2$, $\alpha_2\delta$-1, β1b and mCherry, at a ratio of 3:2:2:0.5. $\alpha_2\delta$-1 was replaced by empty vector or rCachd1 when appropriate.

Cell surface biotinylation, cell lysis, deglycosylation and immunoblotting

The procedures were modified from those described in more detail previously (Kadurin et al., 2012; Kadurin et al., 2016). Briefly, 72 h after transfection, tsA-201 cells were incubated for 30 min at room temperature with 0.5 mg/ml Premium Grade EZ-link Sulfo-NHS-LC-Biotin (Thermo Scientific) in PBS and the reaction was quenched with 200 mM glycine. The cells were resuspended in PBS, pH 7.4 at 4°C containing 1% Igepal; 0.1% SDS and protease inhibitors (PI, cOmplete, Roche), to allow cell lysis, cleared by centrifugation at 18,000 × g and assayed for total protein (Bradford assay, Biorad). Cleared WCL corresponding to 20 – 40 μg total protein was mixed with Laemmli sample buffer (Davies et al., 2010) supplemented with 100 mM dithiothreitol (DTT), resolved by SDS-polyacrylamide gel electrophoresis (PAGE) on 3%–8% Tris-Acetate (Invitrogen) and transferred to polyvinylidene fluoride (PVDF) membrane (Biorad). The proteins were revealed by immunoblotting performed with the corresponding Abs essentially as described previously (Kadurin et al., 2012). The signal was obtained by HRP reaction with fluorescent product (ECL 2; Thermo Scientific) and membranes were scanned on a Typhoon 9410 phosphorimager (GE Healthcare). Biotinylated lysates (equalized to between 0.5 and 1 mg/ml total protein concentration) were applied to 40 μl prewashed streptavidin-agarose beads (Thermo Scientific) and rotated overnight at 4°C. The beads were then washed 3 times with PBS containing 0.1% Igepal and, when required, the streptavidin beads were deglycosylated for 3 h at 37°C with 1 unit of PNGase F (Roche Applied Science). The samples containing precipitated cell surface protein fractions were then analyzed by immunoblotting with the indicated Abs as described previously (Kadurin et al., 2012).

Co–Immunoprecipitation

The protocol described below was adapted from a procedure described previously (Gurnett et al., 1997). A tsA-201 cell pellet derived from one confluent 75 cm^2 flask was resuspended in co-IP buffer (20 mM HEPES (pH 7.4), 300 mM NaCl, 1% Digitonin and PI), sonicated for 8 s at 20 kHz and rotated for 1 h at 4°C. The samples were then diluted with an equal volume of 20 mM HEPES (pH 7.4), 300 mM NaCl, 1 mM $CaCl_2$, 1 mM $MgCl_2$, with PI (to 0.5% final concentration of Digitonin), mixed by pipetting and centrifuged at 20,000 x g for 20 min. The supernatants were collected and assayed for total protein (Bradford assay; Biorad). 1 mg of total protein was adjusted to 2 mg/ml with co-IP buffer and incubated overnight at 4°C with anti-GFP polyclonal Ab (1:200; BD Biosciences). 30 μl A/G PLUS Agarose slurry (Santa Cruz) was added to each tube and further rotated for 2 h at 4°C. The beads were then washed three times with co-IP buffer containing 0.2% Digitonin. The beads were then resuspended in 2 x Laemmli buffer with 100 mM DTT and analyzed alongside equalized aliquots of the initial lysate prior to co-IP by SDS-PAGE and western blotting as described above. The reverse co-IP experiments between Cachd1_GFP and $Ca_V2.2$-HA were performed under identical conditions except that the NaCl concentration in the co-IP buffer was 150 mM, and the beads were washed two times in co-IP buffer containing 0.1% Digitonin.

Immunocytochemistry

Cells were fixed with 4% paraformaldehyde in phosphate-buffered saline (PBS, pH 7.4) for 5 min, incubated with blocking buffer (20% goat serum, 4% BSA in PBS) for 1 h at room temperature before being incubated with rat anti-HA (Roche) diluted 1:200 in 0.5x blocking buffer for 1 h at room temperature. When permeabilization was included, cells were permeabilized with 0.2% Triton X-100 for 5 min before being incubated with the second primary Ab, rabbit anti-$Ca_V2.2$ II-III loop (1:250), for 1 h at room temperature. For hippocampal neurons, primary Ab incubation was carried out at 4°C overnight. After washing, samples were incubated with secondary Abs, anti-rat Alexa Fluor 488, anti-rat Alexa Fluor 594 and anti-rabbit Alexa Fluor 488, at a dilution of 1:500 for 1 h at room temperature. 4',6-diamidine-2′-phenylindole dihydrochloride (DAPI) was used to visualize the nuclei. Coverslips were washed and mounted in VectaShield (Vector Laboratories).

Endocytosis assay

N2A cells were transfected with a $Ca_V2.2$ construct tagged with a double bungarotoxin binding site epitope ($Ca_V2.2$-BBS) (Cassidy et al., 2014), β1b and either empty vector, $\alpha_2\delta$-1 or rCachd1. After 40 h expression, cells were washed twice with Krebs-Ringer solution with HEPES (KRH) (in mM; 125 NaCl, 5 KCl, 1.1 $MgCl_2$, 1.2 KH_2PO_4, 2 $CaCl_2$, 6 Glucose, 25 HEPES, 1 $NaHCO_3$) and incubated with 10 μg/ml α-bungarotoxin Alexa Fluor® 488 conjugate (BTX-488) (Thermo Fisher Scientific) at 17°C for 30 min. The

unbound BTX-488 was removed by washing with KRH, and the labeled cells were returned to 37°C for the kinetic assay. Endocytosis was terminated by fixing the cells with cold 4% PFA-sucrose in PBS at the specified time. The cells were then permeabilized and intracellular $Ca_V2.2$ was labeled using the rabbit anti-$Ca_V2.2$ II-III loop Ab as described above.

Image Analysis

N2A and tsA-201 cell samples were viewed on an LSM 780 confocal microscope (Zeiss) using either 63x/1.4 or 40x/1.3 numerical aperture oil-immersion objective in 16-bit mode. The tile function (3x3 tiles, each tile consisting of 1024x1024 pixels) was used and every transfected cell within the image was analyzed to remove collection bias. Hippocampal neurons were viewed using a 20x objective (neuronal processes) or 63x objective (soma); individual neurons were selected on the basis of mCherry expression. Acquisition settings, chosen to ensure that images were not saturated, were kept constant for each experiment. Images are individual optical sections, unless otherwise stated.

Images were analyzed using ImageJ (National Institutes of Health). For N2A cells, the freehand line tool (5 pixels) was used to manually trace the plasma membrane to measure the mean intensity of cell-surface staining. Intracellular staining was measured using the freehand selection tool, excluding the nucleus and the plasma membrane. For hippocampal neurons, two concentric circles (100 and 150 μm diameter) were centered on the soma and the freehand line tool (3 pixels) was used to trace the neuronal processes between the circles, using the mCherry image as the template. The background fluorescence was measured in an area with no transfected cells and subtracted from the mean intensity.

Electrophysiology

Calcium channel currents in transfected tsA-201 cells were investigated by whole cell patch-clamp recording. The patch pipette solution contained in mM: Cs-aspartate, 140; EGTA, 5; $MgCl_2$, 2; $CaCl_2$, 0.1; K_2ATP, 2; HEPES, 10; pH 7.2, 310 mOsm with sucrose. The external solution for recording Ba^{2+} currents contained in mM: tetraethylammonium (TEA) Br, 160; KCl, 3; $NaHCO_3$, 1.0; $MgCl_2$, 1.0; HEPES, 10; glucose, 4; $BaCl_2$, 1, pH 7.4, 320 mOsm with sucrose. 1 mM extracellular Ba^{2+} was the charge carrier. Pipettes of resistance 2-4 MΩ were used. An Axopatch 1D or Axon 200B amplifier was used, and whole cell voltage-clamp recordings were sampled at 10 kHz frequency, filtered at 2 kHz and digitized at 1 kHz. 70%–80% series resistance compensation was applied, and all recorded currents were leak subtracted using P/8 protocol. Membrane potential was held at – 80 mV. Analysis was performed using pCLAMP 9 (Molecular Devices) and Origin 7 (Microcal Origin, Northampton, MA). *IV* relationships were fit by a modified Boltzmann equation as follows: $I = G_{max}*(V-V_{rev})/(1+exp(-(V-V_{50,\ act})/k))$ where I is the current density (in pA/pF), G_{max} is the maximum conductance (in nS/pF), V_{rev} is the apparent reversal potential, $V_{50,\ act}$ is the midpoint voltage for current activation, and k is the slope factor.

QUANTIFICATION AND STATISTICAL ANALYSIS

Data were analyzed with GraphPad Prism 7 (GraphPad software, San Diego, CA) or Origin-Pro 2015 (OriginLab Corporation, Northampton, MA, USA). All data are shown as mean ± SEM; “n” refers to number of cells or neurites, unless indicated otherwise, and is given in the figure legends, together with details of statistical tests used. Experiments where representative data are shown were repeated at least 3 times, unless otherwise stated. Graphpad Prism 7 was used for statistical analysis. Statistical significance between two groups was assessed by Student’s t test, as stated. One-way ANOVA and the stated post hoc analysis was used for comparison of means between three or more groups.

Cell Reports, Volume *25*

Supplemental Information

The $\alpha_2\delta$-like Protein Cachd1 Increases N-type Calcium Currents and Cell Surface Expression and Competes with $\alpha_2\delta$-1

Shehrazade Dahimene, Karen M. Page, Ivan Kadurin, Laurent Ferron, Dominique Y. Ho, Gareth T. Powell, Wendy S. Pratt, Stephen W. Wilson, and Annette C. Dolphin

SUPPLEMENTARY FIGURES

Figure S1 (relates to Figure 1): Cachd1 is a membrane protein and sequence of rat Cachd1 showing predicted N-glycosylation sites.

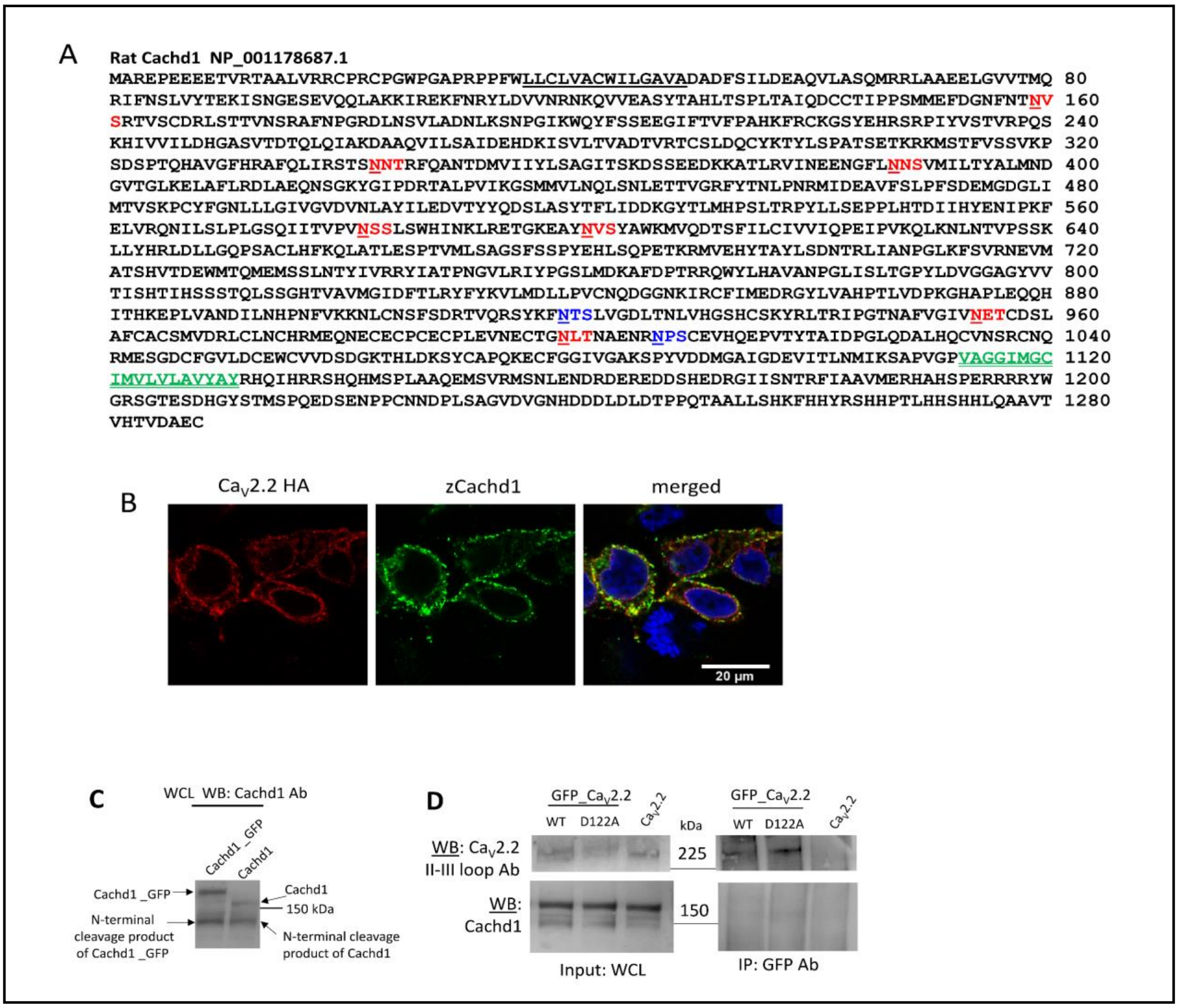

(A) Sequence of rCachd1. Highly predicted N-glycosylation NxS/T sequences are shown in red, sequences with a sub-threshold prediction level are in blue. The approximate predicted position of the N-terminal signal sequence is underlined, and the approximate predicted position of the transmembrane segment is underlined in green. Predictions are from Signal P4.1, NetNglyc 1.0 and ExPASy TMpred.
(B) Representative confocal images of tsA-201 cells expressing $Ca_V2.2$ HA WT (left) with β1b and zCachd1. Cells were permeabilized, and incubated with rat anti-HA and rabbit anti-Cachd1 Abs for 1 h to show distribution of HA staining (left panel, red) and Cachd1 (middle panel, green). Merged images (with co-localization in yellow) are shown in the right-hand panel; DAPI was used to stain the nuclei (blue). Scale bars are 20 µm.
(C) WCL input for experiment shown in Figure 1G, blotted with Cachd1 Ab. zCachd1_GFP (left lane) and untagged zCachd1 (right lane). The Cachd1 Ab shows two bands for both species, whereas the GFP Ab shows a single band for zCachd1_GFP (Figure 1G), indicating that the lower band (which is the same MW in both lanes) is a cleavage product containing the N-terminus of the protein.
(**D**) WCL input (left panels) and IP (right panels) for WT and D122A mutant GFP_$Ca_V2.2$, and untagged $Ca_V2.2$ control (upper panels), and for rCachd1 (lower panels). IP was performed with GFP Ab, and pulled down both WT and D122A GFP_$Ca_V2.2$ (upper right panel). Lack of co-IP of Cachd1 is shown in lower right panel. Representative of 3 experiments.

Figure S2 (relates to Figure 2): Effect of D122A mutation of $Ca_V2.2$ on $Ca_V2.2$ currents enhancement by $\alpha_2\delta$-3

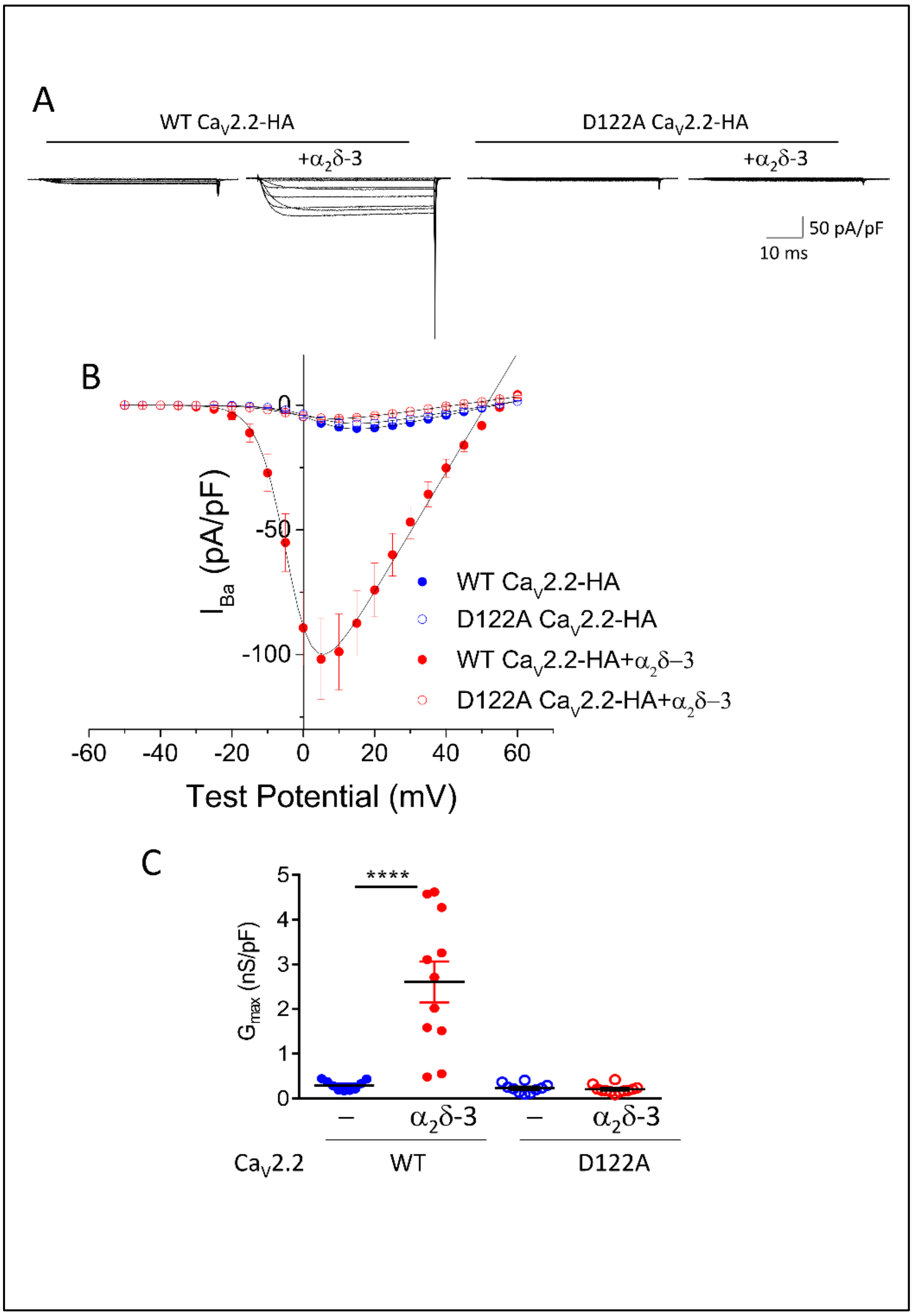

(A) Example families of $Ca_V2.2$ currents for WT $Ca_V2.2$-HA (left two) and D122A $Ca_V2.2$-HA (right two), co-expressed with β1b and either no $\alpha_2\delta$ (left), $\alpha_2\delta$-3 (right). Holding potential -80 mV, steps between -50 and +60 mV for 50 ms. Calibration bars apply to all traces.

(B) Mean (± SEM) current-voltage relationships for the conditions shown in (A). WT $Ca_V2.2$-HA (solid circles) and D122A $Ca_V2.2$-HA (open circles), co-expressed with β1b and either no $\alpha_2\delta$ (blue) or $\alpha_2\delta$-3 (red). The individual and mean data were fit with a modified Boltzmann equation (see Methods).

(C) G_{max} (nS/pF) from the current-voltage relationships shown in (B). Individual data (same symbols as B) and mean ± SEM are plotted. ****P<0.0001.

Figure S3 (relates to Figure 2G): Cachd1 does not increase $Ca_V2.1$ currents

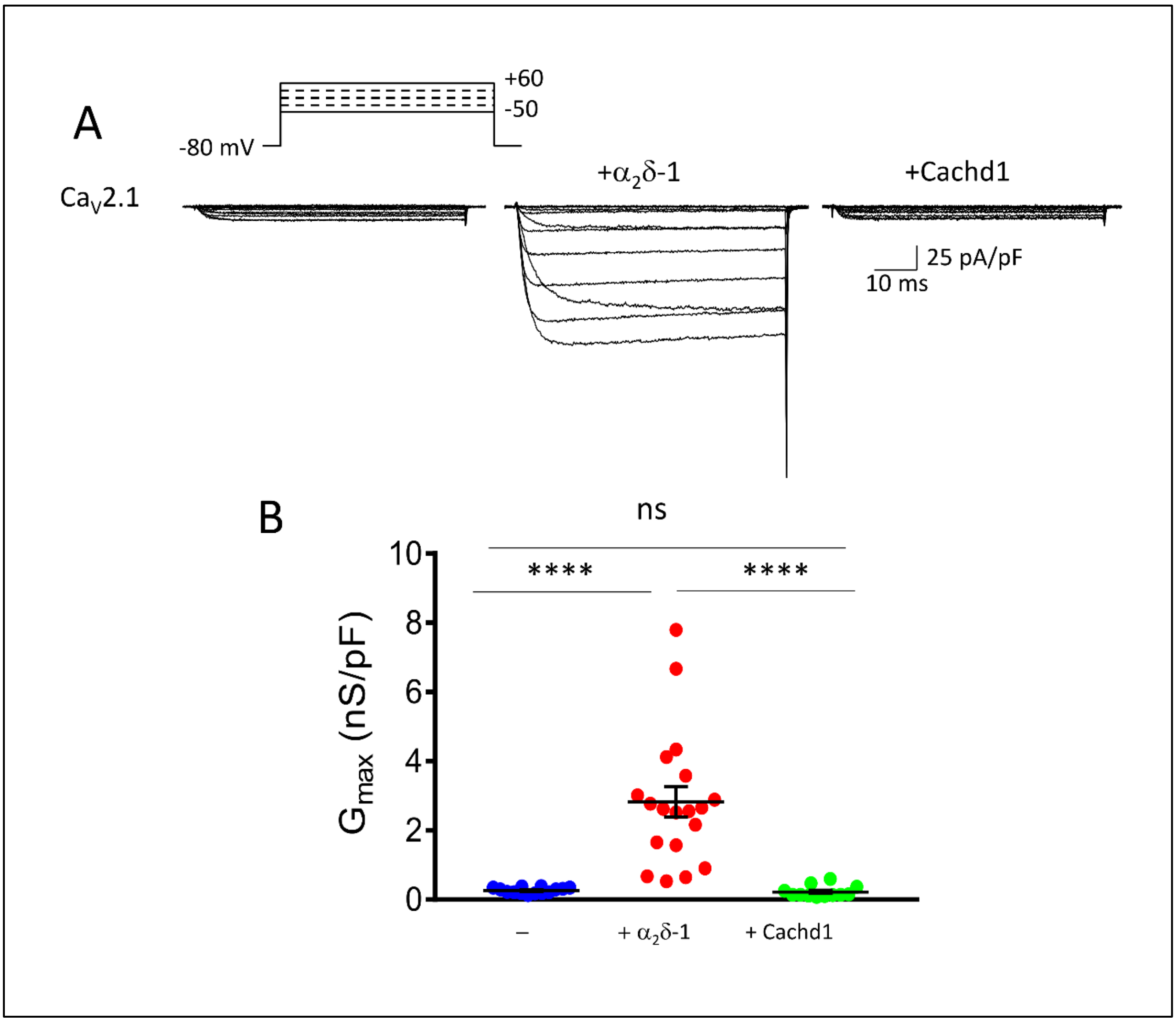

(A) Example current traces of $Ca_V2.1$ co-expressed with β1b and either no $\alpha_2\delta$ (left), $\alpha_2\delta$-1 (middle) or Cachd1 (right). Holding potential -80 mV, steps between -50 and +60 mV for 50 ms.

(B) Maximum conductance G_{max} (nS/pF) from the current-voltage relationships shown in Figure 2G. Individual data for $Ca_V2.1$ co-expressed with β1b and either no $\alpha_2\delta$ (blue solid circles), $\alpha_2\delta$-1 (red solid circles) or Cachd1 (green solid circles) and mean ± SEM are plotted. ns, not significant; **** P<0.0001 (one way ANOVA and Sidak's post-hoc test correcting for multiple comparisons). A similar result was observed for zCachd1 (data not shown).

Figure S4 (relates to Figure 3): $\alpha_2\delta$-1 and Cachd1 expression and effect on Ca_V2.2 cell surface expression

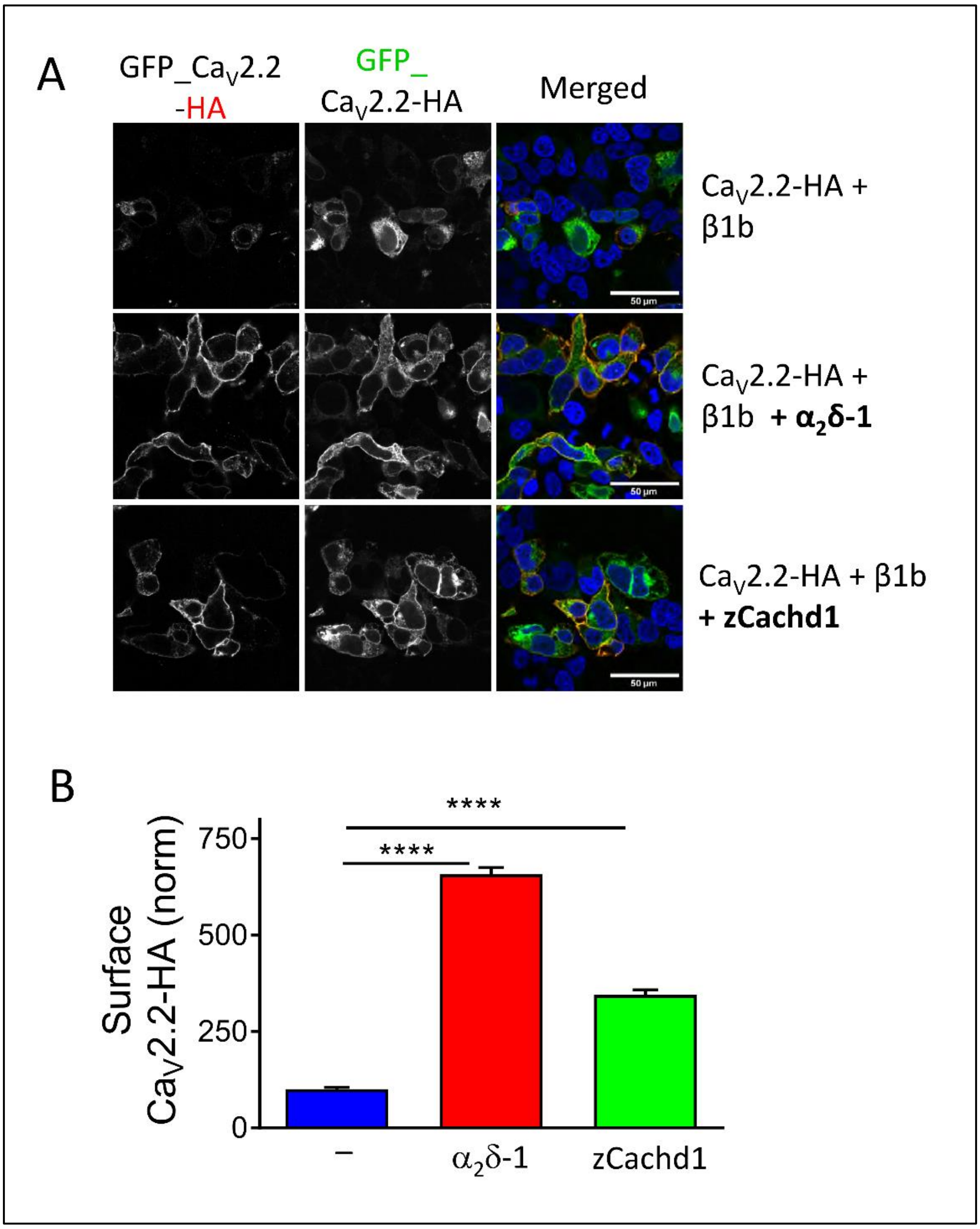

(A) Representative confocal images of tsA-201 cells expressing GFP_Ca_V2.2-HA WT with β1b in the absence of $\alpha_2\delta$ (control, top row) with $\alpha_2\delta$-1 (middle row) or zCachd1 (bottom row). Cells were not permeabilized and incubated with rat anti-HA antibody for 1 h to show extracellular HA staining on the plasma membrane (left panels, white), to be compared with intracellular GFP fluorescence (middle panels). Merged images (with HA in red) are shown in the right-hand panels; DAPI was used to stain the nuclei (blue). Scale bars are 50 µm.

(B) Bar chart showing cell surface expression of Ca_V2.2-HA, determined by HA staining in the absence of permeabilization. Control condition without $\alpha_2\delta$ or zCachd1 (blue, normalized to 100%), with $\alpha_2\delta$-1 (red) and zCachd1 (green). Data for 901 (- $\alpha_2\delta$-1), 921 (+ $\alpha_2\delta$-1), 970 (+ zCachd1) cells were normalized to the Ca_V2.2-HA condition in each experiment. **** $P<0.0001$ (1-way ANOVA and Sidak's post-hoc test correcting for multiple comparisons).
